# PI3K/Akt signalling pathway-associated long noncoding RNA signature predicts the prognosis of laryngeal cancer patients

**DOI:** 10.1038/s41598-023-41927-3

**Published:** 2023-09-07

**Authors:** Qian Nie, Huan Cao, JianWang Yang, Tao Liu, Baoshan Wang

**Affiliations:** Department of Otorhinolaryngology, The Second Hospital of Hebei Medical University, Hebei, 050000 China

**Keywords:** Biomarkers, Oncology, Risk factors

## Abstract

The *PI3K/Akt* signalling pathway is associated with the occurrence and development of tumours and significantly affects the prognosis of patients. We established a predictive signature based on the *PI3K/Akt* pathway to predict the prognosis of patients. The RNA-seq and clinical data of laryngeal cancer patients were downloaded from The Cancer Genome Atlas (TCGA) database. Three lncRNAs (*MNX1-AS1*, *LINC00330*, *LSAMP-AS1*) were selected through univariate, multivariate Cox and log-rank test analysis to establish a prognostic signature. The patients were then divided into high-risk and low-risk groups based on their risk score. In the TCGA training set, the survival time of the high-risk group was shorter than that of the low-risk group (P < 0.01). Follicular helper T cells were lower in the high-risk group (*P* = 0.022), and CCR, inflammation promotion, parainflammation, and type I IFN immune function were suppressed. The results of the drug sensitivity analysis suggest that the high-risk group is sensitive to AKT inhibitors. The establishment of the signature was also verified based on the clinical data. Three lncRNAs can facilitate the migration, invasion, and vitality of cancer cells in vitro, and vice versa. Moreover, *p-AKT* (Ser473) and *p-PI3K* were highly activated in the cells overexpressing the abovementioned three lncRNAs. The *PI3K/Akt* signalling pathway-associated prognosis signature has a good predictive effect.

## Introduction

The development of cancer cells is caused by an imbalance between apoptosis and growth, which mainly occurs due to the role of the *PI3K/AKt* signalling pathway. In a previous study, it was found that 76.47% of *AKT* was activated in tumour tissues, and the positivity rate in para-cancer tissues was only 38.46%^1^. The *PI3K/Akt* signalling pathway is the hub for regulating tumour cell apoptosis, cell growth, nutrient production, angiogenesis, metastasis, and substance metabolism. The most important aspect of the *PI3K/AKt* signalling pathway is the activation of *AKT* and the production of *p-AKT:* the inhibition of caspase-9, Bcl-2 and *TGF-β* lead to the inhibition of tumour cell apoptosis^2^; the activation of eNOS leads to increases in NO, migration and invasion rates^3^; and the activation of *mTOM* facilitates the synthesis of growth-related proteins, which leads to active tumour cell proliferation. Moreover, the *PI3K/AKt* signalling pathway can also regulate tumour metabolism. The intake of large amounts of glucose leads to increased insulin secretion among humans. Insulin binds to the cell surface receptor *IRS1* to activate *PI3K-Akt* and promote glucose absorption. Additionally, *P-AKT* activates *RheB* and *mTORC1*, thereby promoting glucose utilization and the synthesis of glycogen, which is stored in the body and maintains glucose concentration balance^4^. Amino acids are the only nutrients that can directly activate *mTORC1* and then decide whether to biosynthesize or decompose to provide energy for the body, depending on the condition of the body^4^. In summary, the *PI3K/AKt* signalling pathway plays an indispensable role in the occurrence and development of tumours. Therefore, many researchers have focused on inhibiting the *PI3K/Akt* signalling pathway to treat cancer. LncRNAs have been widely reported as important factors in gene regulation^5–7^.

The prognostic signature of lncRNAs related to the *PI3K/AKT* pathway is effective for predicting prognosis. However, to the best of our knowledge, there is no related discussion in previous research. Thus, it is necessary to establish an effective prognostic model and provide guidance for diagnosis and treatment. Therefore, we downloaded the RNA-seq data and clinical data of laryngeal cancer patients from TCGA, obtained the relevant lncRNAs of the *PI3K/Akt* signalling pathway through correlation analysis, and established a signature. Then, the rationality of the signature was verified by a clinical external verification set, and lncRNA function and activation of the PI3K/AKT pathway were verified by in vitro and Western blot assays, respectively**.**

## Materials and methods

### Data download and acquisition of PI3K/Akt signalling pathway-related genes

We downloaded RNA-seq and clinical data of laryngeal cancer patients from GDC (http://portal.gdc.cancer.gov/). There were 111 tumour samples and 12 normal samples. Table 1 lists the basic information of the TCGA samples. The “LIMMA” package in R software (v.4.0.2, http://www.r-project.org) was utilized to analyse the differentially expressed lncRNAs and mRNAs between tumour and normal tissues^8^. The DEGs (differentially expressed genes) were screened based on a false discovery rate (FDR) < 0.05 and |log2FoldChange| > 1, including 443 DElncRNAs (differential expression lncRNAs) and 908 DEmRNAs (differential expression mRNAs). A total of 346 mRNAs related to PI3K/AKT were obtained from the CUSABIO website (http://www.cusabio.com/pathway.html). Among them, 30 mRNAs were differentially expressed in laryngeal cancer. The clinical data originated from the Institute of Otorhinolaryngology, Hebei Medical University. The inclusion criteria were as follows: (1) patients diagnosed with laryngeal cancer; (2) patients selecting treatment under informed consent and agreeing to follow-up; and (3) patients who had not undergone radiotherapy and chemotherapy. In accordance with the inclusion criteria, 52 patient samples (52 cancer tissue samples and normal tissue samples) were included from October 2016 to November 2018. Seven patients were lost to follow-up, and thus, 45 patients had complete follow-up data. The average follow-up duration was 52 ± 9 months. All patients were male. Table 1 lists the details of the patients.

**Table 1:** Basic clinical information of patients.

	TCGA dataset (n = 111)	Clinical dataset (n = 45)
Age	62.87 ± 9.25	62.31 ± 7.80
Male	91 (81.98%)	45 (100%)
Stage		
I	3 (2.70%)	10 (22.22%)
II	11 (9.91%)	7 (15.56%)
III	27 (24.32%)	13 (28.89%)
IV	70 (63.06%)	15 (33.33%)
T		
T1	6 (5.41%)	12 (26.67%)
T2	15 (13.51%)	13 (28.89%)
T3	36 (32.43%)	12 (2.22%)
T4	54 (48.65%)	8 (17.78%)
N		
N0	56 (50.45%)	28 (62.22%)
N1	19 (17.12%)	8 (17.78%)
N2	31 (27.93%)	9 (12%)
N3	3 (2.70%)	0
Nx	2 (1.80%)	0
M		
M0	110 (99.10%)	44 (97.78%)
M1	1 (0.90%)	0
Mx	0	1 (2.22%)

*T* tumor, *M* metastasis, *N* lymph node.

### Establishment of a PI3K/Akt signalling pathway-related lncRNA predictive signature

A total of 443 DElncRNAs and 30 DEmRNAs related to the PI3K/AKT pathway were examined via Spearman correlation analysis in R^9^. According to the criteria of |Correlation coefficient| > 0.4 and P value < 0.001, 325 DElncRNAs related to the PI3K/AKT pathway were screened. Subsequently, the selected lncRNAs were studied through univariate Cox and log-rank tests by the “survival” and “survminer” packages^10,11^. A P value < 0.01 was used as the screening criterion. A total of 6 lncRNAs associated with the survival status of laryngeal cancer patients were screened (including *AP001065.15, LSAMP-AS1, LINC00330, MNX1-AS1, RP11-190J1.3*, and *AC005281.2*). Finally, the *PI3K/Akt* signalling pathway-associated prognostic signature of laryngeal cancer patients was established by multivariate Cox analysis^12^. In accordance with the results of the multivariate Cox regression model, the risk score is expressed as:

$${\text{Risk score}} = {\text{h}}_{0} (t) * \exp \left\{ {\sum\limits_{i = 1}^{n} {\left( {{\text{a}} \times {\text{x}}_{{\text{i}}} } \right)} } \right\}$$

(a: the coefficient value of each factor in the results of multivariate analysis and x_i_ represents the expression of 3 lncRNAs in each sample of laryngeal cancer patients)^13^.

The risk score of 111 cancer samples was calculated. See Supplementary Table S1 for details. With the increase in the risk score, the samples were divided into two groups based on the median risk score: a high-risk group and a low-risk group^14^.

### Establishment of the multivariate Cox regression model and nomogram

We established a multivariate Cox regression model and nomogram based on age, stage, and three lncRNAs (*LINC00330*, *MNX1-AS1*, *LSAMP-AS1*). The nomogram was created using the "rms" package^15^. The validity of the multivariate Cox regression model was verified by the C-index, and the validity of the nomogram was verified by the receiver operating characteristic curve (ROC), univariate Cox analysis, multivariate Cox analysis, and the correction curve^16^.

### Clinical data as external verification of the signature

Forty-five patients in our centre were used as the external verification set of the predictive model. The expression levels of 3 genes (*LINC00330*, *MNX1-AS1*, *LSAMP-AS1*) in 45 laryngeal cancer tissues and matched normal tissues were obtained by real-time PCR, and the relative expression was calculated. Finally, age and stage were combined to establish a multivariate Cox regression model and nomogram to verify the applicability of the model.

### Immune infiltration and drug sensitivity analysis

Single-sample gene set concentration analysis (ssGSEA) in the gene set variation analysis (GSVA) package was used to analyse immune infiltration in the two subgroups^17^. Moreover, the difference in the 50% inhibitory concentration (IC50) of commonly used chemotherapeutic drugs in the high- and low-risk groups was also analysed by the rank sum test (IC50). The IC50% of the drug reaction was calculated by the “prophetic” package^18^.

### Cell culture

Laryngeal cancer cell lines (TU177, AMC-HN-8) were purchased from Beina Chuanglian Institute of Biotechnology in Beijing. The cells were cultured in RPMI 1640 or DMEM with 10% foetal bovine serum and cultured at 37 °C and 5% CO_2_.

All the culture media mentioned above were obtained from GIBCO 108 (Thermo Fisher Science, Inc.)

### Quantitative real-time polymerase chain reaction

The total RNA of the paired tissues of 45 patients was extracted with an EaStep® Super Total RNA extraction kit (Promega, USA), and then the total RNA was reversed transcribed with a Transcriptor First Strand cDNA Synthesis Kit (Roche, Germany). Finally, quantitative real-time polymerase chain reaction (qRT‒PCR) was performed by GoTaq ®qPCR Master Mix (Promega, USA). The relative expression of the gene was calculated by 2^−∆∆Ct^ (GAPDH as reference).

The sequences of the primers used in the experiment are as follows:

- GAPDH (forwards): 5ʹ-AGGTGAAGGTCGGAGTCAACG-3ʹ.
- GAPDH (reverse): 5ʹ-AGGGGTCATTGATGGCAACA-3ʹ.
- LSAMP-AS1 (forwards): 5ʹ-GCAAAGGCTCAGAGGGATGC-3ʹ.
- LSAMP-AS1 (reverse): 5ʹ-AGTTTTCACCTGTAGGGAGCA-3ʹ.
- MNX1-AS1 (forwards): 5ʹ-CCAAAGCTCTGCAGGTCGAA-3ʹ.
- MNX1-AS1 (reverse): 5ʹ-AGACTCACGTAGCACTGTGG-3ʹ.
- LINC00330 (forwards): 5ʹ-ACAGTTGAGAACAGACTCCACC-3ʹ.
- LINC00330 (reverse): 5ʹ-TGAGGTAAAGTTGCTCGGGG-3ʹ.

### Cell transfection

TU177 and AMC-HN-8 were cultured in a 6-well plate, and when the fusion rate reached 90%-95%, 4 μg of the overexpression of *LSAMP-AS1*, *MNX1-AS1*, and* LINC00330* (GENEWIZ, China)/5 μl of antisense oligodeoxynucleotides (ASOs) of *LSAMP-AS1*, *MNX1-AS1*, and* LINC00330* (RIBOBIO, China) were transferred into the abovementioned cells in combination with 7.5 μg of Hieff Trans Liposomal Transfection Reagent (Yeasen Biotechnology, Shanghai), with pcDNA3.1 vector (Invitgen, USA) as the control group. After 48 h, the follow-up experiment was carried out.

### Cell proliferation assays

After digestion, the transfected TU177 and AMC-HN-8 cells were cultured in a 96-well plate at a quantity of 2 × 10^3^ cells/well, and each index was repeated three times. Then, 20 μl of MTS (CellTiter96 ®AQueousOne Solution Cell Proliferation Analysis Kit) (Promega, USA) was added to a 96-well plate and cultured for 2.5 h. The optical density (OD) was examined at 490 nm with a Spark ®143 multimode microplate reader (Mod: Spark 10 m Tecan, Switzerland), and the cell proliferation activity was examined at 0 h, 24 h, 48 h and 72 h, respectively.

### Transwell and migration assays

According to previously reported methods, cell migration and invasion were examined by Transwell assays (Corning Company, USA)^19^. In the invasion assay, 50 μl of Matrigel was added to the chamber advance and incubated for 30 min in an incubator at 37 °C. Subsequently, for the migration and invasion assay, cells were inoculated with 100 μl of nonfoetal bovine serum medium (1 × 10^5^ cells/well) in the upper chamber and then inoculated with 650 μl of medium containing 10% foetal bovine serum in the lower chamber. The cells were cultured for 24 h, washed with PBS for 20 min, and fixed with 4% paraformaldehyde for 20 min. Then, 0.5% crystal violet staining was performed on the cells for 20 min. Finally, the number of migrated or invaded cells was observed by microscopy (CKX53, Olympus).

### Wound healing assay

According to a previous report, we spread the cells on a six-well plate, transfected the plasmid for 48 h, and drew lines along the straight ruler with a sterile 200-μl pipette tip perpendicular to the cell surface^19^. The scratches were washed with aseptic PBS 3 times and added to the culture medium. The six-well plate incubator was cultured. Subsequently, the cells were removed at 0 h, 24 h, and 48 h, and the width of the scratch was measured and photographed under a microscope (CKX53; Olympus).

### Colony formation assay

The transfected cells were inoculated in a six-well plate at 2 × 10^3^ cells/well and then cultured in an incubator at 37 °C and 5% CO_2_ for 10 days. Next, the cells were fixed with 4% paraformaldehyde for 20 min, and 0.5% crystal violet staining was performed on the cells for 20 min. The number of clones formed in the six-well plate was calculated.

### Western blot

The six-well plates transfected with MNX1-AS1, LINC00330, LSAMP-AS1 overexpression plasmid and pcDNA3.1 as the control group for 48 h were collected, and the cells were lysed with RIPA lysate (Solarbio, China), PMSF (SolarBio, China) and protease inhibitor (Promega, USA). The cell protein cleavage products were mixed with SDS‒PAGE loading buffer (Solarbio, China) at 4:1 and then boiled in a metal bath at 95 °C for 5 min. Moreover, an equal amount of (25 μg) protein mixture was added to a 10% sodium dodecyl sulfate‒polyacrylamide gel electrophoresis plate (SDS‒PAGE) for electrophoresis. The gel was cut near the corresponding molecular weight of the target protein, transferred to a polyvinylidene fluoride membrane, incubated with 5% milk for 2 h, incubated with the first antibody of the target protein overnight, and then incubated with the corresponding second antibody for 2 h the next day. The primary antibodies were GAPDH (1:10,000, Cat No. 60004-1-1 g, Proteintech, China), AKT (pan) (1:1000, 4691, CST, USA), p-AKT (Ser473) (1:1000, 4060, CST, USA), PI3K (p85) (1:1000, EM1701-62, HUABIO, China) and p-PI3K (P85, Tyr467/Tyr199) (1:1000, GTX132597, GENETEX, USA). The secondary antibody was anti-rabbit IgG (Haul) (1 10,000, Cat No. SA00001-2, Proteintech, China), and chemiluminescence was displayed by fluorescent XRS + (Bio-Rad, USA).

### Ethics approval and consent

The data of this study were downloaded from the open database. Cancer tissues and paracancerous tissues were obtained from patients with laryngeal cancer at the Second Hospital of Hebei Medical University. All experimental protocols were approved by the Research Ethics Committee of the Second Hospital of Hebei Medical University. Informed consent was obtained from all patients. This study was conducted in accordance with the guidelines of the Declaration of Helsinki.

## Results

### Acquisition of *PI3K/Akt* signalling pathway-associated lncRNAs

**Figure 1:**
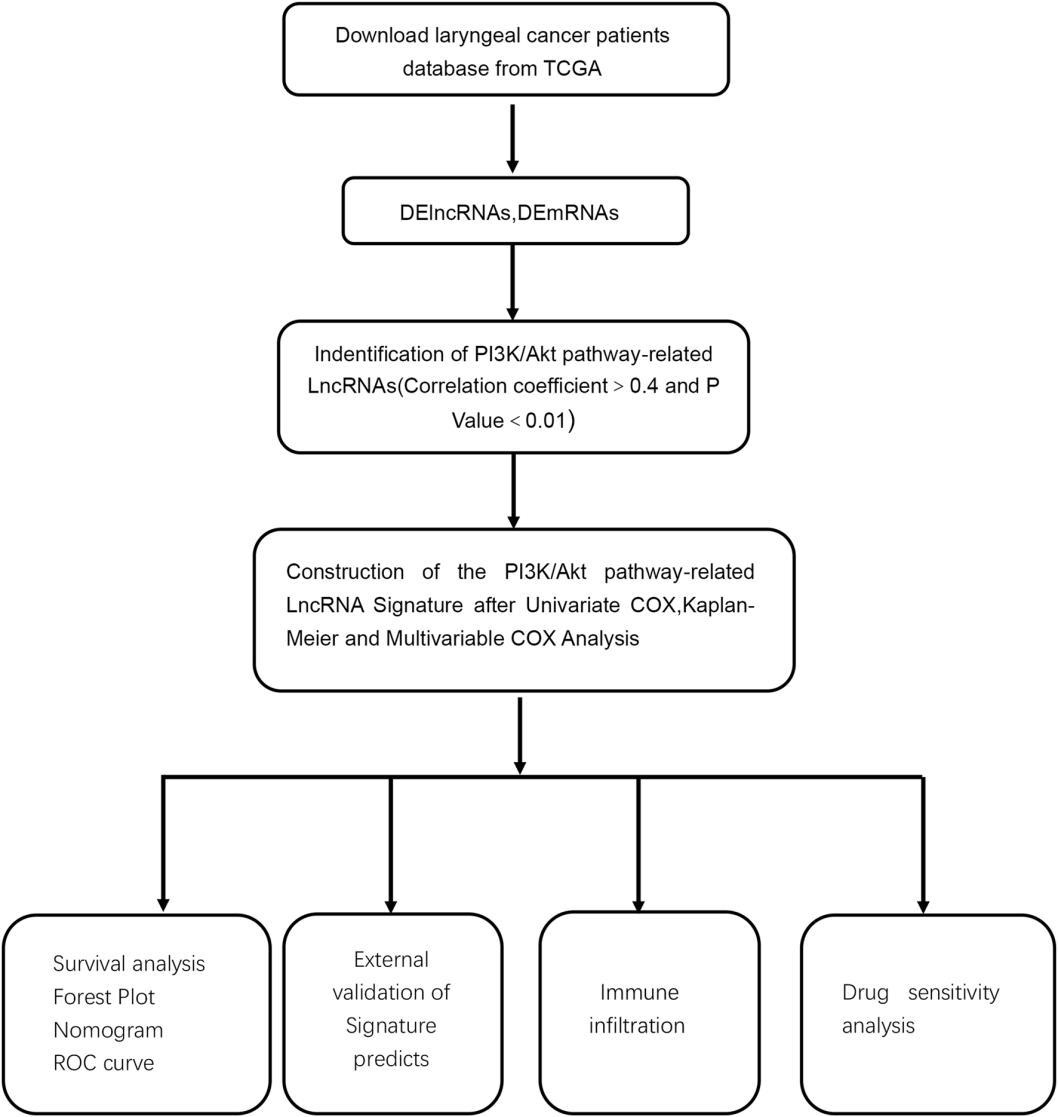
The flowchart of our research. *TCGA* The Cancer Genome Atlas, *DElncRNAs* Differentially expressed lncRNAs, *DEmRNAs* Differentially expressed mRNAs, *lncRNA* long noncoding RNA, *ROC* Receiver operating characteristic.

**Figure 2:**
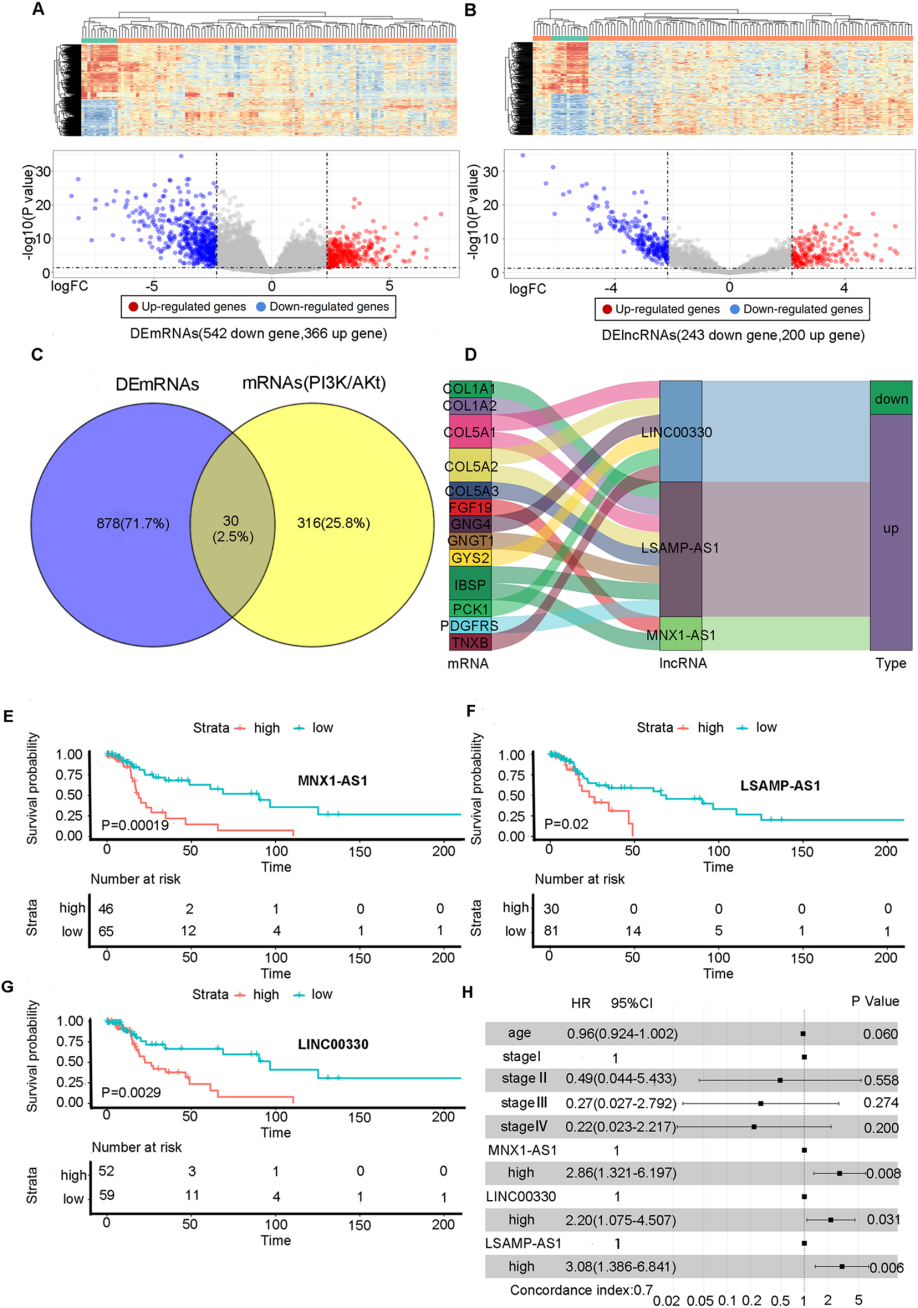
Differential gene screening (**A**) Differentially expressed lncRNAs in laryngeal carcinoma tissues and normal paired tissues, (**B**) Differentially expressed mRNAs in laryngeal carcinoma tissues and normal paired tissues, (**C**) The intersection of differentially expressed mRNA in laryngeal carcinoma tissues and mRNA related to PI3K/Akt pathway; (**D**) The mulberry diagram of mRNA related to PI3K/Akt pathway and coexpressed lncRNA. (**E**–**G**) Kaplan–Meier survival curve of MNX1-AS1,LSAMP-AS1 and LINC00330 in high and low expression subgroup (**H**) Multivariate Cox regression analysis of pathological characteristics of patients and the expression 3 lncRNAs. *HR* Hazard Ratio, *CI* confidence interval.

We downloaded RNA-seq and clinical data of laryngeal cancer patients from GDC. There were 123 samples (111 cancer tissues and 12 normal tissues). The general idea of the research can be seen in Fig. 1. Analysis of DEmRNAs and DElncRNAs between cancer and normal tissues was performed by using the "limma" package of R software. The results of the packages were DEmRNAs: 908 (upregulated mRNAs: 366, downregulated RNAs: 542) and DElncRNAs: 443 (upregulated lncRNAs: 200, downregulated lncRNAs: 243) (Fig. 2A,B). Correlation analysis between 443 DElncRNAs and 30 DEmRNAs related to the PI3K/Akt signalling pathway (Fig. 2C) was performed. Using |Correlation coefficient| > 0.4 and P value < 0.001 as the screening criteria, a total of 325 *PI3K/Akt* signalling pathway-associated lncRNAs were screened. The lncRNAs were studied through univariate Cox and log-rank test analyses. With a P value < 0.01 as the threshold, a total of six lncRNAs (*AP001065.15, LSAMP-AS1*, *LINC00330*, *MNX1-AS1*, *RP11-190J1.3*, *AC005281.2*) showed positive significance in the two tests (Tables 2 and 3). Three lncRNAs (*MNX1-AS1*, *LSAMP-AS1* and *LINC00330*) were selected through multivariate analysis to establish a prognostic signature. The coexpression relationship between the three lncRNAs and the DEmRNAs related to PI3K/AKt signalling pathways is shown in Fig. 2D.

**Table 2:** Univariate cox regression.

	Coef	P value	HR	95% CI
LSAMP-AS1	− 1.386	0.001	0.25	0.114–0.546
MNX1-AS1	− 1.331	0.001	0.264	0.125–0.559
AC005281.2	− 1.48	0.001	0.228	0.097–0.535
RP11-190J1.3	1.323	0.002	3.753	1.614–8.726
FAM225B	− 1.153	0.004	0.316	0.143–0.698
LINC00330	− 1.037	0.005	0.355	0.171–0.734
AP001065.15	− 0.985	0.007	0.374	0.182–0.768

*Coef* Regression coefficient, *HR* Hazard Ratio, *CI* confidence interval.

**Table 3:** Log-rank test.

Gene	P value
AC005281.2	0.000468735
MNX1-AS1	0.000524004
LINC00330	0.002917935
LSAMP-AS1	0.003769397
RP11-190J1.3	0.00500349
RP11-567M16.1	0.006125463
AP001065.15	0.006366346

### Verification of the validity of the lncRNA prognostic signature

**Figure 3:**
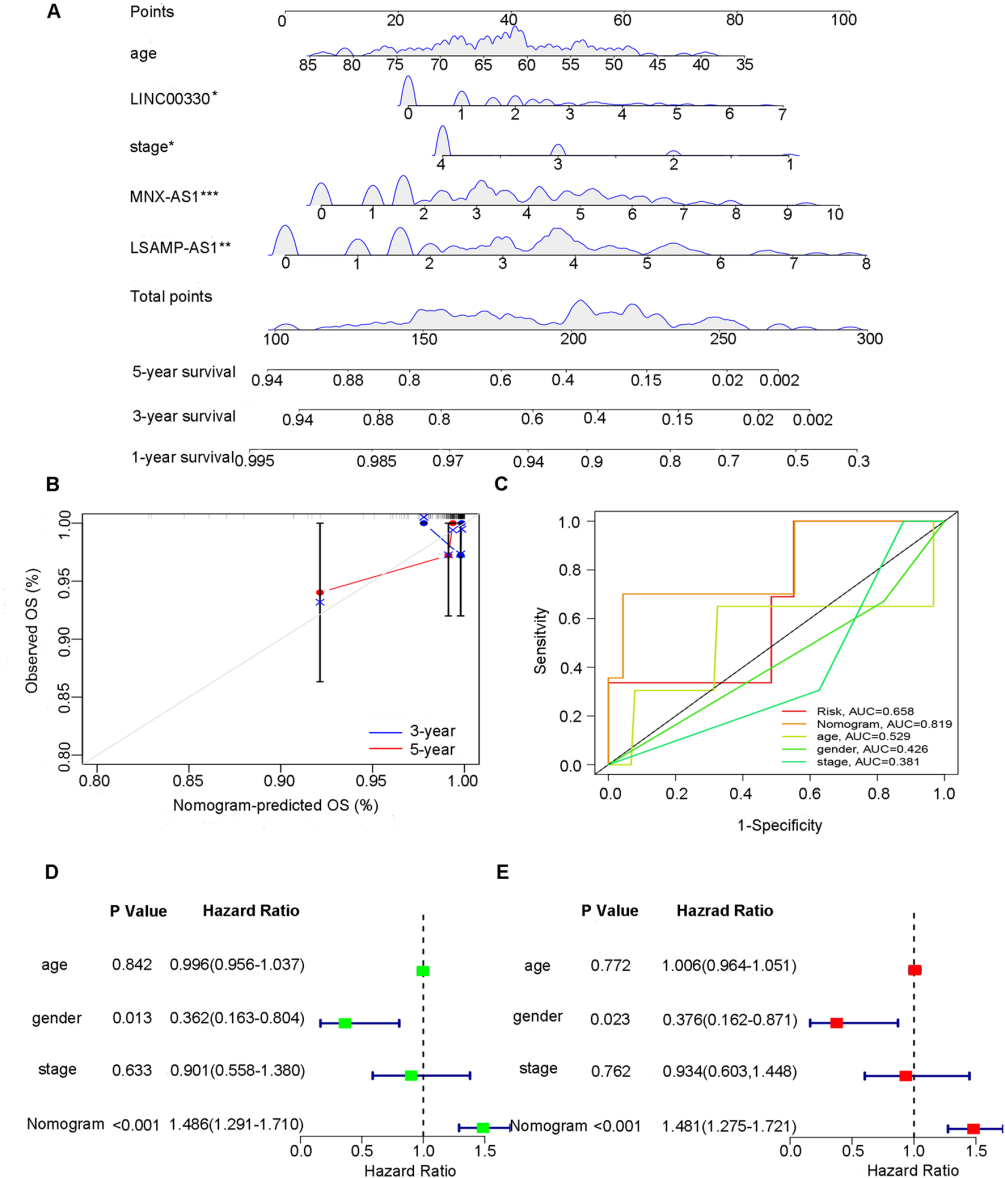
Construction and verification of Nomogram. (**A**) Construction of Nomogram combining 3 lncRNAs expression and clinical variables; (**B**) Consistent evaluation of actual survival rate and Nomogram predicted survival rate of patients; (**C**) Comparison of specificity and sensitivity of risk score, Nomogram score and clinical variables in predicting survival status of patients; (**D**, **E**) Forest Plot for univariate and multivariate Cox regression analysis.

*MNX1-AS1*, *LSAMP-AS1* and *LINC00330* are independent prognostic factors (Fig. 2E–G). The expression of *MNX1-AS1*, *LSAMP-AS1* and *LINC00330* was combined with age and stage to establish a prognostic signature. *MNX1-AS1* (*P* = 0.00769, hazard ratio = 2.8610), *LINC00330* (P = 0.03091, hazard ratio = 2.2013) and *LSAMP-AS1* (*P* = 0.00577, hazard ratio = 3.0786) were strongly correlated with patient survival. The establishment of the multivariate Cox regression model was credible (concordance index: 0.7) (Fig. 2H). The survival time of patients was shortened, and the number of deaths increased (Supplementary Fig. S1A). Additionally, the survival time of high-risk patients was significantly shorter than that of low-risk patients (*P* < 0.01) (Supplementary Fig. S1B). Moreover, the above factors were adopted to establish the nomogram to predict the 1-year, 3-year, and 5-year survival probability of the patients (Fig. 3A). The validity of the nomogram was verified by generating correction curves, ROC curves, and univariate and multivariate Cox regression models. The correction curve shows a high coincidence between the survival status of the nomogram and the actual survival status of patients at 3 and 5 years (Fig. 3B). The ROC curve indicated that the sensitivity and specificity of the nomogram prediction were higher than those of risk score, age, sex, and stage (area under the curve = 0.819) (Fig. 3C). Age, sex, stage, and the nomogram score were studied through univariate Cox regression analysis, and all factors were integrated for multivariate Cox regression (Fig. 3D,E). The results suggest that the nomogram score is an independent prognostic factor (*P* < 0.001, hazard ratio = 1.486). The results of the multivariate Cox regression analysis also suggested that the nomogram score was significantly correlated with patient prognosis (*P* < 0.01, hazard ratio = 1.481). The nomogram score was significantly correlated with prognosis (*P* < 0.01).

### External verification of the lncRNA prognostic signature

**Figure 4:**
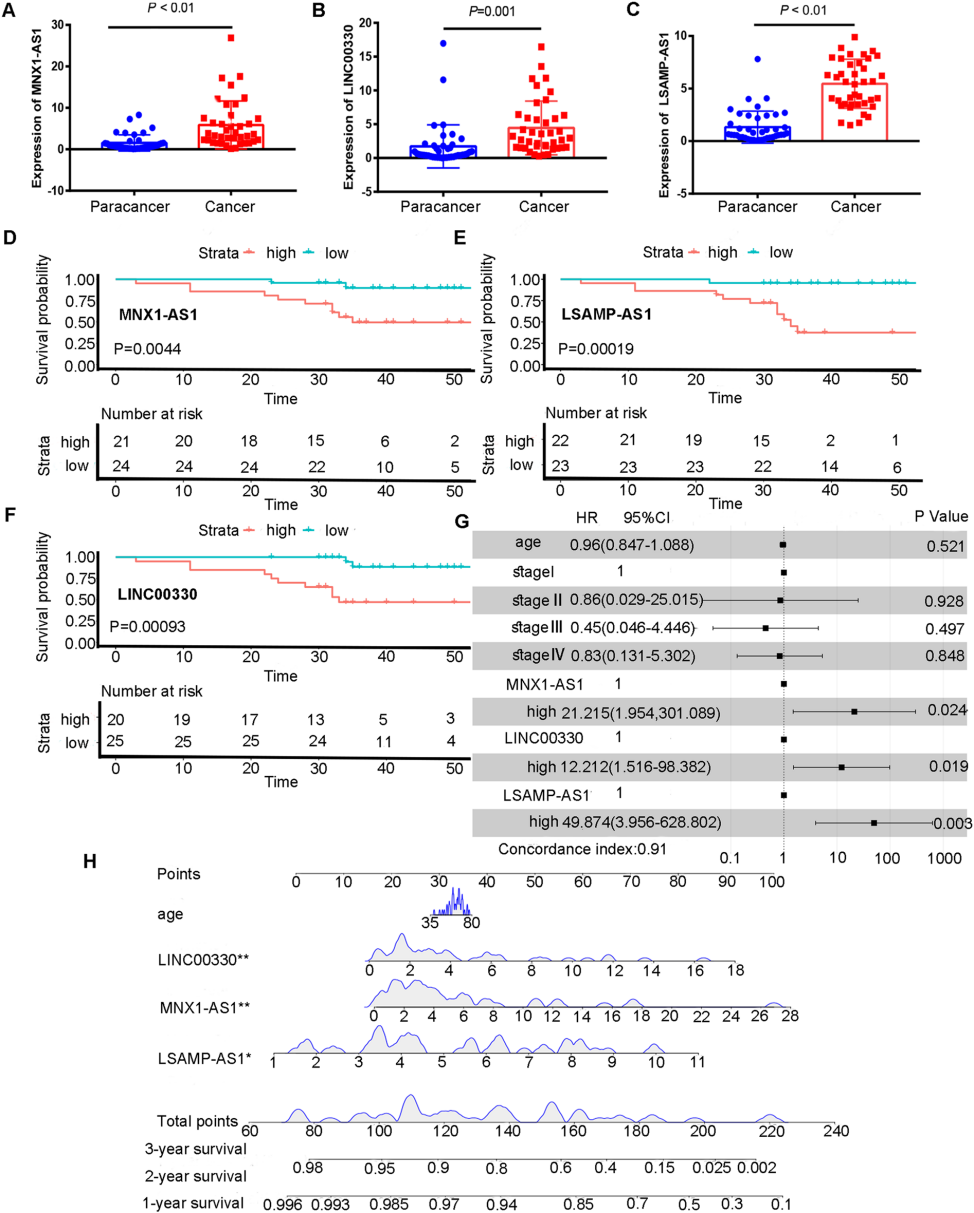
Verification of clinical external verification set. (**A**–**C**) Expression of MNX1-AS1, LINC00330 and LSAMP-AS1 in cancer and paracancerous tissues of 45 clinical patients. (**D**–**F**) Kaplan–Meier survival curve of MNX1-AS1, LSAMP-AS1 and LINC00330 in high and low expression subgroup in clinical external verification set. (**G**) Multivariate Cox regression analysis of pathological characteristics of clinical patients and the expression 3 lncRNAs (**H**) Construction of Nomogram combining 3 lncRNAs expression and clinical variables in clinical external verification set. *HR* Hazard Ratio, *CI* confidence interval.

We calculated the relative expression of *MNX1-AS1, LSAMP-AS1* and *LINC00330* in 45 pairs of laryngeal cancer patients (45 cancer tissue samples and 45 normal samples). The expression of *MNX1-AS1*, *LINC00330* and *LSAMP-AS1* was high in laryngeal carcinoma (Fig. 4A–C). In the survival analysis of the three lncRNAs, it was found that the results were highly consistent with those of the above signature, and *MNX1-AS1, LSAMP-AS1* and *LINC00330* could be used as prognostic factors (Fig. 4D–F). We combined the expression of *MNX1-AS1, LSAMP-AS1, LINC00330*, age, and stage to establish a nomogram. The results of the multivariate regression analysis (Fig. 4G) and the nomogram model (Fig. 4H) indicate that 3 lncRNAs are independent prognostic factors. According to the above methods, the clinical patients were divided into high- and low-risk groups, and the survival time of the high-risk group was significantly shorter than that of the low-risk group (P < 0.01). (Supplementary Fig. S2).

### Analysis of immune infiltration and drug sensitivity

**Figure 5:**
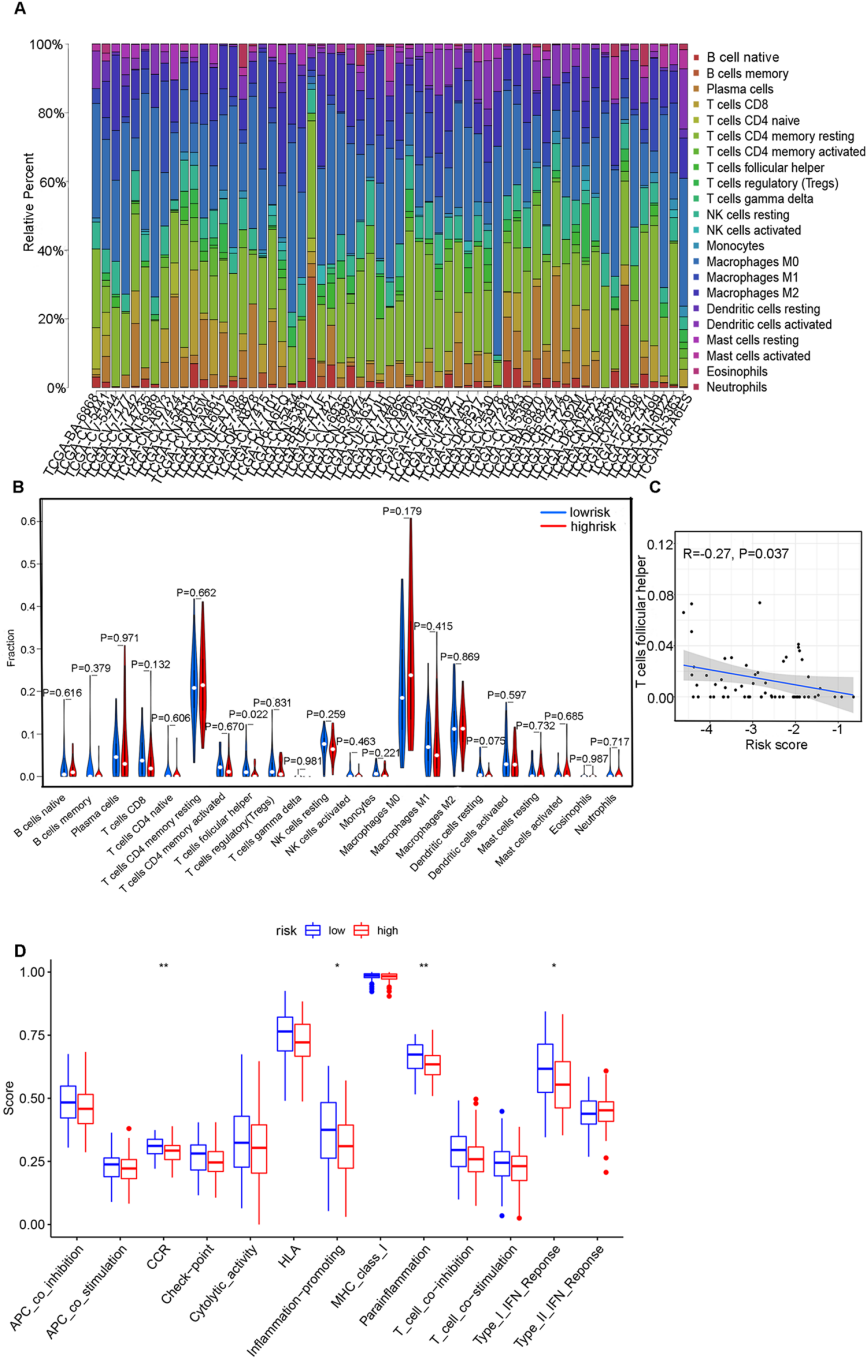
Immune cell infiltration in TCGA patients with laryngeal carcinoma. (**A**) Infiltration of immune cells in laryngeal cancer samples of patients; (**B**) Difference of immune cell infiltration between high and low risk subgroups; (**C**) Correlation Analysis between T cells follicular helper and risk score. (**D**) Difference of immune function between high and low risk subgroups.

**Figure 6:**
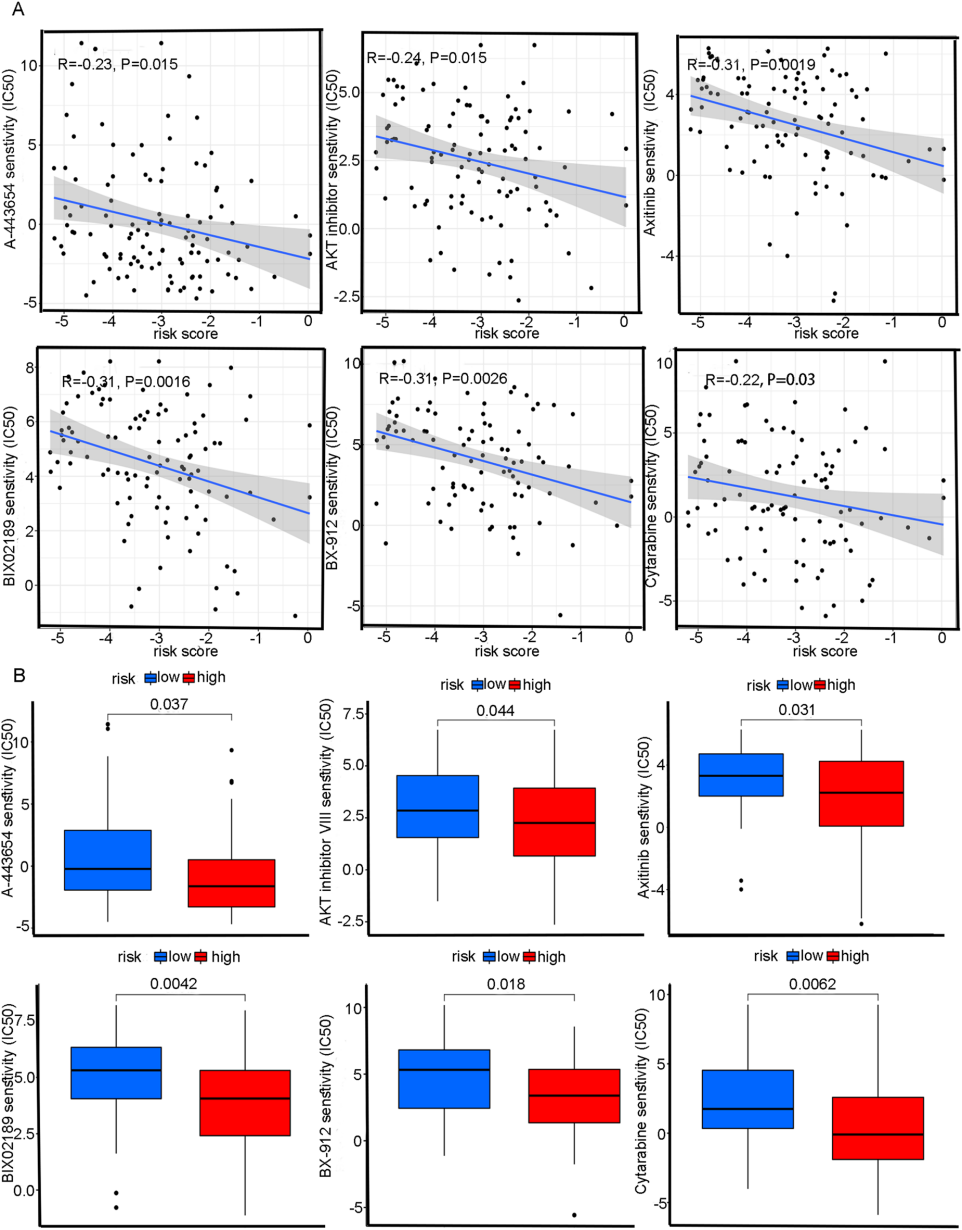
Comparison of drug sensitivity between high and low risk subgroups (**A**) Correlation analysis of A-443654, AKT inhibitor VIII, Axitinib, BIX02189, BX-912, Cytarabine and risk score. (**B**) The IC50 of A-443654, AKT inhibitor VIII, Axitinib, BIX02189, BX-912 and Cytarabine in high-risk group and low-risk subgroup.

To explore the molecular mechanism of high- and low-risk groups and the relationship between gene function and immunity. The immunity of the patient can be seen in Fig. 5A. The risk score with immune cell infiltration and immune cell function was analysed. Different immune cells were quantified in the process of analysis of the risk score. Follicular helper T cells were lower in the high-risk group (*P* = 0.022), and the risk score was negatively correlated with follicular helper T cells (R = − 0.27, P = 0.037) (Fig. 5B,C). Additionally, CCR, inflammation promotion, parainflammation, and type I IFN immune function were suppressed (Fig. 5D). We also analysed the drug sensitivity of the patients in the high-risk group. The IC50 values of A-443654, AKT inhibitor VIII, BX-912, BIX02189, axitinib, and others in the high-risk group were lower (P < 0.05) (Fig. 6A,B). The results provide guidance for the design of specific drug treatments for the high-risk group.

### lncRNA function analysis

**Figure 7:**
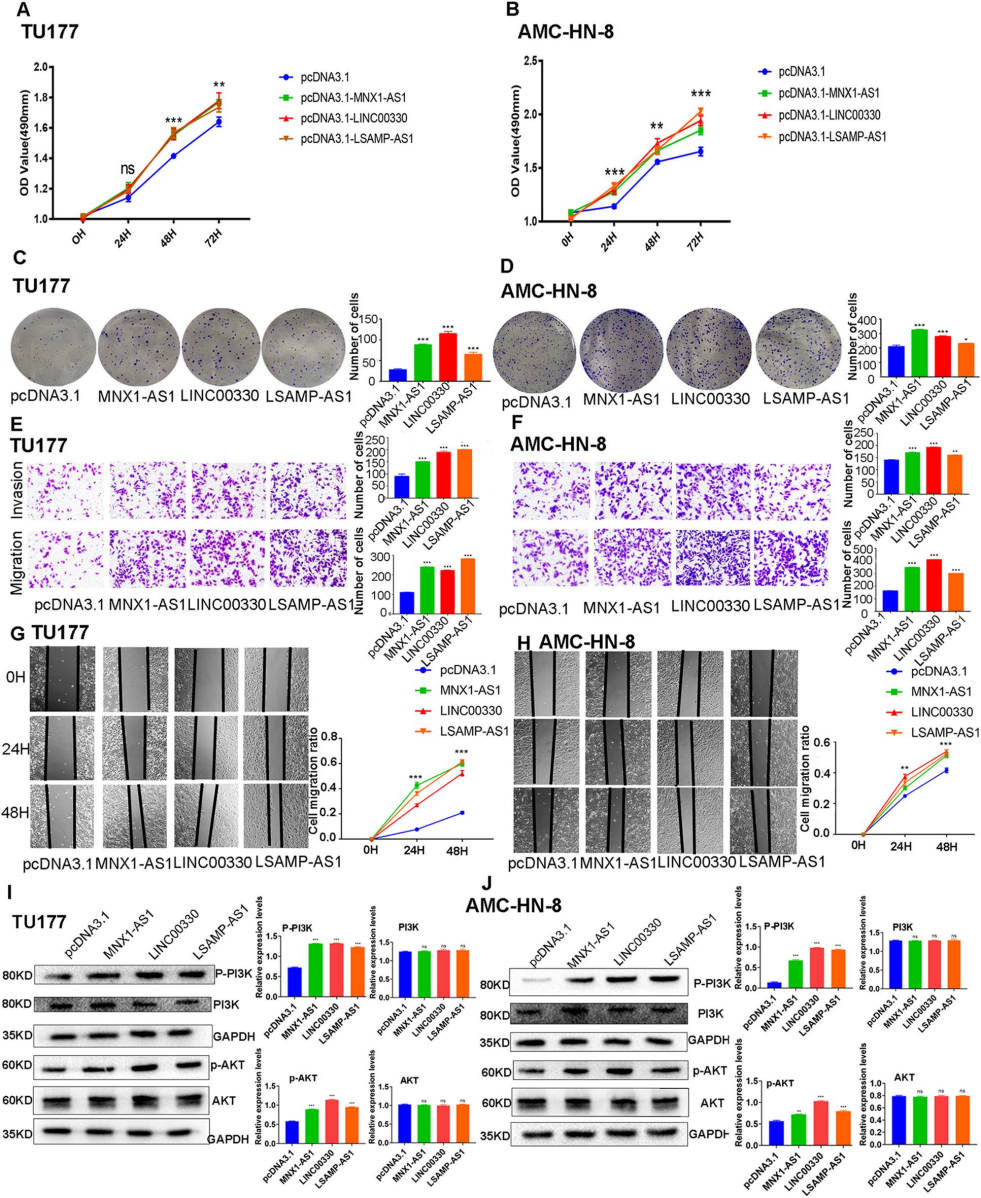
The overexpression function of 3 lncRNAs and the verification of of PI3K/Akt pathway. (**A**) Comparison of viability in TU177 cells between MNX1-AS1, LINC00330, LSAMP-AS1 overexpression group and pcDNA3.1 group; (**B**) Comparison of viability in AMC-HN-8 cells between MNX1-AS1, LINC00330, LSAMP-AS1 overexpression group and pcDNA3.1 group; (**C**) Comparison ability of colony formation in TU177 cells between MNX1-AS1, LINC00330, LSAMP-AS1 overexpression group and pcDNA3.1 group; (**D**) Comparison ability of colony formation in AMC-HN-8 cells between MNX1-AS1,LINC00330,LSAMP-AS1 overexpression group and pcDNA3.1 group; (**E**) Comparison ability of migration and invasion in TU177 cells between MNX1-AS1, LINC00330, LSAMP-AS1 overexpression group and pcDNA3.1 group; (**F**) Comparison ability of migration and invasion in AMC-HN-8 cells between MNX1-AS1, LINC00330, LSAMP-AS1 overexpression group and pcDNA3.1 group; (**G**) Comparison ability of cell migration in TU177 cells between MNX1-AS1, LINC00330, LSAMP-AS1 overexpression group and pcDNA3.1 group; (**H**) Comparison ability of cell migration in AMC-HN-8 cells between MNX1-AS1, LINC00330, LSAMP-AS1 overexpression group and pcDNA3.1 group (**I**) Western Blot analysis of p-PI3K, p-AKT, PI3K and AKT activation situation of MNX1-AS1, LINC00330, LSAMP-AS1 overexpression group and pcDNA3.1 in TU177 cells (Quantify the results of blots by Image J, and calculate the relative protein amount of the target protein relative to the internal reference protein(GAPDH)); (**J**) Western Blot analysis of p-PI3K, p-AKT, PI3K and AKT activation situation of MNX1-AS1, LINC00330, LSAMP-AS1 overexpression group and pcDNA3.1 in AMC-HN-8 cells (The original images of all gels has been cropped, and the above original images can be seen in the Supplementary File).

**Figure 8:**
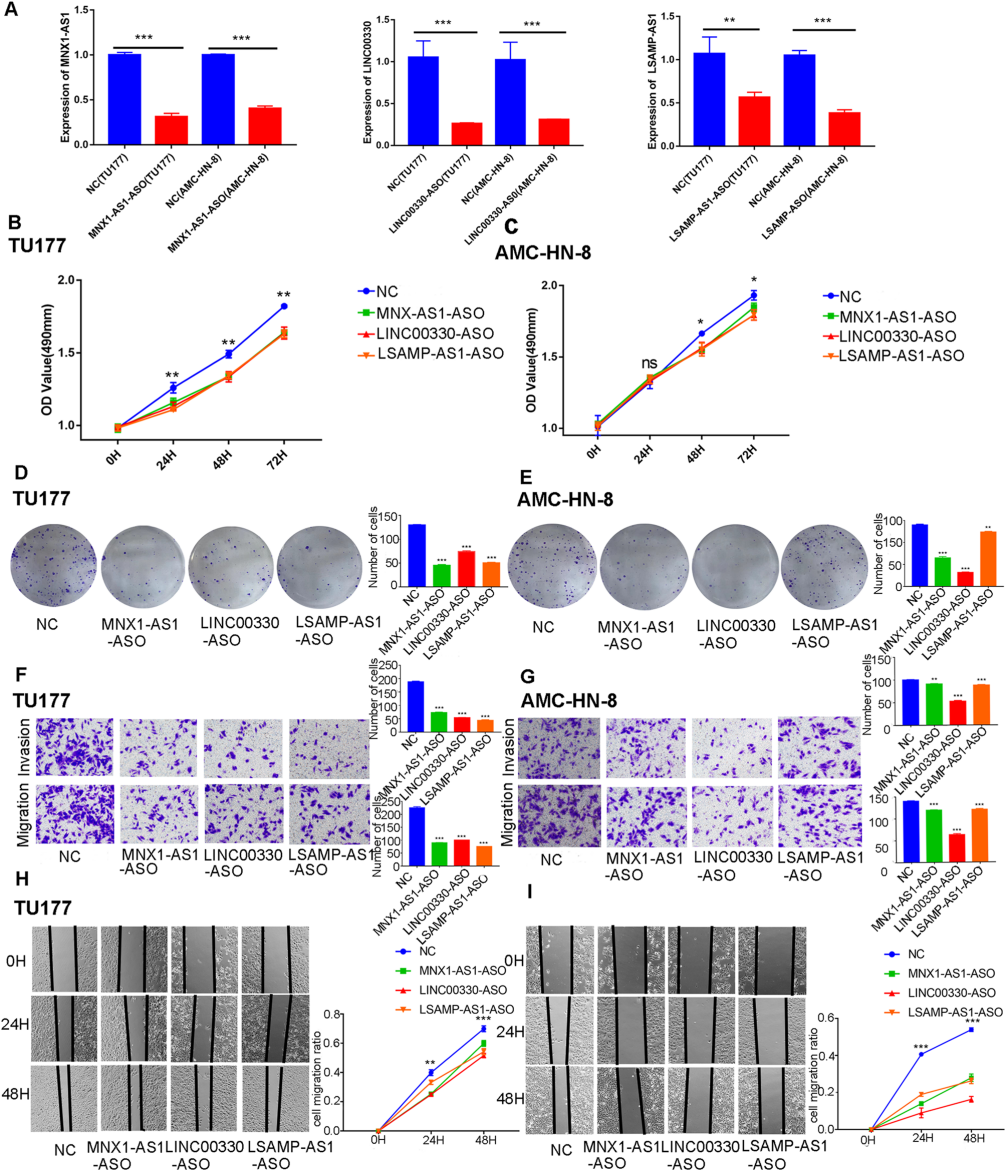
The knockdown function of 3 lncRNAs: (**A**) Validation of 3 lncRNAs knockdown efficiency in TU177 and AMC-HN-8 cells (**B**) Comparison of viability in TU177 cells between MNX1-AS1, LINC00330, LSAMP-AS1 knockdown group and non-specific control group; (**C**) Comparison of viability in AMC-HN-8 cells between MNX1-AS1,LINC00330,LSAMP-AS1 knockdown group and non-specific control group; (**D**) Comparison ability of colony formation in TU177 cells between MNX1-AS1, LINC00330, LSAMP-AS1 knockdown group and non-specific control group; (**E**) Comparison ability of colony formation in AMC-HN-8 cells between MNX1-AS1, LINC00330, LSAMP-AS1 knockdown group and non-specific control group; (**F**) Comparison ability of migration and invasion in TU177 cells between MNX1-AS1, LINC00330, LSAMP-AS1 knockdown group and non-specific control group; (**G**) Comparison ability of migration and invasion in AMC-HN-8 cells between MNX1-AS1, LINC00330, LSAMP-AS1 knockdown group and NC group; (**H**) Comparison ability of cell migration in TU177 cells between MNX1-AS1,LINC00330,LSAMP-AS1 knockdown group and non-specific control group; (**I**) Comparison ability of cell migration in AMC-HN-8 cells between MNX1-AS1, LINC00330, LSAMP-AS1 knockdown group and non-specific control group.

After transfection with *MNX1-AS1*, *LINC00330*, and* LSAMP-AS1* overexpression plasmids, the cell viability of the *MNX1-AS1, LINC00330*, *LSAMP-AS1* TU177, and AMC-HN-8 overexpression groups was higher than that of the control group (P < 0.05) (Fig. 7A,B), which was more obvious at 48 h and 72 h. In the migration and invasion assays, the number of penetrating cells in the *MNX1-AS1, LINC00330, and LSAMP-AS1* overexpression groups was greater than that in the control group (*P* < 0.01) (Fig. 7C,D). The number of clones formed in the overexpression group was significantly higher than that in the control group (*P* < 0.01) (Fig. 7E,F). The cell migration ratio in the overexpression group was significantly higher than that in the control group (*P* < 0.01) (Fig. 7G,H). In brief, it can be concluded that overexpression of *MNX1-AS1*, *LINC00330*, and* LSAMP-AS1* can increase the proliferation, cell viability, migration, and invasion of TU177 and AMC-HN-8 cells and vice versa (Fig. 8).

### Western blot

To further prove the relationship between *MNX1-AS1*, *LINC00330*, *LSAMP-AS1* and the *PI3K/Akt* pathway. Compared with the control group, *p-AKT* (Ser473) and P-PI3K (Tyr467/Tyr199) in the three groups overexpressing *MNX1-AS1*, *LINC00330* and *LSAMP-AS1* were significantly activated. There was no significant difference in AKT total protein and PI3K total protein between the three overexpression groups and the control group. (Fig. 7J,I). It is known that the *PI3K/Akt* pathway is significantly activated in laryngeal cancer cells after overexpression of *MNX1-AS1*, *LINC00330*, and* LSAMP-AS1*.

## Discussion

In accordance with the above study, we established a prognostic signature related to the *PI3K/Akt* signalling pathway through the TCGA database and achieved good results. The predictive effect of the nomogram was better than that of clinicopathological criteria (AUC: 0.819 vs. 0.381). The external verification set of clinical samples also showed good consistency with the establishment of the model.

It is generally known that the *PI3K/Akt* signalling pathway plays a role in a variety of tumour phenotypes (e.g., angiogenesis, migration and invasion, growth and proliferation, phagocytosis, apoptosis, and metabolic reprogramming)^2^. *p-AKT* is necessary for tumour survival and spread. Many studies have confirmed the existence of *AKT* activation in critical cancers affecting human survival, such as non-small cell lung cancer (NSCLC), gastric cancer, and head and neck cancer, and laryngeal cancer is no exception^20–24^. There is a strong correlation between *AKT* activation and low differentiation, high malignant degree, metastasis, and other malignant behaviours^1^. Of course, the activation of *AKT* is closely related to the decreased survival rate of tumour patients. This view has been confirmed by reports^20^. *AKT* is activated in laryngeal papilloma, which has been recognized by otorhinolaryngologists as a laryngeal precancerous lesion^25^. In brief, the activation of the *PI3K/Akt* signalling pathway runs through the whole process of the tumour from initiation to development.

LncRNAs play a vital role in tumour regulation. Existing research has suggested that the regulation of lncRNA comprises transcription, posttranscriptional translation, and other processes and plays an essential role^26^. For instance, lncRNAs can produce specific transcription after some signal stimulation and regulate the transcription of downstream genes. Since lncRNA does not involve translation, it also responds rapidly to body signals^27^. LncRNA can serve as a molecular bait, including lncRNA and miRNA binding that competitively blocks the effect of miRNA on mRNA^28^ or directly binding to a protein blocking the action and pathway of the molecule^29^. In brief, the screening of *PI3K/Akt* signalling pathway-associated lncRNAs and the establishment of the prognostic signature take on a critical significance in the prognosis of tumour patients. Through the log-rank test, univariate Cox analysis, and multivariate Cox analysis, we selected three lncRNAs to participate in the establishment of the signature (*MNX1-AS1*, *LINC00330*, *LSAMP-AS1*). In a study of breast cancer, *p-AKT* was significantly activated in cells expressing the *MNX1-AS1* vector, and the expression of the downstream target genes *CDK4*, *bcl2*, *c-myc* and *cyClinD1* was also significantly upregulated^30^. The pathways of *LINC00330* and *LSAMP-AS1* have not been reported.

The correlation between lncRNAs and mRNAs of the PI3K/Akt pathway was analysed. The mRNAs coexpressed with *MNX1-AS1*, *LINC00330*, and* LSAMP-AS1* were extracellular matrix factor proteins (*COL1A1*, *COL1A2*, *COL6A1*, *COL6A2, COL6A3*, and* IBSP*), growth factors (*FGF19* and* PDGFRB*), G protein-coupled receptors connexin (*GNG* and* GNG4*), *CYS2*, and* TNXB*. The extracellular matrix (ECM) is composed of enzymes, glycoproteins, collagen, etc. It is similar to the soil in which cells are conceived, providing nutrients and support for cells. During the development of tumour cells, extracellular matrix collagen adhesion, fibrosis, extracellular matrix hardening, and ECM remodelling occur, and the activity of *PI3K* can also be enhanced^31^, thus promoting local tumour adhesion and migration and invasion via the basement membrane. Existing research has also provided evidence that ECM factors can activate the* PI3K/Akt* signalling pathway^32^. For instance, IBSP siRNA significantly inhibited the levels of *AKT*, *p-AKT*, *PI3K*, and *p-PI3K* in osteoblasts. According to the KEGG pathway map, *FGF19 and PDGFRβ,* as growth factors (GFs), can activate tyrosine kinase receptor (*RTK*) and then activate *PI3K*. Existing research has also shown that FGF19 and *PDGFRβ* significantly upregulate the phosphorylation levels of *PI3K*, *AKT*, and* mTOM*^33–35^. In addition to tyrosine kinase receptor proteins, there are also G protein-coupled receptor proteins in the *PI3K/Akt* signalling pathway. Factors of the chemokine pathway can directly stimulate G-protein-coupled receptors and promote the phosphorylation of the *PI3K* pathway. *GNG* and *GNG4* are members of the G-protein-coupled receptor-binding protein family and important activators of *PI3K/Akt. TNXB* is a member of the *TGF-β* protein family, and the effective activation of the *PI3K/Akt* signalling pathway by *TGF-β* has also been confirmed^36,37^. The above results reveal that the coexpression of lncRNAs in the model mRNA plays a key role in the *PI3K/Akt* signalling pathway, and the lncRNA model based on the above functional mRNAs is positively correlated with the *PI3K/Akt* signalling pathway.

The lncRNA prognostic model has been applied to various diseases, such as the ferroptosis-related bladder cancer lncRNA prognostic signature^38^, glycolysis-related hepatocellular carcinoma lncRNA signature^39^, and autophagy-related lncRNA signature for patients with breast cancer^40^. According to the multivariate Cox regression model, we calculated risk scores for each patient and then used the median risk score to divide patients into high- and low-risk groups. The survival analysis showed that there was a significant difference in prognosis between the high- and low-risk groups, and the survival time of the low-risk group was significantly longer than that of the high-risk group (P < 0.01). The patient's risk score was inversely proportional to T follicular helper cells (Tfhs) (R = − 0.27, P = 0.037). The content of Tfh cells in the low-risk group was higher than that in the high-risk group (*P* = 0.022). In an immunological study, it was proposed that PD-1 and PD-L1 are important factors that prevent the body from overimmunity to persistent antigen stimulation, and they are also used by tumours for “immune escape”^41,42^. Researchers have found that the accumulation of many Tfh cells can promote tumour CD8+-dependent antitumour immunity while resisting the role of PD-L1, weakening the role of tumour immune escape, and increasing tumour cell death^43,44^. Tfh cells are also professional helper cells of B cells, playing a role in and maintaining the formation of germinal centres and promoting B cells to differentiate into plasma cells and memory B cells in germinal centres, which are the basic components of acquired immunity and memory immunity. The decrease in Tfh cells in the high-risk group suggests that there is tumour immunosuppression in the high-risk group. This study also found that the high-risk group was sensitive to AKT inhibitors such as AKT inhibitor VIII and A-443654, as well as to an *ERK* pathway inhibitor (BIX02189), a PDK1/AKT inhibitor (BX-912) and a tyrosine kinase inhibitor (axitinib) of the *PI3K/AKt* signalling pathway. Cytarabine also showed the superiority of treatment in the high-risk group.

## Conclusion

Our study confirmed that the signature constructed by three lncRNAs related to the PI3K/Akt pathways has good prognostic efficacy, and this was also confirmed by the clinical external validation set. In in vitro verification assays, overexpression of MNX1-AS1, LINC00330, and LSAMP-AS1 promoted the proliferation, migration, and invasion of laryngeal cancer cells, and vice versa. Western blot analysis of proteins related to the PI3K/Akt pathway confirmed that P-PI3K and P-AKT were activated after the overexpression of the three lncRNAs. In summary, this signature has a good ability to predict prognosis.

### Supplementary Information

Supplementary Figure S1.

Supplementary Figure S2.

Supplementary Table S1.

Supplementary Information 1.

Supplementary Information 2.

Supplementary Information 3.

Supplementary Information 4.

## Data Availability

The public datasets generated and analysed during the current study are available in Genomic Data Commons [TCGA-HNSC(larynx)] (GDC, https://portal.gdc.cancer.gov/repository). RNA primer sequences are available in the National Center for Biotechnology Information (NCBI, http://www.ncbi.nlm.nih.gov/) repository. In addition, the related data of 45 patients are original data, not public data. The datasets generated during and/or analysed during the current study are not publicly available due to patient privacy but are available from the corresponding author on reasonable request.
