# Supplementary material for: PI3K/Akt signalling pathway-associated long noncoding RNA signature predicts the prognosis of laryngeal cancer patients
Source: Sci Rep. 2023 Sep 7;13:14764. doi: 10.1038/s41598-023-41927-3 (PMC10485045; doi:10.1038/s41598-023-41927-3)
Supplement: Supplementary file 1 — Supplementary Figure S1. [file 41598_2023_41927_MOESM1_ESM.pdf]

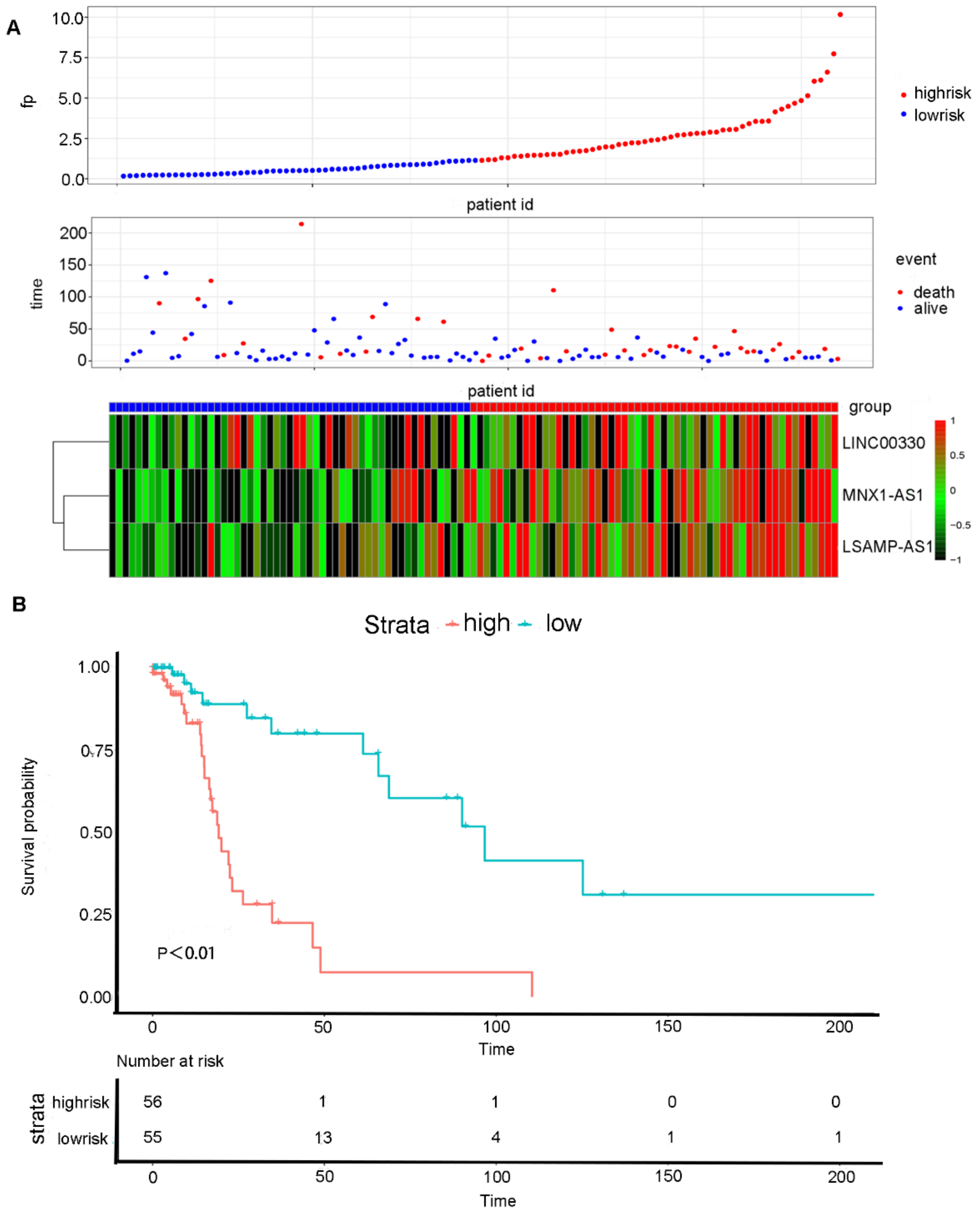

Supplementary figure S1: Relationship between risk score and prognosis of patients with laryngeal carcinoma. (A):The distribution of risk scores in patients with laryngeal cancer, the survival and death state of patients under different risk scores, and the expression of 3lncRNAs in laryngeal cancer tissues and matched normal tissues(.B): Kaplan-Meier Survival Analysis of High and low risk subgroups
