# Supplementary material for: PI3K/Akt signalling pathway-associated long noncoding RNA signature predicts the prognosis of laryngeal cancer patients
Source: Sci Rep. 2023 Sep 7;13:14764. doi: 10.1038/s41598-023-41927-3 (PMC10485045; doi:10.1038/s41598-023-41927-3)
Supplement: Supplementary file 2 — Supplementary Figure S2. [file 41598_2023_41927_MOESM2_ESM.pdf]

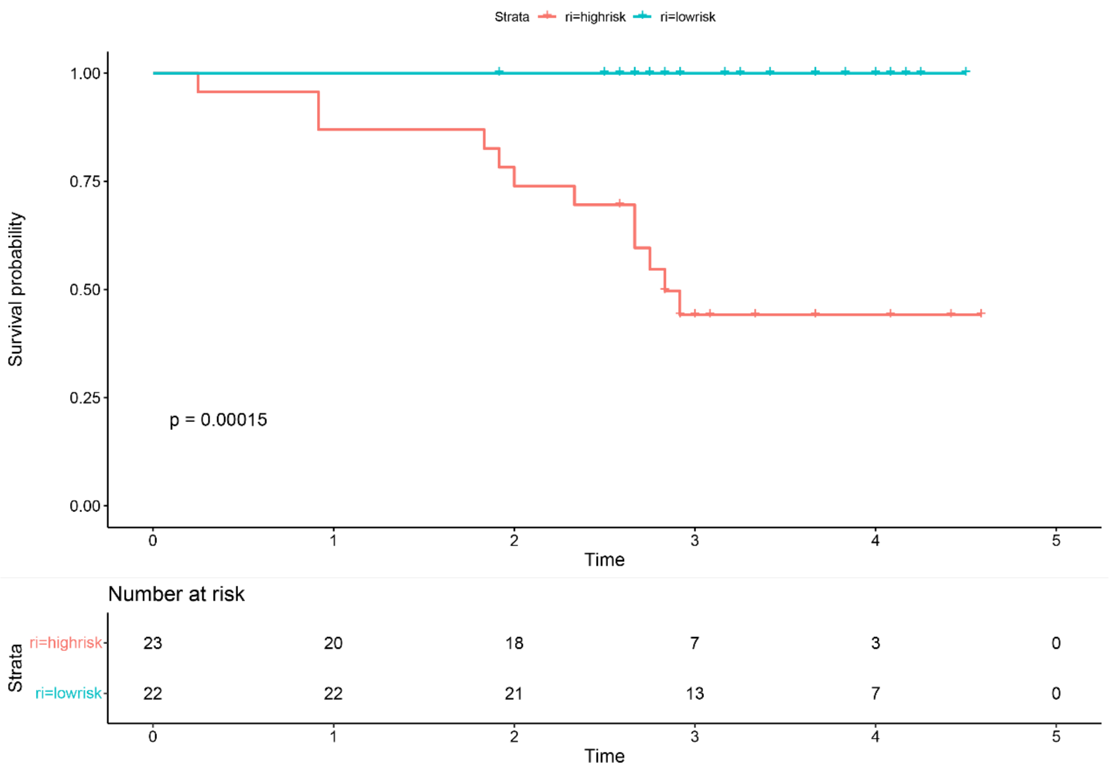

Supplementary figure S2: Kaplan-Meier Survival Analysis of High and low risk subgroups in clinical external verification set.
