# Supplementary material for: PI3K/Akt signalling pathway-associated long noncoding RNA signature predicts the prognosis of laryngeal cancer patients
Source: Sci Rep. 2023 Sep 7;13:14764. doi: 10.1038/s41598-023-41927-3 (PMC10485045; doi:10.1038/s41598-023-41927-3)
Supplement: Supplementary file 3 — Supplementary Table S1. [file 41598_2023_41927_MOESM3_ESM.docx]

Supplementary Table 1: Risk scores of patients form TCGA

| ID riskscore | |  | |
| --- | --- | --- | --- |
| TCGA-CN-6988 | 1.137036944 | |  |
| TCGA-CV-5440 | 0.327477654 | |  |
| TCGA-BA-6868 | 0.901312938 | |  |
| TCGA-CV-7250 | 0.251348139 | |  |
| TCGA-CN-5355 | 0.633485432 | |  |
| TCGA-CV-7430 | 2.15773571 | |  |
| TCGA-CN-4739 | 3.042368257 | |  |
| TCGA-CV-7418 | 4.310823453 | |  |
| TCGA-CV-A6K1 | 1.743534588 | |  |
| TCGA-CV-5441 | 0.799163431 | |  |
| TCGA-CV-5444 | 0.588700167 | |  |
| TCGA-CV-7177 | 2.888947925 | |  |
| TCGA-CV-7242 | 0.645984115 | |  |
| TCGA-CN-5356 | 3.017664598 | |  |
| TCGA-F7-A623 | 1.436640455 | |  |
| TCGA-CN-4735 | 0.327477654 | |  |
| TCGA-QK-A8Z8 | 0.528846815 | |  |
| TCGA-CN-4738 | 0.693498233 | |  |
| TCGA-CN-6989 | 1.812370825 | |  |
| TCGA-CN-A6V3 | 0.508760515 | |  |
| TCGA-CV-6962 | 1.464985406 | |  |
| TCGA-CN-6021 | 2.301206025 | |  |
| TCGA-CV-7424 | 3.552039294 | |  |
| TCGA-CV-5432 | 0.215282978 | |  |
| TCGA-TN-A7HJ | 0.915314399 | |  |
| TCGA-CN-A641 | 1.137036944 | |  |
| TCGA-CR-7389 | 2.413555095 | |  |
| TCGA-BA-A6DI | 0.59784534 | |  |
| TCGA-CV-5431 | 2.815650792 | |  |
| TCGA-CN-6023 | 2.815650792 | |  |
| TCGA-CV-A45W | 3.062113263 | |  |
| TCGA-UF-A7JK | 4.841832065 | |  |
| TCGA-CR-7398 | 6.041904245 | |  |
| TCGA-CV-A461 | 0.7444098 | |  |
| TCGA-CN-6012 | 0.23261766 | |  |
| TCGA-UF-A7J9 | 1.183765331 | |  |
| TCGA-CN-4727 | 0.280488907 | |  |
| TCGA-CR-7388 | 0.359058277 | |  |
| TCGA-QK-A8ZB | 1.493360249 | |  |
| TCGA-CV-7415 | 2.584669319 | |  |
| TCGA-UF-A71D | 0.867079883 | |  |
| TCGA-CV-7101 | 4.675291082 | |  |
| TCGA-D6-A6EQ | 0.830715086 | |  |
| TCGA-CV-5434 | 1.513004863 | |  |
| TCGA-CN-5361 | 0.975400875 | |  |
| TCGA-CR-7402 | 1.45895797 | |  |
| TCGA-BB-4217 | 0.387969811 | |  |
| TCGA-UF-A7JF | 0.243781077 | |  |
| TCGA-CV-7421 | 1.13880436 | |  |
| TCGA-CN-6010 | 6.108135923 | |  |
| TCGA-D6-A74Q | 0.183203701 | |  |
| TCGA-CV-6935 | 1.971013151 | |  |
| TCGA-CN-6992 | 4.481018449 | |  |
| TCGA-CR-7364 | 0.512062377 | |  |
| TCGA-CR-6474 | 6.599965767 | |  |
| TCGA-CN-A63T | 0.240242428 | |  |
| TCGA-CN-A497 | 0.611942553 | |  |
| TCGA-UF-A7JH | 0.768810148 | |  |
| TCGA-CN-4722 | 1.107793285 | |  |
| TCGA-CV-7440 | 2.699218956 | |  |
| TCGA-BA-5555 | 1.908707124 | |  |
| TCGA-UF-A718 | 0.222339548 | |  |
| TCGA-KU-A66S | 1.304318977 | |  |
| TCGA-CV-A460 | 1.023021723 | |  |
| TCGA-CV-7433 | 3.239623113 | |  |
| TCGA-F7-A50I | 0.46473424 | |  |
| TCGA-CN-A49B | 0.191672677 | |  |
| TCGA-CV-A45Z | 1.983264783 | |  |
| TCGA-UF-A7JJ | 0.485078118 | |  |
| TCGA-CV-A45Y | 0.231117704 | |  |
| TCGA-D6-6517 | 0.872707241 | |  |
| TCGA-CV-7089 | 0.879851528 | |  |
| TCGA-CV-5430 | 0.231117704 | |  |
| TCGA-QK-AA3J | 5.142764325 | |  |
| TCGA-CN-A63W | 1.09385078 | |  |
| TCGA-D6-8568 | 0.48413006 | |  |
| TCGA-CV-7261 | 2.233696825 | |  |
| TCGA-CV-5978 | 2.774779699 | |  |
| TCGA-CV-7248 | 4.147092686 | |  |
| TCGA-CV-5443 | 0.261271578 | |  |
| TCGA-CN-A63U | 2.722057028 | |  |
| TCGA-CV-7247 | 1.397518928 | |  |
| TCGA-CN-6997 | 1.677312843 | |  |
| TCGA-CN-5360 | 0.542588584 | |  |
| TCGA-BA-6870 | 1.62810645 | |  |
| TCGA-D6-6824 | 0.489437119 | |  |
| TCGA-CV-7245 | 0.836106437 | |  |
| TCGA-CR-7374 | 7.734337 | |  |
| TCGA-HD-7229 | 1.513004863 | |  |
| TCGA-DQ-5629 | 1.394494534 | |  |
| TCGA-T3-A92M | 2.884330375 | |  |
| TCGA-F7-7848 | 0.397433202 | |  |
| TCGA-D6-A6EK | 0.403287191 | |  |
| TCGA-CN-4723 | 3.552039294 | |  |
| TCGA-CV-5435 | 0.269835568 | |  |
| TCGA-D6-6826 | 1.295908515 | |  |
| TCGA-CV-7410 | 0.503243928 | |  |
| TCGA-CV-7437 | 2.385491954 | |  |
| TCGA-F7-A622 | 0.164170162 | |  |
| TCGA-BA-4078 | 0.303545099 | |  |
| TCGA-BA-4076 | 3.411824793 | |  |
| TCGA-BA-6869 | 1.710406265 | |  |
| TCGA-F7-8298 | 3.57682658 | |  |
| TCGA-CR-7371 | 10.17027271 | |  |
| TCGA-CR-7399 | 2.115987232 | |  |
| TCGA-CV-7422 | 0.240242428 | |  |
| TCGA-CN-6022 | 2.492666877 | |  |
| TCGA-CN-5363 | 1.177065291 | |  |
| TCGA-CR-7370 | 2.227563973 | |  |
| TCGA-BA-A6DA | 0.501375509 | |  |
| TCGA-D6-A6ES | 1.089350315 | |  |
