# Supplementary material for: PI3K/Akt signalling pathway-associated long noncoding RNA signature predicts the prognosis of laryngeal cancer patients
Source: Sci Rep. 2023 Sep 7;13:14764. doi: 10.1038/s41598-023-41927-3 (PMC10485045; doi:10.1038/s41598-023-41927-3)
Supplement: Supplementary file 4 — Supplementary Information 1. [file 41598_2023_41927_MOESM4_ESM.pdf]

P-PI3K TU177

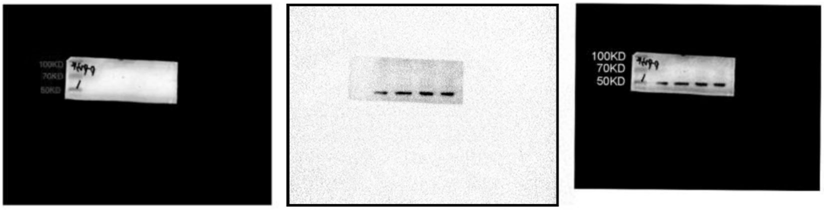

PI3K TU177

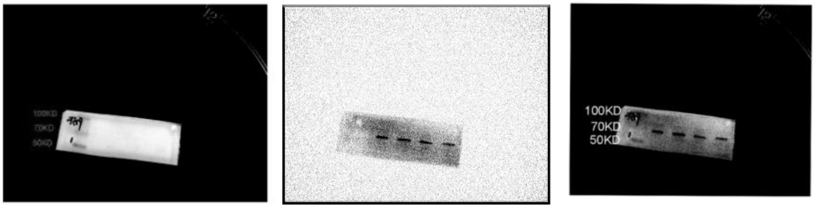

GAPDH TU177

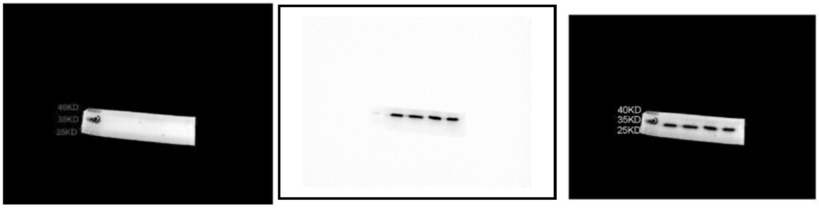

P-AKT TU177

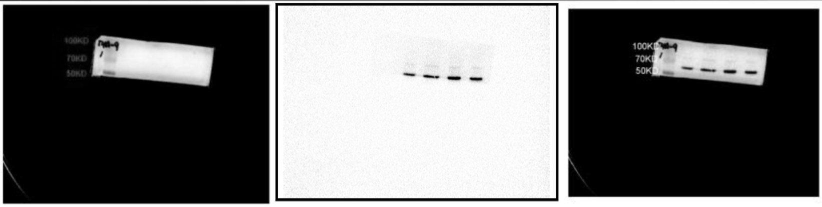

AKT TU177

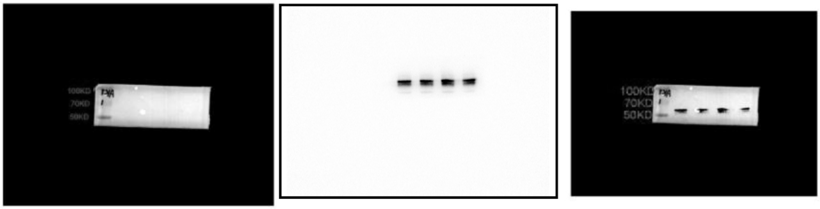

GAPDH TU177 2.0

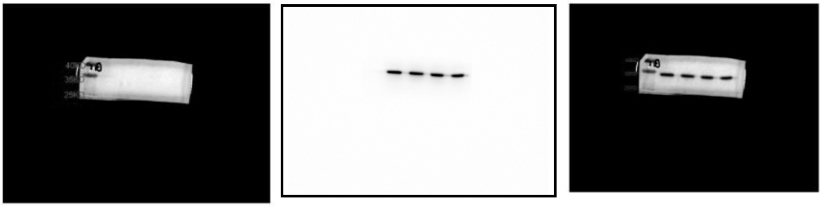

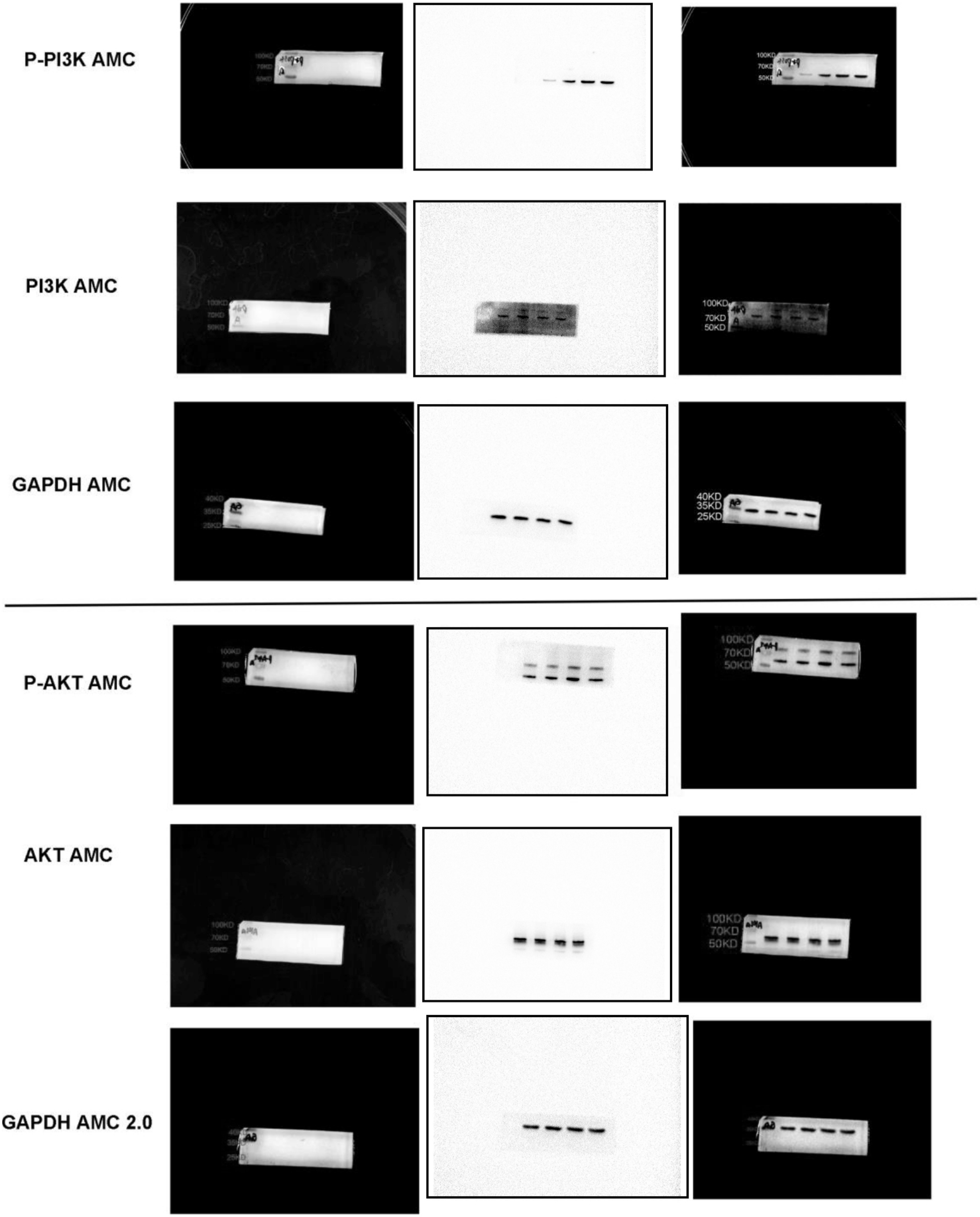

The black dividing line separates two different pieces of glue
