# Supplementary material for: PI3K/Akt signalling pathway-associated long noncoding RNA signature predicts the prognosis of laryngeal cancer patients
Source: Sci Rep. 2023 Sep 7;13:14764. doi: 10.1038/s41598-023-41927-3 (PMC10485045; doi:10.1038/s41598-023-41927-3)
Supplement: Supplementary file 5 — Supplementary Information 2. [file 41598_2023_41927_MOESM5_ESM.docx]

|  | logFC | AveExpr | t | P.Value | adj.P.Val | B | change |
| --- | --- | --- | --- | --- | --- | --- | --- |
| DLG2 | -3.84866 | -0.43172 | -17.3835 | 3.41E-35 | 6.26E-31 | 68.7425 | DOWN |
| FAM107A | -3.5211 | 1.646546 | -14.4147 | 2.24E-28 | 1.28E-24 | 53.9146 | DOWN |
| CRISP3 | -8.22582 | 0.158818 | -14.4013 | 2.41E-28 | 1.28E-24 | 53.84668 | DOWN |
| CAPN5 | -3.59838 | 3.758242 | -14.3745 | 2.78E-28 | 1.28E-24 | 53.75652 | DOWN |
| SCIN | -4.33469 | 2.202933 | -14.2813 | 4.63E-28 | 1.69E-24 | 53.23707 | DOWN |
| MYZAP | -4.31431 | -0.17459 | -14.249 | 5.53E-28 | 1.69E-24 | 52.86354 | DOWN |
| KAT2B | -2.46746 | 3.765366 | -13.8955 | 3.84E-27 | 1.00E-23 | 51.16231 | DOWN |
| SORBS2 | -3.596 | 2.652222 | -13.7126 | 1.05E-26 | 2.41E-23 | 50.16102 | DOWN |
| ATP6V0A4 | -4.97521 | 0.510675 | -13.6771 | 1.28E-26 | 2.60E-23 | 49.92271 | DOWN |
| CAB39L | -2.34912 | 2.641334 | -13.4164 | 5.39E-26 | 9.88E-23 | 48.53118 | DOWN |
| SH3BGRL2 | -3.9717 | 3.831694 | -13.3399 | 8.24E-26 | 1.37E-22 | 48.1306 | DOWN |
| HPGD | -4.53091 | 2.798798 | -12.9532 | 7.06E-25 | 1.05E-21 | 46.00617 | DOWN |
| MYOC | -6.62816 | -3.94225 | -12.9379 | 7.69E-25 | 1.05E-21 | 45.46714 | DOWN |
| C15orf62 | -3.54205 | 2.19841 | -12.8152 | 1.52E-24 | 1.64E-21 | 45.24243 | DOWN |
| PADI1 | -6.10394 | 2.343067 | -12.7797 | 1.86E-24 | 1.89E-21 | 45.04971 | DOWN |
| NRG2 | -3.90107 | -1.62377 | -12.817 | 1.51E-24 | 1.64E-21 | 44.77941 | DOWN |
| GPD1L | -2.63361 | 4.212121 | -12.626 | 4.38E-24 | 4.02E-21 | 44.19743 | DOWN |
| KIAA1211L | -3.00968 | 2.688174 | -12.4575 | 1.13E-23 | 8.97E-21 | 43.27086 | DOWN |
| GYS2 | -5.36061 | -3.98683 | -12.4973 | 9.01E-24 | 7.51E-21 | 42.80118 | DOWN |
| MUC21 | -8.51293 | 1.437262 | -12.3257 | 2.36E-23 | 1.67E-20 | 42.54016 | DOWN |
| PLEKHA6 | -3.21803 | 2.685765 | -12.3194 | 2.44E-23 | 1.67E-20 | 42.50658 | DOWN |
| FMO2 | -5.36524 | 3.00326 | -12.318 | 2.46E-23 | 1.67E-20 | 42.49727 | DOWN |
| SH2D1B | -4.58224 | -1.05448 | -12.3059 | 2.63E-23 | 1.72E-20 | 42.26368 | DOWN |
| VSIG2 | -4.62567 | 0.976268 | -12.2723 | 3.18E-23 | 2.01E-20 | 42.23949 | DOWN |
| GNA14 | -2.90104 | 0.171454 | -12.2465 | 3.67E-23 | 2.24E-20 | 41.92026 | DOWN |
| CYP4B1 | -5.76457 | 1.07626 | -12.1042 | 8.17E-23 | 4.68E-20 | 41.31434 | DOWN |
| COL4A2 | 3.516317 | 8.588683 | 11.94832 | 1.96E-22 | 1.09E-19 | 40.43963 | UP |
| MUC22 | -7.48489 | -1.66195 | -11.8167 | 4.11E-22 | 2.15E-19 | 39.70915 | DOWN |
| CXCR2 | -3.84923 | 1.286034 | -11.7877 | 4.84E-22 | 2.47E-19 | 39.55654 | DOWN |
| SNX31 | -4.76666 | 0.509523 | -11.7426 | 6.24E-22 | 3.01E-19 | 39.30353 | DOWN |
| TJP3 | -4.20274 | 2.75254 | -11.7342 | 6.54E-22 | 3.08E-19 | 39.25575 | DOWN |
| ANXA9 | -3.60925 | 2.461361 | -11.7264 | 6.84E-22 | 3.13E-19 | 39.21634 | DOWN |
| CLCA4 | -6.7081 | 3.527206 | -11.7156 | 7.27E-22 | 3.25E-19 | 39.15149 | DOWN |
| EDN3 | -6.14191 | -3.83393 | -11.7757 | 5.18E-22 | 2.57E-19 | 39.1331 | DOWN |
| COL4A1 | 3.720793 | 8.275852 | 11.43814 | 3.47E-21 | 1.51E-18 | 37.60727 | UP |
| SMIM5 | -3.56289 | 1.653968 | -11.4155 | 3.94E-21 | 1.64E-18 | 37.49327 | DOWN |
| MAL | -7.00612 | 3.247895 | -11.3007 | 7.53E-21 | 2.94E-18 | 36.84295 | DOWN |
| TRNP1 | -3.88453 | 3.630746 | -11.2598 | 9.49E-21 | 3.62E-18 | 36.60335 | DOWN |
| PDE6A | -3.58594 | -1.8596 | -11.3068 | 7.28E-21 | 2.90E-18 | 36.53453 | DOWN |
| ADIPOQ | -6.17334 | -4.85515 | -11.3073 | 7.26E-21 | 2.90E-18 | 36.4152 | DOWN |
| CD200R1 | -2.92081 | 0.755196 | -11.2154 | 1.22E-20 | 4.48E-18 | 36.34068 | DOWN |
| SLC27A6 | -4.26062 | -1.67211 | -11.0853 | 2.54E-20 | 8.95E-18 | 35.46783 | DOWN |
| KALRN | -2.43102 | 3.367033 | -11.0333 | 3.40E-20 | 1.18E-17 | 35.35107 | DOWN |
| KRT17 | 3.486305 | 11.98186 | 10.98236 | 4.54E-20 | 1.54E-17 | 35.04606 | UP |
| CAPN14 | -6.22025 | 2.03296 | -10.9369 | 5.86E-20 | 1.95E-17 | 34.82171 | DOWN |
| FAM3D | -5.57324 | 2.935038 | -10.911 | 6.78E-20 | 2.22E-17 | 34.66826 | DOWN |
| BCAS1 | -4.21004 | 2.22325 | -10.8488 | 9.64E-20 | 3.10E-17 | 34.33062 | DOWN |
| PDE4C | -3.36628 | 0.248376 | -10.8432 | 9.95E-20 | 3.14E-17 | 34.27904 | DOWN |
| TMPRSS11B | -7.51223 | 0.793037 | -10.8112 | 1.19E-19 | 3.70E-17 | 34.13222 | DOWN |
| C2orf54 | -4.6325 | 3.413969 | -10.7949 | 1.31E-19 | 3.99E-17 | 34.01335 | DOWN |
| FAM3B | -4.85857 | 2.024967 | -10.7468 | 1.71E-19 | 5.15E-17 | 33.76345 | DOWN |
| PLAC8 | -4.22197 | 3.216368 | -10.5868 | 4.22E-19 | 1.23E-16 | 32.85597 | DOWN |
| CYP2F1 | -5.76232 | -2.56034 | -10.5608 | 4.89E-19 | 1.40E-16 | 32.65631 | DOWN |
| ADH1B | -5.40496 | -2.17933 | -10.5125 | 6.42E-19 | 1.78E-16 | 32.40826 | DOWN |
| GPT | -2.80295 | 0.937003 | -10.4488 | 9.19E-19 | 2.51E-16 | 32.11941 | DOWN |
| DPCR1 | -5.53026 | -3.59342 | -10.442 | 9.55E-19 | 2.57E-16 | 31.85704 | DOWN |
| FUT6 | -4.90455 | 2.053721 | -10.3311 | 1.78E-18 | 4.74E-16 | 31.45069 | DOWN |
| CEACAM7 | -5.90469 | 1.065839 | -10.2935 | 2.20E-18 | 5.77E-16 | 31.25415 | DOWN |
| CLEC3B | -2.88674 | 1.244958 | -10.2758 | 2.43E-18 | 6.20E-16 | 31.17379 | DOWN |
| IKZF2 | -2.82508 | 3.93372 | -10.249 | 2.83E-18 | 7.11E-16 | 30.96534 | DOWN |
| CRYM | -3.61455 | 0.287547 | -10.214 | 3.45E-18 | 8.42E-16 | 30.82584 | DOWN |
| GPD1 | -3.41634 | -0.2461 | -10.2057 | 3.61E-18 | 8.71E-16 | 30.74575 | DOWN |
| CGNL1 | -2.81363 | 2.771967 | -10.1705 | 4.40E-18 | 1.05E-15 | 30.55966 | DOWN |
| GDPD3 | -3.48588 | 2.422473 | -10.1284 | 5.58E-18 | 1.28E-15 | 30.32848 | DOWN |
| PSCA | -5.28559 | 3.056135 | -10.1313 | 5.49E-18 | 1.27E-15 | 30.32588 | DOWN |
| HTR3B | -5.59545 | -3.95642 | -10.164 | 4.57E-18 | 1.07E-15 | 30.31142 | DOWN |
| AC011513.3 | -2.931 | -5.95343 | -10.2275 | 3.19E-18 | 7.91E-16 | 30.2789 | DOWN |
| CYP2J2 | -2.5637 | 1.757106 | -10.0705 | 7.72E-18 | 1.71E-15 | 30.0395 | DOWN |
| PPP1R3C | -3.68311 | 2.679809 | -10.0268 | 9.87E-18 | 2.13E-15 | 29.75485 | DOWN |
| HOXD11 | 7.190438 | 1.137848 | 10.10804 | 6.25E-18 | 1.42E-15 | 29.69944 | UP |
| COBL | -3.48437 | 2.44892 | -10.001 | 1.14E-17 | 2.43E-15 | 29.61964 | DOWN |
| PHYHIP | -2.94743 | 1.357724 | -9.95619 | 1.47E-17 | 3.02E-15 | 29.41088 | DOWN |
| TMEM132C | -4.69943 | -3.30093 | -9.99632 | 1.17E-17 | 2.47E-15 | 29.39298 | DOWN |
| DYNAP | -6.07248 | -2.65623 | -9.88378 | 2.20E-17 | 4.34E-15 | 28.97832 | DOWN |
| ADH4 | -4.56548 | -4.2435 | -9.9387 | 1.62E-17 | 3.30E-15 | 28.93771 | DOWN |
| KLHDC7A | -5.066 | -1.75606 | -9.84348 | 2.76E-17 | 5.33E-15 | 28.75521 | DOWN |
| SPNS2 | -3.4989 | 4.523753 | -9.78612 | 3.81E-17 | 7.27E-15 | 28.38717 | DOWN |
| ARSF | -5.23156 | -3.1718 | -9.7076 | 5.91E-17 | 1.12E-14 | 27.91104 | DOWN |
| FAM149A | -2.70108 | 0.475381 | -9.66723 | 7.41E-17 | 1.36E-14 | 27.81126 | DOWN |
| C16orf89 | -3.83053 | -1.83888 | -9.67804 | 6.98E-17 | 1.29E-14 | 27.76191 | DOWN |
| MKRN2OS | -3.01766 | -0.39221 | -9.66199 | 7.63E-17 | 1.39E-14 | 27.74153 | DOWN |
| BCL2L15 | -4.07146 | -1.35541 | -9.64042 | 8.61E-17 | 1.55E-14 | 27.61964 | DOWN |
| CRNN | -8.21319 | 2.823628 | -9.62597 | 9.34E-17 | 1.65E-14 | 27.54825 | DOWN |
| NR3C2 | -2.79586 | 0.514709 | -9.60865 | 1.03E-16 | 1.76E-14 | 27.49705 | DOWN |
| FAM189A2 | -3.08701 | 0.831197 | -9.58488 | 1.17E-16 | 1.98E-14 | 27.37446 | DOWN |
| ASPA | -3.32583 | -2.33056 | -9.60937 | 1.02E-16 | 1.76E-14 | 27.28605 | DOWN |
| ZIC5 | 6.528046 | 0.908411 | 9.617326 | 9.80E-17 | 1.71E-14 | 27.1013 | UP |
| CH25H | -2.62137 | 2.130686 | -9.538 | 1.53E-16 | 2.54E-14 | 27.09345 | DOWN |
| SLC46A2 | -3.90637 | -2.7928 | -9.51409 | 1.74E-16 | 2.88E-14 | 26.78128 | DOWN |
| ATP13A4 | -4.05265 | 2.539131 | -9.42161 | 2.92E-16 | 4.62E-14 | 26.41224 | DOWN |
| GCOM1 | -4.3744 | -1.56599 | -9.41385 | 3.05E-16 | 4.77E-14 | 26.3972 | DOWN |
| CDH26 | -3.4186 | 2.41527 | -9.41277 | 3.07E-16 | 4.77E-14 | 26.37565 | DOWN |
| UPK1A | -5.01791 | -1.18558 | -9.39018 | 3.48E-16 | 5.32E-14 | 26.30868 | DOWN |
| ENDOU | -4.76431 | 0.978425 | -9.39206 | 3.45E-16 | 5.31E-14 | 26.28876 | DOWN |
| MC5R | -3.96934 | -5.07265 | -9.44872 | 2.51E-16 | 4.11E-14 | 26.19712 | DOWN |
| GALNT12 | -2.35819 | 3.350783 | -9.32705 | 4.95E-16 | 7.38E-14 | 25.88781 | DOWN |
| GPR110 | -4.60401 | 3.009211 | -9.2993 | 5.78E-16 | 8.47E-14 | 25.72743 | DOWN |
| SCGB1A1 | -5.49096 | -1.36449 | -9.27624 | 6.57E-16 | 9.41E-14 | 25.69106 | DOWN |
| C9orf152 | -4.35967 | -0.61338 | -9.27605 | 6.57E-16 | 9.41E-14 | 25.68873 | DOWN |
| RORC | -3.23446 | 1.037187 | -9.27709 | 6.53E-16 | 9.41E-14 | 25.6885 | DOWN |
| TUSC5 | -4.38805 | -4.82355 | -9.32278 | 5.07E-16 | 7.49E-14 | 25.58732 | DOWN |
| COL1A1 | 4.310892 | 10.99813 | 9.270341 | 6.78E-16 | 9.64E-14 | 25.53204 | UP |
| SASH1 | -2.86737 | 4.925118 | -9.26228 | 7.10E-16 | 1.00E-13 | 25.49219 | DOWN |
| EMP1 | -3.15989 | 8.416279 | -9.21419 | 9.27E-16 | 1.29E-13 | 25.25086 | DOWN |
| IL36A | -5.84541 | 0.458274 | -9.1852 | 1.09E-15 | 1.49E-13 | 25.1556 | DOWN |
| CEACAM1 | -3.67222 | 4.366468 | -9.19875 | 1.01E-15 | 1.39E-13 | 25.15079 | DOWN |
| FCER1A | -3.1467 | 0.661616 | -9.16765 | 1.20E-15 | 1.62E-13 | 25.1012 | DOWN |
| GABRP | -5.60297 | 3.282889 | -9.18134 | 1.11E-15 | 1.51E-13 | 25.07986 | DOWN |
| SERPINB11 | -5.07306 | 0.547864 | -9.1431 | 1.38E-15 | 1.83E-13 | 24.93428 | DOWN |
| CYP4F12 | -3.54457 | 0.936949 | -9.13793 | 1.42E-15 | 1.87E-13 | 24.92659 | DOWN |
| GREM2 | -3.60022 | -1.03666 | -9.13576 | 1.43E-15 | 1.88E-13 | 24.8926 | DOWN |
| KIAA1456 | -3.07588 | -0.2294 | -9.09404 | 1.81E-15 | 2.30E-13 | 24.68713 | DOWN |
| SCNN1B | -3.55996 | 3.491045 | -9.08775 | 1.87E-15 | 2.36E-13 | 24.55834 | DOWN |
| OTC | -2.78388 | -5.95054 | -9.12766 | 1.50E-15 | 1.93E-13 | 24.4967 | DOWN |
| C1orf177 | -3.37506 | -0.3342 | -9.01166 | 2.85E-15 | 3.50E-13 | 24.24835 | DOWN |
| CYP11A1 | -4.25337 | -1.25286 | -9.00424 | 2.97E-15 | 3.60E-13 | 24.20266 | DOWN |
| LMO7 | -2.60619 | 6.491479 | -9.02045 | 2.71E-15 | 3.38E-13 | 24.16691 | DOWN |
| SELENBP1 | -2.5081 | 3.172412 | -8.97351 | 3.52E-15 | 4.22E-13 | 23.95782 | DOWN |
| RBM20 | -2.91613 | -0.06377 | -8.94012 | 4.23E-15 | 5.04E-13 | 23.86159 | DOWN |
| LGI1 | -4.07887 | -4.83634 | -8.99042 | 3.20E-15 | 3.86E-13 | 23.818 | DOWN |
| B3GNT6 | -5.42049 | -1.5994 | -8.86285 | 6.48E-15 | 7.29E-13 | 23.45545 | DOWN |
| KRT40 | -4.99508 | -3.64871 | -8.85464 | 6.78E-15 | 7.58E-13 | 23.28981 | DOWN |
| ANGPTL1 | -3.39199 | -1.09154 | -8.82437 | 8.01E-15 | 8.90E-13 | 23.2132 | DOWN |
| ADAMTS20 | 6.135124 | -1.00226 | 8.866952 | 6.34E-15 | 7.17E-13 | 23.15878 | UP |
| SLC16A7 | -2.62064 | 2.991097 | -8.81742 | 8.33E-15 | 9.19E-13 | 23.1141 | DOWN |
| PLIN1 | -3.86439 | -2.38431 | -8.75866 | 1.15E-14 | 1.23E-12 | 22.80785 | DOWN |
| BLNK | -2.47781 | 3.587871 | -8.73001 | 1.35E-14 | 1.41E-12 | 22.61773 | DOWN |
| SLC6A1 | -3.23576 | -0.90007 | -8.70875 | 1.51E-14 | 1.56E-12 | 22.60035 | DOWN |
| GCNT3 | -3.94197 | 3.174751 | -8.71692 | 1.45E-14 | 1.50E-12 | 22.54606 | DOWN |
| KRT32 | -3.7078 | -1.63405 | -8.6571 | 2.01E-14 | 1.99E-12 | 22.30836 | DOWN |
| AQP5 | -5.40419 | 1.143483 | -8.66258 | 1.95E-14 | 1.95E-12 | 22.30227 | DOWN |
| PAX1 | -4.92636 | -1.5808 | -8.63854 | 2.23E-14 | 2.17E-12 | 22.25048 | DOWN |
| LYPD2 | -5.23691 | 1.967679 | -8.63799 | 2.23E-14 | 2.17E-12 | 22.14831 | DOWN |
| ALDH1L1 | -3.38524 | 1.751704 | -8.6132 | 2.56E-14 | 2.45E-12 | 22.05026 | DOWN |
| SPINK8 | -4.68517 | -4.80956 | -8.62103 | 2.45E-14 | 2.36E-12 | 21.94105 | DOWN |
| SCN7A | -3.72891 | -2.82186 | -8.59572 | 2.81E-14 | 2.69E-12 | 21.90492 | DOWN |
| RNF224 | -3.70391 | -1.76352 | -8.56002 | 3.42E-14 | 3.22E-12 | 21.78828 | DOWN |
| SLC16A6 | -2.41035 | 1.980394 | -8.52146 | 4.23E-14 | 3.91E-12 | 21.58338 | DOWN |
| TRPC4 | 3.019285 | -0.98497 | 8.532256 | 3.98E-14 | 3.71E-12 | 21.41259 | UP |
| SLC7A4 | -3.57608 | 0.347568 | -8.47433 | 5.47E-14 | 4.96E-12 | 21.36694 | DOWN |
| HS3ST1 | -2.48139 | 3.464763 | -8.49753 | 4.82E-14 | 4.39E-12 | 21.36596 | DOWN |
| ENPP4 | -2.38618 | 1.90607 | -8.46969 | 5.61E-14 | 5.06E-12 | 21.30869 | DOWN |
| MAPT | -2.95161 | 0.85744 | -8.45563 | 6.05E-14 | 5.36E-12 | 21.26595 | DOWN |
| ESM1 | 4.77883 | 1.852626 | 8.466457 | 5.71E-14 | 5.13E-12 | 21.10964 | UP |
| RAET1E | -3.61762 | 2.28893 | -8.42338 | 7.22E-14 | 6.30E-12 | 20.9997 | DOWN |
| BTC | -3.20867 | 0.064309 | -8.39691 | 8.34E-14 | 7.18E-12 | 20.967 | DOWN |
| AIF1L | -2.5753 | 4.458542 | -8.42883 | 7.01E-14 | 6.15E-12 | 20.96473 | DOWN |
| ANKRD35 | -2.86548 | 3.180477 | -8.38525 | 8.88E-14 | 7.61E-12 | 20.76973 | DOWN |
| CLCN1 | -3.03999 | -1.16072 | -8.36164 | 1.01E-13 | 8.46E-12 | 20.74674 | DOWN |
| ACPP | -2.56219 | 3.133157 | -8.3767 | 9.31E-14 | 7.94E-12 | 20.73139 | DOWN |
| SCARA5 | -3.293 | 1.192552 | -8.35108 | 1.07E-13 | 8.92E-12 | 20.67931 | DOWN |
| TP53INP2 | -2.44603 | 5.851883 | -8.37075 | 9.61E-14 | 8.08E-12 | 20.64158 | DOWN |
| CCDC60 | -4.67526 | -1.85734 | -8.33462 | 1.17E-13 | 9.71E-12 | 20.6323 | DOWN |
| CYP2E1 | -3.62723 | 0.93387 | -8.30232 | 1.39E-13 | 1.14E-11 | 20.42388 | DOWN |
| CCDC178 | -3.14171 | -3.6275 | -8.30793 | 1.35E-13 | 1.11E-11 | 20.30592 | DOWN |
| LRMP | -2.85193 | 2.346304 | -8.28186 | 1.56E-13 | 1.24E-11 | 20.258 | DOWN |
| SEC14L5 | -2.37736 | -0.3183 | -8.23066 | 2.06E-13 | 1.61E-11 | 20.07386 | DOWN |
| SLC6A4 | -3.77305 | -0.84047 | -8.21688 | 2.22E-13 | 1.71E-11 | 20.01618 | DOWN |
| ATP12A | -4.85353 | 2.784376 | -8.22452 | 2.13E-13 | 1.66E-11 | 19.90755 | DOWN |
| KRT78 | -5.65276 | 3.875308 | -8.16185 | 2.99E-13 | 2.25E-11 | 19.55766 | DOWN |
| PLA2G2A | -3.67465 | 1.301562 | -8.1409 | 3.34E-13 | 2.48E-11 | 19.54123 | DOWN |
| SLC38A3 | -3.251 | -1.83932 | -8.10593 | 4.04E-13 | 2.93E-11 | 19.39001 | DOWN |
| IL33 | -2.99078 | 2.486132 | -8.1165 | 3.81E-13 | 2.78E-11 | 19.3656 | DOWN |
| CCDC64B | -2.70328 | 4.493304 | -8.12525 | 3.64E-13 | 2.68E-11 | 19.33977 | DOWN |
| CYP3A5 | -2.82876 | 1.711591 | -8.07108 | 4.87E-13 | 3.50E-11 | 19.18428 | DOWN |
| GDF7 | -2.73183 | -0.88213 | -8.05772 | 5.24E-13 | 3.74E-11 | 19.16228 | DOWN |
| GNGT1 | 4.021018 | 0.823324 | 8.03904 | 5.79E-13 | 4.11E-11 | 18.90753 | UP |
| C2orf40 | -3.70439 | -2.02499 | -8.01055 | 6.75E-13 | 4.76E-11 | 18.90739 | DOWN |
| PLIN4 | -2.83604 | 1.410514 | -7.92528 | 1.07E-12 | 7.14E-11 | 18.43392 | DOWN |
| SPAG17 | -2.89531 | 1.967986 | -7.91623 | 1.12E-12 | 7.41E-11 | 18.34637 | DOWN |
| HOXC9 | 4.397004 | -0.0507 | 7.929568 | 1.04E-12 | 7.00E-11 | 18.34037 | UP |
| CHST9 | -4.79217 | -0.03579 | -7.89631 | 1.25E-12 | 8.10E-11 | 18.2836 | DOWN |
| NDRG2 | -2.72284 | 6.181936 | -7.92083 | 1.09E-12 | 7.26E-11 | 18.24903 | DOWN |
| PRSS27 | -4.28516 | 3.996116 | -7.91239 | 1.14E-12 | 7.48E-11 | 18.22289 | DOWN |
| KRTAP3-2 | -2.69393 | -5.9147 | -7.89763 | 1.24E-12 | 8.07E-11 | 18.16717 | DOWN |
| PERM1 | -2.39016 | 2.275163 | -7.86403 | 1.48E-12 | 9.52E-11 | 18.07898 | DOWN |
| PPL | -2.72305 | 8.72776 | -7.866 | 1.47E-12 | 9.46E-11 | 17.98861 | DOWN |
| HOXC6 | 4.185659 | 0.540431 | 7.856272 | 1.54E-12 | 9.89E-11 | 17.97709 | UP |
| TREH | -3.52856 | -3.47437 | -7.84419 | 1.65E-12 | 1.04E-10 | 17.96872 | DOWN |
| FOXS1 | 2.896272 | 0.287606 | 7.845323 | 1.64E-12 | 1.04E-10 | 17.92239 | UP |
| LBX2 | 2.462445 | -0.53387 | 7.838039 | 1.70E-12 | 1.07E-10 | 17.87752 | UP |
| VSIG10L | -3.56151 | 4.137109 | -7.82998 | 1.78E-12 | 1.10E-10 | 17.78396 | DOWN |
| MUC15 | -3.25981 | 2.429116 | -7.8067 | 2.01E-12 | 1.23E-10 | 17.72825 | DOWN |
| CRISP2 | -4.63246 | -4.73339 | -7.77697 | 2.36E-12 | 1.40E-10 | 17.61281 | DOWN |
| TF | -3.80512 | 2.049258 | -7.78021 | 2.32E-12 | 1.39E-10 | 17.59486 | DOWN |
| ABLIM3 | -2.50454 | 3.504825 | -7.78223 | 2.29E-12 | 1.38E-10 | 17.56187 | DOWN |
| MACC1 | -2.59958 | 3.832814 | -7.77918 | 2.33E-12 | 1.39E-10 | 17.52903 | DOWN |
| KRT222 | -3.19635 | -4.9291 | -7.7686 | 2.46E-12 | 1.45E-10 | 17.52238 | DOWN |
| C7 | -3.63464 | 0.146781 | -7.7372 | 2.91E-12 | 1.69E-10 | 17.48977 | DOWN |
| SPARC | 2.766756 | 9.590343 | 7.756479 | 2.63E-12 | 1.53E-10 | 17.37803 | UP |
| UPK3B | -3.27067 | 2.614022 | -7.7217 | 3.16E-12 | 1.82E-10 | 17.27251 | DOWN |
| LHX5 | 5.315691 | 0.250337 | 7.698779 | 3.57E-12 | 2.04E-10 | 17.17978 | UP |
| TMPRSS2 | -3.62811 | 2.886889 | -7.69864 | 3.58E-12 | 2.04E-10 | 17.13206 | DOWN |
| PPP1R1A | -4.3242 | -2.9893 | -7.65027 | 4.62E-12 | 2.58E-10 | 17.04559 | DOWN |
| COL3A1 | 3.706224 | 10.34929 | 7.681002 | 3.93E-12 | 2.21E-10 | 16.98463 | UP |
| HCN1 | -3.04147 | -5.53864 | -7.65629 | 4.48E-12 | 2.51E-10 | 16.95682 | DOWN |
| AQP7 | -3.39774 | -2.29433 | -7.62183 | 5.37E-12 | 2.95E-10 | 16.89587 | DOWN |
| GULP1 | -2.50559 | 2.492296 | -7.6339 | 5.04E-12 | 2.80E-10 | 16.84744 | DOWN |
| TNXB | -2.82047 | 2.88801 | -7.63315 | 5.06E-12 | 2.80E-10 | 16.8059 | DOWN |
| RNF222 | -3.81932 | 0.877782 | -7.61385 | 5.60E-12 | 3.06E-10 | 16.80511 | DOWN |
| FUT3 | -3.27391 | 4.271525 | -7.63923 | 4.90E-12 | 2.73E-10 | 16.78263 | DOWN |
| MAMDC2 | -3.00579 | 0.837272 | -7.59909 | 6.06E-12 | 3.29E-10 | 16.76659 | DOWN |
| LRRC2 | -3.60448 | -1.41795 | -7.5508 | 7.82E-12 | 4.14E-10 | 16.56287 | DOWN |
| PPARG | -2.56785 | 1.398924 | -7.54253 | 8.17E-12 | 4.30E-10 | 16.45974 | DOWN |
| COL6A3 | 3.499055 | 8.61618 | 7.573597 | 6.93E-12 | 3.72E-10 | 16.45326 | UP |
| PLP1 | -3.61325 | -1.95551 | -7.51183 | 9.60E-12 | 4.96E-10 | 16.35578 | DOWN |
| PIRT | -3.25624 | -5.27285 | -7.53247 | 8.61E-12 | 4.51E-10 | 16.34075 | DOWN |
| TTC9 | -2.97893 | 5.044585 | -7.54724 | 7.97E-12 | 4.21E-10 | 16.29385 | DOWN |
| CYSRT1 | -3.88259 | 3.533298 | -7.52142 | 9.13E-12 | 4.74E-10 | 16.18783 | DOWN |
| IL12A | -2.92166 | -1.50457 | -7.47527 | 1.16E-11 | 5.89E-10 | 16.16685 | DOWN |
| PRH2 | -6.74865 | -2.12999 | -7.48329 | 1.12E-11 | 5.68E-10 | 16.16656 | DOWN |
| SLC13A4 | -2.64152 | 1.133508 | -7.45084 | 1.32E-11 | 6.65E-10 | 16.00394 | DOWN |
| C6 | -4.14952 | -4.25044 | -7.4353 | 1.44E-11 | 7.10E-10 | 15.90173 | DOWN |
| AMOT | -2.74288 | 2.675771 | -7.44581 | 1.36E-11 | 6.78E-10 | 15.85015 | DOWN |
| LMO7DN | -2.72204 | -5.06406 | -7.41693 | 1.58E-11 | 7.77E-10 | 15.76661 | DOWN |
| ST6GALNAC1 | -3.41675 | 3.517457 | -7.42456 | 1.52E-11 | 7.49E-10 | 15.68864 | DOWN |
| ISL2 | 3.569627 | -0.15622 | 7.364193 | 2.08E-11 | 1.01E-09 | 15.51289 | UP |
| CDX1 | 3.157446 | -1.41483 | 7.352255 | 2.22E-11 | 1.06E-09 | 15.44531 | UP |
| C15orf48 | -2.45835 | 4.15665 | -7.37959 | 1.92E-11 | 9.33E-10 | 15.44453 | DOWN |
| FAM180B | -2.86169 | -5.01049 | -7.34187 | 2.34E-11 | 1.11E-09 | 15.39653 | DOWN |
| SLC9A4 | -4.37598 | -0.70671 | -7.32273 | 2.59E-11 | 1.22E-09 | 15.38101 | DOWN |
| AR | -2.36453 | 1.192477 | -7.30333 | 2.86E-11 | 1.34E-09 | 15.25915 | DOWN |
| PAX9 | -2.88845 | 4.736694 | -7.34371 | 2.32E-11 | 1.10E-09 | 15.24557 | DOWN |
| EDAR | -2.94279 | 1.116513 | -7.29641 | 2.97E-11 | 1.38E-09 | 15.19918 | DOWN |
| MT1A | -3.35262 | -3.03653 | -7.28853 | 3.09E-11 | 1.43E-09 | 15.19413 | DOWN |
| C1QTNF7 | -2.91302 | -1.81402 | -7.27839 | 3.26E-11 | 1.50E-09 | 15.17175 | DOWN |
| EYA2 | -3.14564 | 3.618796 | -7.29341 | 3.02E-11 | 1.40E-09 | 15.01291 | DOWN |
| UGT1A8 | -5.14932 | -2.17455 | -7.22517 | 4.30E-11 | 1.93E-09 | 14.91387 | DOWN |
| SOX11 | 4.44207 | -0.45208 | 7.242507 | 3.93E-11 | 1.78E-09 | 14.91079 | UP |
| PRR15L | -2.94858 | 1.207142 | -7.23837 | 4.02E-11 | 1.81E-09 | 14.89769 | DOWN |
| AQP4 | -4.35051 | -3.28474 | -7.20988 | 4.66E-11 | 2.07E-09 | 14.82588 | DOWN |
| IL22 | -3.68579 | -5.19948 | -7.22065 | 4.40E-11 | 1.96E-09 | 14.8026 | DOWN |
| SCEL | -4.12762 | 5.071279 | -7.25425 | 3.70E-11 | 1.68E-09 | 14.79332 | DOWN |
| HOXC13 | 6.113601 | 1.85626 | 7.215442 | 4.52E-11 | 2.01E-09 | 14.79233 | UP |
| UGT1A10 | -4.80221 | -0.94462 | -7.20297 | 4.83E-11 | 2.14E-09 | 14.76535 | DOWN |
| TTYH1 | -2.39653 | -0.1263 | -7.19106 | 5.13E-11 | 2.24E-09 | 14.74733 | DOWN |
| FKSG48 | 3.669407 | -0.5561 | 7.202307 | 4.84E-11 | 2.14E-09 | 14.71361 | UP |
| PDK4 | -2.66332 | 2.238327 | -7.15433 | 6.21E-11 | 2.65E-09 | 14.39805 | DOWN |
| ZDHHC15 | -2.42572 | -1.20269 | -7.10468 | 8.03E-11 | 3.34E-09 | 14.31306 | DOWN |
| CHRDL1 | -3.62869 | -0.03063 | -7.10564 | 7.99E-11 | 3.33E-09 | 14.27384 | DOWN |
| ATP10B | -3.2297 | 3.174588 | -7.14337 | 6.57E-11 | 2.78E-09 | 14.26616 | DOWN |
| IYD | -3.6872 | -2.55538 | -7.09083 | 8.62E-11 | 3.57E-09 | 14.23976 | DOWN |
| ZAN | 4.58864 | -0.66213 | 7.08806 | 8.75E-11 | 3.61E-09 | 14.15542 | UP |
| MGLL | -2.3974 | 6.030631 | -7.12743 | 7.14E-11 | 3.00E-09 | 14.13414 | DOWN |
| CLDN10 | -4.23786 | -0.19691 | -7.07683 | 9.27E-11 | 3.81E-09 | 14.11583 | DOWN |
| COL1A2 | 3.201276 | 10.25032 | 7.119988 | 7.42E-11 | 3.11E-09 | 14.09368 | UP |
| HOXA11 | 4.493562 | -0.66356 | 7.064214 | 9.89E-11 | 4.04E-09 | 14.03944 | UP |
| PI16 | -3.239 | -0.20075 | -7.04417 | 1.10E-10 | 4.46E-09 | 13.99545 | DOWN |
| RNF225 | -2.86378 | -0.66284 | -7.00968 | 1.31E-10 | 5.22E-09 | 13.84689 | DOWN |
| GUCY2C | -2.46816 | 0.36573 | -7.00539 | 1.34E-10 | 5.31E-09 | 13.80649 | DOWN |
| ASB12 | -3.42816 | -3.61885 | -7.00453 | 1.34E-10 | 5.32E-09 | 13.78025 | DOWN |
| SYT8 | -3.36754 | 2.701394 | -7.03261 | 1.16E-10 | 4.70E-09 | 13.72832 | DOWN |
| SDPR | -2.41498 | 2.876179 | -7.02777 | 1.19E-10 | 4.80E-09 | 13.71968 | DOWN |
| BMP3 | -4.14695 | -0.79265 | -6.98428 | 1.49E-10 | 5.79E-09 | 13.69351 | DOWN |
| HOPX | -3.45423 | 5.394537 | -7.03838 | 1.13E-10 | 4.58E-09 | 13.69019 | DOWN |
| MMRN1 | -2.78977 | 1.018059 | -6.99402 | 1.42E-10 | 5.57E-09 | 13.68618 | DOWN |
| C9orf66 | -2.55977 | -2.61132 | -6.98126 | 1.51E-10 | 5.86E-09 | 13.67427 | DOWN |
| ZBTB16 | -3.57402 | -1.32711 | -6.97161 | 1.59E-10 | 6.13E-09 | 13.65886 | DOWN |
| ODF4 | -3.32876 | -5.30155 | -6.98306 | 1.50E-10 | 5.81E-09 | 13.6453 | DOWN |
| SGIP1 | 2.893325 | 1.32387 | 6.960461 | 1.68E-10 | 6.47E-09 | 13.55647 | UP |
| COL6A1 | 2.653615 | 8.601776 | 7.003304 | 1.35E-10 | 5.34E-09 | 13.51039 | UP |
| TRPV6 | -2.945 | 0.019989 | -6.94147 | 1.86E-10 | 7.04E-09 | 13.48787 | DOWN |
| TMEM100 | -2.72796 | -1.54499 | -6.92813 | 1.99E-10 | 7.45E-09 | 13.44441 | DOWN |
| SRD5A2 | -3.59722 | -2.89045 | -6.92918 | 1.98E-10 | 7.44E-09 | 13.4386 | DOWN |
| LAMC2 | 3.215862 | 9.565802 | 6.988762 | 1.46E-10 | 5.68E-09 | 13.43252 | UP |
| COL5A2 | 3.481762 | 7.424737 | 6.952275 | 1.76E-10 | 6.69E-09 | 13.36311 | UP |
| WDR66 | 2.492243 | 5.35821 | 6.918184 | 2.09E-10 | 7.79E-09 | 13.31244 | UP |
| SPINK5 | -4.31099 | 6.668833 | -6.95917 | 1.70E-10 | 6.50E-09 | 13.31124 | DOWN |
| SLC2A6 | 2.698143 | 2.783889 | 6.899446 | 2.30E-10 | 8.50E-09 | 13.30249 | UP |
| GAST | 4.76424 | -0.6285 | 6.906766 | 2.22E-10 | 8.22E-09 | 13.2787 | UP |
| TGM3 | -6.33031 | 4.427912 | -6.94705 | 1.80E-10 | 6.86E-09 | 13.2658 | DOWN |
| CACNB4 | -2.49208 | -0.17963 | -6.88282 | 2.50E-10 | 9.07E-09 | 13.21793 | DOWN |
| CXCL17 | -3.76805 | 4.192082 | -6.93507 | 1.92E-10 | 7.26E-09 | 13.1818 | DOWN |
| ARHGAP40 | -3.00763 | 0.770654 | -6.88082 | 2.53E-10 | 9.13E-09 | 13.13924 | DOWN |
| SPINK7 | -4.76989 | 1.014068 | -6.88689 | 2.45E-10 | 8.94E-09 | 13.05878 | DOWN |
| MAB21L3 | -3.22076 | 0.742999 | -6.86684 | 2.72E-10 | 9.72E-09 | 13.05669 | DOWN |
| HLF | -2.41031 | 2.82459 | -6.88367 | 2.49E-10 | 9.07E-09 | 13.00381 | DOWN |
| HMGCS2 | -4.33299 | -2.96461 | -6.81474 | 3.54E-10 | 1.24E-08 | 12.89211 | DOWN |
| GNG4 | -2.58321 | 2.904736 | -6.86279 | 2.77E-10 | 9.91E-09 | 12.88791 | DOWN |
| STATH | -5.5605 | -1.02981 | -6.83291 | 3.23E-10 | 1.14E-08 | 12.88185 | DOWN |
| HSPB6 | -3.02777 | 1.703109 | -6.84634 | 3.01E-10 | 1.07E-08 | 12.87964 | DOWN |
| CIDEC | -3.70301 | -4.07724 | -6.79549 | 3.90E-10 | 1.35E-08 | 12.76485 | DOWN |
| COL13A1 | 2.718018 | 2.143038 | 6.78516 | 4.11E-10 | 1.42E-08 | 12.73522 | UP |
| GALNT5 | -2.85692 | 3.575323 | -6.83347 | 3.22E-10 | 1.14E-08 | 12.69396 | DOWN |
| KRT4 | -7.65597 | 5.841823 | -6.82068 | 3.43E-10 | 1.21E-08 | 12.65899 | DOWN |
| TNMD | -2.43138 | -5.88685 | -6.77088 | 4.42E-10 | 1.51E-08 | 12.62376 | DOWN |
| TPPP2 | -2.65128 | -5.14873 | -6.76522 | 4.55E-10 | 1.55E-08 | 12.59849 | DOWN |
| C15orf59 | -2.49829 | 1.727379 | -6.77454 | 4.34E-10 | 1.48E-08 | 12.56124 | DOWN |
| SERPINB1 | -2.40199 | 7.805878 | -6.80432 | 3.73E-10 | 1.30E-08 | 12.52872 | DOWN |
| DLX1 | 4.172743 | 0.481013 | 6.748025 | 4.96E-10 | 1.67E-08 | 12.52404 | UP |
| BGN | 2.669335 | 8.0779 | 6.79031 | 4.01E-10 | 1.39E-08 | 12.46009 | UP |
| CD300LG | -2.8937 | -3.80982 | -6.72919 | 5.46E-10 | 1.82E-08 | 12.43605 | DOWN |
| OMP | -2.47349 | -5.33148 | -6.72303 | 5.63E-10 | 1.87E-08 | 12.39694 | DOWN |
| DHRS9 | -2.96079 | 3.046497 | -6.75495 | 4.79E-10 | 1.62E-08 | 12.33123 | DOWN |
| SHOX2 | 3.946504 | 1.563275 | 6.703426 | 6.21E-10 | 2.05E-08 | 12.31772 | UP |
| NT5DC4 | 2.740839 | -2.23092 | 6.692698 | 6.56E-10 | 2.15E-08 | 12.25132 | UP |
| B3GALT5 | -3.6358 | 0.530282 | -6.69705 | 6.42E-10 | 2.11E-08 | 12.20653 | DOWN |
| SLC4A4 | -2.868 | -0.36238 | -6.66648 | 7.48E-10 | 2.43E-08 | 12.15766 | DOWN |
| CA10 | -2.89116 | -5.13429 | -6.67019 | 7.35E-10 | 2.39E-08 | 12.14729 | DOWN |
| ISL1 | -3.10032 | -0.91022 | -6.63374 | 8.82E-10 | 2.81E-08 | 12.01063 | DOWN |
| SOWAHB | -2.3612 | 2.67591 | -6.67629 | 7.12E-10 | 2.33E-08 | 11.99332 | DOWN |
| PACRG | -3.21802 | -2.32194 | -6.62837 | 9.06E-10 | 2.88E-08 | 11.99108 | DOWN |
| MYRIP | -2.42671 | -1.16722 | -6.62462 | 9.23E-10 | 2.93E-08 | 11.97551 | DOWN |
| OGN | -3.19815 | 0.121114 | -6.61777 | 9.56E-10 | 3.03E-08 | 11.87911 | DOWN |
| KRT33A | -3.91645 | -2.92013 | -6.59226 | 1.09E-09 | 3.37E-08 | 11.81882 | DOWN |
| GBP6 | -2.85609 | 6.361302 | -6.65454 | 7.95E-10 | 2.56E-08 | 11.77621 | DOWN |
| KEL | -3.26126 | -2.12824 | -6.58024 | 1.15E-09 | 3.56E-08 | 11.76268 | DOWN |
| TIMP4 | -2.59724 | -1.264 | -6.57048 | 1.21E-09 | 3.72E-08 | 11.71631 | DOWN |
| KLB | -2.38142 | -1.58336 | -6.56325 | 1.26E-09 | 3.85E-08 | 11.6803 | DOWN |
| UBXN10 | -2.60852 | 0.192234 | -6.56947 | 1.22E-09 | 3.74E-08 | 11.67206 | DOWN |
| UGT1A7 | -4.24943 | 1.164621 | -6.59318 | 1.08E-09 | 3.36E-08 | 11.61538 | DOWN |
| MUC20 | -3.03212 | 3.449416 | -6.59988 | 1.05E-09 | 3.27E-08 | 11.54382 | DOWN |
| GBX2 | 4.492484 | -2.03907 | 6.539246 | 1.41E-09 | 4.27E-08 | 11.53013 | UP |
| SLC6A7 | 2.613392 | -3.44911 | 6.517784 | 1.57E-09 | 4.70E-08 | 11.42807 | UP |
| CERCAM | 2.556978 | 5.621469 | 6.538544 | 1.42E-09 | 4.28E-08 | 11.42391 | UP |
| PEBP4 | -3.5147 | -3.35741 | -6.50378 | 1.69E-09 | 4.98E-08 | 11.38925 | DOWN |
| STX19 | -2.37912 | 0.687321 | -6.51014 | 1.64E-09 | 4.84E-08 | 11.36975 | DOWN |
| COL5A1 | 3.318829 | 7.855544 | 6.548307 | 1.35E-09 | 4.10E-08 | 11.31575 | UP |
| OFCC1 | 3.687179 | -2.51803 | 6.487209 | 1.83E-09 | 5.37E-08 | 11.28524 | UP |
| CNGB3 | 2.921161 | -1.59172 | 6.483693 | 1.86E-09 | 5.46E-08 | 11.26802 | UP |
| MLIP | -2.80819 | 0.416813 | -6.47829 | 1.92E-09 | 5.56E-08 | 11.21193 | DOWN |
| EPS8L1 | -2.9334 | 5.555427 | -6.53588 | 1.44E-09 | 4.32E-08 | 11.19068 | DOWN |
| ABCA8 | -2.5703 | 0.992814 | -6.44027 | 2.31E-09 | 6.50E-08 | 10.98902 | DOWN |
| KRT24 | -5.1833 | 1.090543 | -6.46157 | 2.08E-09 | 5.95E-08 | 10.95207 | DOWN |
| CFTR | -3.34974 | -0.61332 | -6.41488 | 2.62E-09 | 7.27E-08 | 10.9433 | DOWN |
| NOX4 | 2.615378 | 1.905308 | 6.407098 | 2.72E-09 | 7.51E-08 | 10.93668 | UP |
| EHF | -2.5989 | 6.387483 | -6.47326 | 1.96E-09 | 5.70E-08 | 10.88751 | DOWN |
| PLAU | 2.377198 | 7.516763 | 6.465763 | 2.04E-09 | 5.87E-08 | 10.88045 | UP |
| CLIC3 | -2.99023 | 3.873965 | -6.46372 | 2.06E-09 | 5.91E-08 | 10.86582 | DOWN |
| CHRNA6 | 2.925773 | -2.85566 | 6.395593 | 2.88E-09 | 7.94E-08 | 10.85745 | UP |
| CYP2C18 | -2.86879 | 2.63539 | -6.44598 | 2.25E-09 | 6.35E-08 | 10.85014 | DOWN |
| MALL | -2.50984 | 3.936354 | -6.45212 | 2.18E-09 | 6.20E-08 | 10.81108 | DOWN |
| COL12A1 | 2.675102 | 7.710172 | 6.43637 | 2.36E-09 | 6.62E-08 | 10.74215 | UP |
| MFAP2 | 3.41046 | 5.10211 | 6.374204 | 3.20E-09 | 8.69E-08 | 10.74147 | UP |
| COL6A2 | 2.386982 | 8.88882 | 6.435114 | 2.37E-09 | 6.64E-08 | 10.69563 | UP |
| SLURP1 | -4.50233 | 2.277269 | -6.41749 | 2.59E-09 | 7.20E-08 | 10.69495 | DOWN |
| ADARB2 | -3.22652 | -0.69533 | -6.34395 | 3.72E-09 | 9.95E-08 | 10.61451 | DOWN |
| EYA1 | -3.00279 | -0.50432 | -6.33532 | 3.88E-09 | 1.03E-07 | 10.57294 | DOWN |
| STMND1 | -3.60134 | -4.0656 | -6.32728 | 4.04E-09 | 1.07E-07 | 10.55365 | DOWN |
| IBSP | 4.139632 | -0.95429 | 6.328603 | 4.01E-09 | 1.06E-07 | 10.5486 | UP |
| THY1 | 3.143665 | 5.771294 | 6.340698 | 3.78E-09 | 1.01E-07 | 10.51224 | UP |
| DIO1 | -2.92703 | -2.806 | -6.31532 | 4.28E-09 | 1.13E-07 | 10.50694 | DOWN |
| TNFRSF4 | 2.631425 | 1.712681 | 6.312611 | 4.34E-09 | 1.14E-07 | 10.49312 | UP |
| ANKRD20A1 | -2.67726 | -5.1857 | -6.31627 | 4.26E-09 | 1.12E-07 | 10.49123 | DOWN |
| ADAMTS6 | 2.597346 | 0.326229 | 6.314627 | 4.29E-09 | 1.13E-07 | 10.48923 | UP |
| ATP2B2 | -2.85066 | -2.12632 | -6.30906 | 4.41E-09 | 1.15E-07 | 10.48203 | DOWN |
| ANGPTL5 | -2.61943 | -5.17656 | -6.30067 | 4.60E-09 | 1.19E-07 | 10.41919 | DOWN |
| HOXC8 | 4.258868 | -0.14645 | 6.292217 | 4.79E-09 | 1.23E-07 | 10.38283 | UP |
| HOXD10 | 4.230096 | 2.361417 | 6.288248 | 4.89E-09 | 1.25E-07 | 10.37362 | UP |
| GPC2 | 2.716406 | 0.196929 | 6.289282 | 4.86E-09 | 1.25E-07 | 10.37166 | UP |
| ATP1A2 | -3.58632 | -0.02391 | -6.29279 | 4.78E-09 | 1.23E-07 | 10.30774 | DOWN |
| BARX2 | -3.24676 | 4.061462 | -6.34768 | 3.65E-09 | 9.78E-08 | 10.29804 | DOWN |
| CCDC160 | -3.57509 | -3.19884 | -6.26885 | 5.37E-09 | 1.37E-07 | 10.29296 | DOWN |
| PXDN | 2.77274 | 6.448656 | 6.301819 | 4.57E-09 | 1.19E-07 | 10.21267 | UP |
| AADAC | -3.54592 | 0.360228 | -6.24662 | 5.99E-09 | 1.50E-07 | 10.06474 | DOWN |
| PRH1 | -3.94708 | -1.98176 | -6.21749 | 6.90E-09 | 1.71E-07 | 10.04282 | DOWN |
| LAMB4 | -3.09824 | -1.63904 | -6.19616 | 7.66E-09 | 1.88E-07 | 9.952563 | DOWN |
| MYLK3 | -3.17936 | -2.05133 | -6.17727 | 8.39E-09 | 2.05E-07 | 9.86864 | DOWN |
| BMP8A | 2.534492 | -0.98146 | 6.167052 | 8.82E-09 | 2.13E-07 | 9.807391 | UP |
| SPOCD1 | 3.150595 | 1.541559 | 6.156826 | 9.27E-09 | 2.21E-07 | 9.768628 | UP |
| P4HA3 | 2.884104 | 1.335469 | 6.154541 | 9.37E-09 | 2.23E-07 | 9.758308 | UP |
| PLA2G7 | 2.964141 | 2.30321 | 6.143878 | 9.87E-09 | 2.33E-07 | 9.716674 | UP |
| C4orf19 | -2.72361 | 0.672943 | -6.16478 | 8.92E-09 | 2.15E-07 | 9.706049 | DOWN |
| C1QL4 | 3.249162 | -2.49722 | 6.127324 | 1.07E-08 | 2.51E-07 | 9.624813 | UP |
| BNIPL | -2.43024 | 4.340426 | -6.16795 | 8.78E-09 | 2.12E-07 | 9.437013 | DOWN |
| PPAPDC1A | 4.243964 | 0.444581 | 6.076108 | 1.37E-08 | 3.12E-07 | 9.396537 | UP |
| GGT6 | -2.58267 | 4.576139 | -6.15627 | 9.29E-09 | 2.22E-07 | 9.374492 | DOWN |
| FAM132B | 3.363811 | 0.761463 | 6.070087 | 1.41E-08 | 3.21E-07 | 9.370518 | UP |
| LOXL2 | 3.318216 | 5.648351 | 6.088168 | 1.29E-08 | 2.97E-07 | 9.346648 | UP |
| ASPG | -2.73732 | 2.610335 | -6.12789 | 1.07E-08 | 2.51E-07 | 9.344355 | DOWN |
| ZFP92 | 2.833133 | 1.00895 | 6.062948 | 1.46E-08 | 3.31E-07 | 9.339412 | UP |
| FKSG52 | 2.665921 | -3.26507 | 6.063112 | 1.46E-08 | 3.31E-07 | 9.334008 | UP |
| WISP1 | 3.459617 | 2.887515 | 6.030853 | 1.70E-08 | 3.77E-07 | 9.199991 | UP |
| C14orf180 | -2.40182 | -6.09411 | -6.02773 | 1.73E-08 | 3.82E-07 | 9.174002 | DOWN |
| CADM2 | -3.14758 | -2.96338 | -6.02129 | 1.78E-08 | 3.90E-07 | 9.156689 | DOWN |
| HSPB8 | -2.63939 | 5.697802 | -6.09968 | 1.22E-08 | 2.82E-07 | 9.096893 | DOWN |
| HOXB7 | 3.170523 | 3.193896 | 5.987185 | 2.10E-08 | 4.50E-07 | 8.996991 | UP |
| RNF223 | -2.44778 | 1.900799 | -6.03325 | 1.68E-08 | 3.74E-07 | 8.992192 | DOWN |
| CRTAC1 | -2.74945 | 0.629266 | -6.00807 | 1.90E-08 | 4.14E-07 | 8.977841 | DOWN |
| HES7 | 3.05974 | -2.75695 | 5.982081 | 2.15E-08 | 4.59E-07 | 8.968939 | UP |
| PCDH17 | 2.746416 | 2.590117 | 5.977522 | 2.20E-08 | 4.67E-07 | 8.95557 | UP |
| ARSH | -2.77923 | -3.93912 | -5.97148 | 2.26E-08 | 4.78E-07 | 8.923784 | DOWN |
| HOXC11 | 5.36431 | -0.18751 | 5.96969 | 2.28E-08 | 4.81E-07 | 8.915437 | UP |
| SNX10 | 2.647623 | 3.454482 | 5.972854 | 2.24E-08 | 4.76E-07 | 8.913677 | UP |
| MUC4 | -4.37349 | 4.930588 | -6.05575 | 1.51E-08 | 3.41E-07 | 8.908553 | DOWN |
| MCEMP1 | 2.812633 | -1.23772 | 5.954268 | 2.45E-08 | 5.12E-07 | 8.844847 | UP |
| ESRRG | -3.03353 | -1.24126 | -5.95582 | 2.43E-08 | 5.09E-07 | 8.83793 | DOWN |
| IL11 | 4.136832 | 1.858734 | 5.947984 | 2.53E-08 | 5.26E-07 | 8.821599 | UP |
| CDH22 | -3.44654 | -1.91804 | -5.94947 | 2.51E-08 | 5.23E-07 | 8.818371 | DOWN |
| CCL23 | -2.46249 | -2.78418 | -5.93513 | 2.69E-08 | 5.56E-07 | 8.766291 | DOWN |
| FADS1 | 2.902516 | 5.25443 | 5.957407 | 2.42E-08 | 5.06E-07 | 8.742814 | UP |
| LINGO4 | -2.89434 | -4.28376 | -5.92915 | 2.76E-08 | 5.71E-07 | 8.733352 | DOWN |
| PCP4L1 | -3.02819 | 2.120225 | -5.98895 | 2.08E-08 | 4.48E-07 | 8.714367 | DOWN |
| LUM | 2.745926 | 7.723452 | 6.004919 | 1.93E-08 | 4.19E-07 | 8.693848 | UP |
| LDLRAD1 | -3.84614 | -1.91308 | -5.91798 | 2.91E-08 | 5.99E-07 | 8.665652 | DOWN |
| ACADL | -2.78192 | -2.07242 | -5.90818 | 3.05E-08 | 6.23E-07 | 8.643327 | DOWN |
| PDGFRB | 2.36255 | 6.462566 | 5.971154 | 2.26E-08 | 4.78E-07 | 8.613119 | UP |
| CHAD | -2.42639 | -1.58059 | -5.90053 | 3.17E-08 | 6.41E-07 | 8.607117 | DOWN |
| CD80 | 2.393934 | -0.28388 | 5.898369 | 3.20E-08 | 6.47E-07 | 8.596977 | UP |
| NLRP14 | -2.36436 | -4.19312 | -5.89496 | 3.25E-08 | 6.57E-07 | 8.580429 | DOWN |
| TMPRSS11A | -3.91947 | 4.348594 | -5.97345 | 2.24E-08 | 4.75E-07 | 8.523468 | DOWN |
| ZG16B | -3.49559 | 2.088589 | -5.93802 | 2.65E-08 | 5.49E-07 | 8.459576 | DOWN |
| CTHRC1 | 4.057594 | 4.709229 | 5.863915 | 3.77E-08 | 7.41E-07 | 8.417828 | UP |
| KBTBD12 | -3.72271 | -3.2957 | -5.85433 | 3.94E-08 | 7.71E-07 | 8.404181 | DOWN |
| PIP | -5.09175 | -2.26032 | -5.86394 | 3.76E-08 | 7.41E-07 | 8.384662 | DOWN |
| DLX2 | 3.886632 | -0.23105 | 5.844895 | 4.12E-08 | 8.00E-07 | 8.358686 | UP |
| CIDEA | -3.65032 | -1.74572 | -5.84655 | 4.09E-08 | 7.96E-07 | 8.343054 | DOWN |
| DKK4 | -3.56387 | -2.58552 | -5.84007 | 4.21E-08 | 8.14E-07 | 8.335174 | DOWN |
| C1orf168 | -3.01744 | -3.58291 | -5.83832 | 4.25E-08 | 8.19E-07 | 8.33251 | DOWN |
| PRB3 | -4.44844 | -0.81974 | -5.86596 | 3.73E-08 | 7.36E-07 | 8.327956 | DOWN |
| LHX2 | 3.875588 | 0.139078 | 5.830156 | 4.42E-08 | 8.45E-07 | 8.293891 | UP |
| RHBG | -2.72685 | -1.21317 | -5.83194 | 4.38E-08 | 8.40E-07 | 8.286448 | DOWN |
| SORCS1 | -2.93968 | -2.90475 | -5.82093 | 4.61E-08 | 8.80E-07 | 8.256206 | DOWN |
| MUC1 | -2.60556 | 5.890763 | -5.91024 | 3.02E-08 | 6.19E-07 | 8.212675 | DOWN |
| LRFN2 | -3.02536 | -3.62231 | -5.81088 | 4.84E-08 | 9.18E-07 | 8.210547 | DOWN |
| OXGR1 | -2.94701 | -0.41747 | -5.82059 | 4.62E-08 | 8.80E-07 | 8.188667 | DOWN |
| NTF3 | -2.49079 | -0.25167 | -5.81552 | 4.73E-08 | 9.00E-07 | 8.179427 | DOWN |
| DEGS2 | -2.36043 | 2.579812 | -5.8711 | 3.64E-08 | 7.21E-07 | 8.171982 | DOWN |
| ELANE | -2.50285 | -4.35565 | -5.79499 | 5.21E-08 | 9.81E-07 | 8.137222 | DOWN |
| PKHD1L1 | -2.52888 | -2.09496 | -5.77701 | 5.67E-08 | 1.05E-06 | 8.058678 | DOWN |
| C6orf10 | 4.155001 | -1.59101 | 5.773664 | 5.76E-08 | 1.07E-06 | 8.042812 | UP |
| PRR4 | -4.38916 | 0.940602 | -5.823 | 4.57E-08 | 8.72E-07 | 7.978203 | DOWN |
| GJC1 | 3.230447 | 3.04063 | 5.757966 | 6.20E-08 | 1.15E-06 | 7.96873 | UP |
| METTL11B | 2.924824 | -3.43438 | 5.755311 | 6.28E-08 | 1.16E-06 | 7.962312 | UP |
| AQP2 | -3.11037 | -4.88807 | -5.74745 | 6.51E-08 | 1.19E-06 | 7.927996 | DOWN |
| RERGL | -2.72583 | -3.85689 | -5.74472 | 6.59E-08 | 1.20E-06 | 7.91683 | DOWN |
| ZIC2 | 4.236127 | 2.187771 | 5.733097 | 6.96E-08 | 1.26E-06 | 7.867336 | UP |
| HIST1H2BO | 2.775483 | -2.60106 | 5.731055 | 7.03E-08 | 1.27E-06 | 7.855335 | UP |
| CTTNBP2 | -2.40484 | 1.235167 | -5.76968 | 5.87E-08 | 1.09E-06 | 7.843415 | DOWN |
| GPR111 | -2.63906 | -0.74649 | -5.73179 | 7.01E-08 | 1.27E-06 | 7.823822 | DOWN |
| DLX6 | 4.134681 | 0.732897 | 5.713781 | 7.62E-08 | 1.37E-06 | 7.781641 | UP |
| SNCB | 3.317441 | -1.57371 | 5.709477 | 7.77E-08 | 1.39E-06 | 7.761036 | UP |
| CLDN14 | 2.895691 | -1.35091 | 5.697568 | 8.22E-08 | 1.46E-06 | 7.709158 | UP |
| EXTL1 | -2.92429 | -3.01442 | -5.69275 | 8.40E-08 | 1.49E-06 | 7.689816 | DOWN |
| CCL11 | 4.176833 | 0.132671 | 5.689176 | 8.54E-08 | 1.50E-06 | 7.673486 | UP |
| SCGN | -2.57508 | -5.59086 | -5.68929 | 8.54E-08 | 1.50E-06 | 7.672925 | DOWN |
| GABRA4 | -2.96557 | -4.94235 | -5.68797 | 8.59E-08 | 1.51E-06 | 7.667523 | DOWN |
| SLC11A1 | 2.413108 | 2.955386 | 5.692317 | 8.42E-08 | 1.49E-06 | 7.66345 | UP |
| ANKRD20A4 | -2.73111 | -4.93011 | -5.68496 | 8.71E-08 | 1.53E-06 | 7.654279 | DOWN |
| SCG3 | -2.92721 | -3.49746 | -5.6432 | 1.06E-07 | 1.82E-06 | 7.473756 | DOWN |
| CKMT2 | -2.83801 | -0.40232 | -5.6585 | 9.85E-08 | 1.71E-06 | 7.468049 | DOWN |
| SLC17A9 | 2.561664 | 2.4228 | 5.641794 | 1.06E-07 | 1.83E-06 | 7.456374 | UP |
| HOXB9 | 4.678724 | -0.38901 | 5.626331 | 1.14E-07 | 1.95E-06 | 7.399487 | UP |
| FKBP10 | 2.699538 | 5.990605 | 5.682184 | 8.83E-08 | 1.54E-06 | 7.38375 | UP |
| FOXI2 | -3.48483 | -3.30162 | -5.62192 | 1.17E-07 | 1.99E-06 | 7.37852 | DOWN |
| CLVS1 | -2.41415 | -2.48454 | -5.61516 | 1.20E-07 | 2.04E-06 | 7.350105 | DOWN |
| TDO2 | 3.132859 | 1.252949 | 5.598922 | 1.30E-07 | 2.19E-06 | 7.281289 | UP |
| PLCXD3 | -3.05289 | -3.46335 | -5.59644 | 1.31E-07 | 2.21E-06 | 7.270216 | DOWN |
| SFTPA2 | -3.41678 | -3.05781 | -5.57211 | 1.47E-07 | 2.44E-06 | 7.159809 | DOWN |
| ADH1C | -3.81584 | -0.15993 | -5.6003 | 1.29E-07 | 2.18E-06 | 7.121555 | DOWN |
| MUC7 | -4.815 | -3.49244 | -5.56481 | 1.52E-07 | 2.51E-06 | 7.112977 | DOWN |
| ADAMTS2 | 3.733889 | 5.551866 | 5.568769 | 1.49E-07 | 2.48E-06 | 7.029819 | UP |
| SHISA3 | -3.06449 | -4.00452 | -5.53919 | 1.71E-07 | 2.78E-06 | 7.023215 | DOWN |
| CCL14 | -2.4313 | -2.07636 | -5.53662 | 1.73E-07 | 2.80E-06 | 7.004488 | DOWN |
| CEACAM5 | -4.58262 | 5.799905 | -5.63918 | 1.08E-07 | 1.84E-06 | 6.998212 | DOWN |
| CTNNA2 | -2.51909 | -4.91085 | -5.51982 | 1.87E-07 | 3.00E-06 | 6.939752 | DOWN |
| CYP3A4 | -2.58748 | -4.39231 | -5.50106 | 2.03E-07 | 3.24E-06 | 6.859388 | DOWN |
| CRCT1 | -3.76565 | 2.696184 | -5.58949 | 1.36E-07 | 2.28E-06 | 6.827452 | DOWN |
| TNFRSF9 | 2.467476 | 1.616495 | 5.492374 | 2.12E-07 | 3.35E-06 | 6.817597 | UP |
| TCF24 | 2.364622 | -2.40887 | 5.489795 | 2.14E-07 | 3.38E-06 | 6.811047 | UP |
| CA3 | -3.26105 | -0.84142 | -5.50467 | 2.00E-07 | 3.20E-06 | 6.79429 | DOWN |
| POSTN | 3.496416 | 7.067697 | 5.544193 | 1.67E-07 | 2.72E-06 | 6.721396 | UP |
| ART4 | -2.41909 | -3.76267 | -5.46868 | 2.36E-07 | 3.72E-06 | 6.721001 | DOWN |
| NRG3 | -2.7745 | -4.44638 | -5.45956 | 2.46E-07 | 3.84E-06 | 6.682098 | DOWN |
| IGF2BP1 | 6.573039 | 1.206408 | 5.455964 | 2.50E-07 | 3.89E-06 | 6.666555 | UP |
| GRIK3 | -3.11178 | -3.31012 | -5.44568 | 2.62E-07 | 4.06E-06 | 6.619867 | DOWN |
| TMPRSS11E | -3.23435 | 4.597952 | -5.55498 | 1.59E-07 | 2.61E-06 | 6.607211 | DOWN |
| SOST | 4.724545 | -1.18742 | 5.439077 | 2.70E-07 | 4.16E-06 | 6.59492 | UP |
| TEX11 | 2.468308 | -2.73705 | 5.43609 | 2.73E-07 | 4.21E-06 | 6.58221 | UP |
| SCN2B | -2.47321 | -1.05765 | -5.44165 | 2.67E-07 | 4.13E-06 | 6.567391 | DOWN |
| LRG1 | -2.66324 | 4.302461 | -5.54077 | 1.70E-07 | 2.76E-06 | 6.551177 | DOWN |
| SERPINB13 | -2.62953 | 6.186233 | -5.53904 | 1.71E-07 | 2.78E-06 | 6.528067 | DOWN |
| KIF26B | 3.203239 | 4.080868 | 5.43144 | 2.79E-07 | 4.29E-06 | 6.498718 | UP |
| C8orf22 | -3.43544 | -5.23696 | -5.40872 | 3.09E-07 | 4.70E-06 | 6.466041 | DOWN |
| SLC5A1 | -2.46847 | 2.823219 | -5.49461 | 2.09E-07 | 3.32E-06 | 6.442321 | DOWN |
| FST | 2.794653 | 5.862189 | 5.457469 | 2.48E-07 | 3.88E-06 | 6.415914 | UP |
| ANGPTL7 | -3.08544 | -3.51009 | -5.39128 | 3.35E-07 | 5.04E-06 | 6.389625 | DOWN |
| DDX25 | -2.76591 | -4.21747 | -5.39021 | 3.36E-07 | 5.06E-06 | 6.387643 | DOWN |
| ENO2 | 2.804455 | 5.117488 | 5.429252 | 2.82E-07 | 4.33E-06 | 6.382437 | UP |
| FSD1 | 2.386371 | -0.72759 | 5.386595 | 3.42E-07 | 5.13E-06 | 6.372293 | UP |
| IL1R2 | -2.55492 | 4.294868 | -5.49698 | 2.07E-07 | 3.29E-06 | 6.357312 | DOWN |
| GPR12 | -2.75204 | -4.55571 | -5.38231 | 3.49E-07 | 5.19E-06 | 6.354296 | DOWN |
| KRT31 | -2.57926 | 1.210879 | -5.42337 | 2.90E-07 | 4.43E-06 | 6.303995 | DOWN |
| FN1 | 3.010838 | 9.384175 | 5.481212 | 2.23E-07 | 3.51E-06 | 6.266851 | UP |
| GPM6A | -2.84691 | -3.42443 | -5.35985 | 3.86E-07 | 5.65E-06 | 6.25724 | DOWN |
| RGS22 | -2.47142 | -2.1391 | -5.36099 | 3.84E-07 | 5.63E-06 | 6.251472 | DOWN |
| CNTD2 | 3.681821 | -0.48385 | 5.354548 | 3.95E-07 | 5.75E-06 | 6.237055 | UP |
| SEMA5B | 2.360207 | 0.568288 | 5.348075 | 4.07E-07 | 5.91E-06 | 6.208737 | UP |
| LINGO1 | 2.451889 | 1.163258 | 5.339856 | 4.22E-07 | 6.12E-06 | 6.171519 | UP |
| ADAMTS12 | 4.045391 | 3.756944 | 5.325289 | 4.51E-07 | 6.48E-06 | 6.086427 | UP |
| C1orf61 | 2.615178 | -2.70989 | 5.310995 | 4.80E-07 | 6.86E-06 | 6.054462 | UP |
| HOXA10 | 2.971767 | 1.949094 | 5.303759 | 4.96E-07 | 7.05E-06 | 6.015709 | UP |
| PRKAA2 | -2.34936 | 1.309497 | -5.3546 | 3.95E-07 | 5.75E-06 | 6.001032 | DOWN |
| C6orf58 | -4.68122 | -2.0606 | -5.30147 | 5.01E-07 | 7.11E-06 | 5.916467 | DOWN |
| SOCS1 | 2.435255 | 3.157652 | 5.286901 | 5.35E-07 | 7.52E-06 | 5.894689 | UP |
| KCNK9 | 2.615893 | -1.91589 | 5.272626 | 5.70E-07 | 7.92E-06 | 5.894079 | UP |
| TM4SF19-TCTEX1D2 | 2.920128 | -3.78173 | 5.272275 | 5.71E-07 | 7.92E-06 | 5.892245 | UP |
| AC009060.2 | 3.081479 | -3.37407 | 5.264197 | 5.92E-07 | 8.15E-06 | 5.858661 | UP |
| TMEM45B | -2.61419 | 4.20234 | -5.38041 | 3.52E-07 | 5.23E-06 | 5.846785 | DOWN |
| RCN3 | 2.580409 | 4.610481 | 5.294626 | 5.17E-07 | 7.31E-06 | 5.831447 | UP |
| HSD17B13 | -2.5398 | -2.86218 | -5.25432 | 6.19E-07 | 8.49E-06 | 5.8114 | DOWN |
| C8orf74 | -3.36551 | -3.48276 | -5.25353 | 6.21E-07 | 8.51E-06 | 5.806665 | DOWN |
| FOS | -2.46864 | 7.918841 | -5.36622 | 3.75E-07 | 5.51E-06 | 5.785294 | DOWN |
| GJB7 | 3.327674 | 0.366373 | 5.246798 | 6.40E-07 | 8.75E-06 | 5.784991 | UP |
| DPF1 | 3.04633 | 1.483279 | 5.240814 | 6.57E-07 | 8.94E-06 | 5.757509 | UP |
| PMEPA1 | 2.463217 | 6.455245 | 5.321649 | 4.58E-07 | 6.57E-06 | 5.705322 | UP |
| MMP1 | 5.23823 | 7.040827 | 5.266364 | 5.86E-07 | 8.09E-06 | 5.704183 | UP |
| MIXL1 | 2.819632 | -1.68504 | 5.22184 | 7.15E-07 | 9.58E-06 | 5.682884 | UP |
| AL589743.1 | 3.276274 | -1.99289 | 5.21979 | 7.21E-07 | 9.64E-06 | 5.674445 | UP |
| NWD1 | -2.75304 | -0.1243 | -5.24961 | 6.32E-07 | 8.66E-06 | 5.674425 | DOWN |
| CPXM1 | 3.119036 | 3.58756 | 5.231621 | 6.84E-07 | 9.25E-06 | 5.668559 | UP |
| S100P | -3.22298 | 4.511088 | -5.32715 | 4.47E-07 | 6.44E-06 | 5.604386 | DOWN |
| SERPINB2 | -3.18033 | 4.81901 | -5.32657 | 4.48E-07 | 6.45E-06 | 5.597432 | DOWN |
| STC2 | 3.564187 | 4.268577 | 5.20347 | 7.75E-07 | 1.03E-05 | 5.5339 | UP |
| SYTL5 | -2.87161 | 0.949867 | -5.22643 | 7.00E-07 | 9.43E-06 | 5.448721 | DOWN |
| SLC5A12 | 3.40095 | 0.391135 | 5.161218 | 9.34E-07 | 1.22E-05 | 5.430452 | UP |
| HOXA13 | 4.01907 | -1.0472 | 5.157942 | 9.48E-07 | 1.23E-05 | 5.418707 | UP |
| ABO | -2.59513 | 3.86679 | -5.27736 | 5.58E-07 | 7.79E-06 | 5.410168 | DOWN |
| SERPINE1 | 2.878908 | 7.437919 | 5.265561 | 5.89E-07 | 8.12E-06 | 5.401582 | UP |
| PAEP | 3.953407 | -2.03725 | 5.152794 | 9.70E-07 | 1.26E-05 | 5.397734 | UP |
| RXFP1 | 3.19289 | -2.39866 | 5.145032 | 1.00E-06 | 1.29E-05 | 5.366036 | UP |
| SLAMF9 | 3.285007 | 0.089537 | 5.144418 | 1.01E-06 | 1.30E-05 | 5.36172 | UP |
| LAIR2 | 2.397792 | -1.77706 | 5.142256 | 1.02E-06 | 1.30E-05 | 5.354563 | UP |
| PRB4 | -3.89887 | -5.10059 | -5.13624 | 1.04E-06 | 1.33E-05 | 5.328908 | DOWN |
| GPR158 | 4.478456 | 0.297889 | 5.134489 | 1.05E-06 | 1.34E-05 | 5.320997 | UP |
| CMA1 | -2.60788 | -2.74433 | -5.13071 | 1.07E-06 | 1.36E-05 | 5.295676 | DOWN |
| ECEL1 | 2.946026 | -1.05377 | 5.126797 | 1.09E-06 | 1.38E-05 | 5.290712 | UP |
| HOXD13 | 6.045528 | -0.06224 | 5.121041 | 1.11E-06 | 1.41E-05 | 5.265972 | UP |
| FAP | 3.405544 | 3.481296 | 5.125924 | 1.09E-06 | 1.38E-05 | 5.240444 | UP |
| SHISA6 | -3.04073 | -1.00877 | -5.12899 | 1.08E-06 | 1.37E-05 | 5.213429 | DOWN |
| LHFPL4 | -2.93055 | -4.13396 | -5.10311 | 1.21E-06 | 1.51E-05 | 5.192938 | DOWN |
| APBA2 | 2.991519 | 2.784047 | 5.110615 | 1.17E-06 | 1.47E-05 | 5.19006 | UP |
| HTR1D | 3.049933 | -1.6249 | 5.0842 | 1.31E-06 | 1.62E-05 | 5.117107 | UP |
| AC007163.2 | 3.314905 | -2.99432 | 5.08365 | 1.31E-06 | 1.62E-05 | 5.114512 | UP |
| LGALS9B | -2.65144 | 0.316789 | -5.12309 | 1.10E-06 | 1.40E-05 | 5.10682 | DOWN |
| CHST2 | 3.319803 | 5.012489 | 5.109498 | 1.17E-06 | 1.47E-05 | 5.072818 | UP |
| PRSS3 | -3.11889 | 2.547546 | -5.17517 | 8.79E-07 | 1.15E-05 | 5.045386 | DOWN |
| IGF2BP2 | 2.763122 | 5.942489 | 5.142604 | 1.01E-06 | 1.30E-05 | 5.043745 | UP |
| SLITRK4 | -3.01088 | -1.06999 | -5.08302 | 1.32E-06 | 1.63E-05 | 5.026762 | DOWN |
| SPINK1 | 2.953446 | -1.96813 | 5.061349 | 1.45E-06 | 1.77E-05 | 5.02426 | UP |
| REN | -2.76051 | -3.39092 | -5.0618 | 1.44E-06 | 1.77E-05 | 5.020376 | DOWN |
| MYOM1 | -2.44877 | 0.672897 | -5.10291 | 1.21E-06 | 1.51E-05 | 4.999554 | DOWN |
| AQP9 | 3.050999 | 1.42442 | 5.034055 | 1.63E-06 | 1.95E-05 | 4.906655 | UP |
| CLDN8 | -3.32503 | 0.079908 | -5.08099 | 1.33E-06 | 1.64E-05 | 4.894939 | DOWN |
| NXPH4 | 3.584564 | 4.037489 | 5.022752 | 1.71E-06 | 2.04E-05 | 4.796832 | UP |
| VAX2 | 3.105587 | -0.8262 | 5.001344 | 1.88E-06 | 2.21E-05 | 4.780299 | UP |
| KRT13 | -4.7266 | 9.167113 | -5.11855 | 1.13E-06 | 1.42E-05 | 4.778636 | DOWN |
| NID1 | 2.521443 | 6.027582 | 5.085621 | 1.30E-06 | 1.61E-05 | 4.75736 | UP |
| MMP9 | 4.609919 | 5.812708 | 5.025901 | 1.69E-06 | 2.01E-05 | 4.751134 | UP |
| SFTA2 | -4.10912 | -3.17953 | -4.99352 | 1.94E-06 | 2.27E-05 | 4.711356 | DOWN |
| CFAP46 | -2.56857 | -1.09062 | -4.99198 | 1.95E-06 | 2.28E-05 | 4.673687 | DOWN |
| VIP | -2.75279 | -4.23843 | -4.97282 | 2.12E-06 | 2.45E-05 | 4.665298 | DOWN |
| CHST4 | -2.61252 | -1.7962 | -4.97905 | 2.07E-06 | 2.40E-05 | 4.651876 | DOWN |
| PCK1 | -3.15153 | -3.48177 | -4.9715 | 2.14E-06 | 2.47E-05 | 4.650399 | DOWN |
| CST9 | -2.50808 | -5.26395 | -4.96027 | 2.24E-06 | 2.58E-05 | 4.615737 | DOWN |
| C9orf135 | -3.10492 | -5.23852 | -4.95815 | 2.26E-06 | 2.60E-05 | 4.606847 | DOWN |
| NKX6-1 | 2.436348 | -0.90614 | 4.953586 | 2.31E-06 | 2.64E-05 | 4.588068 | UP |
| DIRC1 | 2.350763 | -4.20522 | 4.94912 | 2.35E-06 | 2.68E-05 | 4.570545 | UP |
| CILP2 | 3.027205 | 1.239334 | 4.927861 | 2.58E-06 | 2.91E-05 | 4.479667 | UP |
| ACER1 | -3.07422 | -1.52262 | -4.94275 | 2.42E-06 | 2.75E-05 | 4.475188 | DOWN |
| RNASE10 | 4.404105 | -0.70124 | 4.919146 | 2.67E-06 | 3.00E-05 | 4.450528 | UP |
| TREM1 | 3.333151 | 2.341416 | 4.919316 | 2.67E-06 | 3.00E-05 | 4.433158 | UP |
| THBS2 | 2.436927 | 6.685892 | 5.028737 | 1.67E-06 | 1.99E-05 | 4.430025 | UP |
| EMX1 | 3.019604 | -0.43849 | 4.896577 | 2.94E-06 | 3.27E-05 | 4.35974 | UP |
| CEACAM6 | -3.37914 | 6.148228 | -5.03426 | 1.63E-06 | 1.95E-05 | 4.352205 | DOWN |
| AC087651.1 | -2.4997 | -5.19717 | -4.88965 | 3.03E-06 | 3.36E-05 | 4.333781 | DOWN |
| EPHX3 | -2.57313 | 3.552 | -4.99865 | 1.90E-06 | 2.23E-05 | 4.244668 | DOWN |
| CNTFR | -2.67053 | -0.91394 | -4.88882 | 3.04E-06 | 3.36E-05 | 4.240023 | DOWN |
| COL10A1 | 5.997509 | 3.125274 | 4.860476 | 3.43E-06 | 3.75E-05 | 4.209064 | UP |
| PNPLA3 | 2.538945 | 0.004336 | 4.857604 | 3.47E-06 | 3.78E-05 | 4.203674 | UP |
| EMILIN1 | 2.4536 | 5.178407 | 4.924399 | 2.61E-06 | 2.95E-05 | 4.196833 | UP |
| ROBO2 | -2.69022 | 0.595167 | -4.9118 | 2.76E-06 | 3.09E-05 | 4.193315 | DOWN |
| AC055733.1 | 3.452572 | -2.9094 | 4.852378 | 3.55E-06 | 3.85E-05 | 4.185846 | UP |
| PTHLH | 3.074088 | 6.788389 | 4.949535 | 2.35E-06 | 2.68E-05 | 4.157019 | UP |
| KLK13 | -3.8768 | 4.886899 | -4.98284 | 2.03E-06 | 2.36E-05 | 4.138752 | DOWN |
| GNG8 | 2.509715 | -3.52019 | 4.826001 | 3.97E-06 | 4.23E-05 | 4.082173 | UP |
| FADS2 | 2.817662 | 5.732315 | 4.887635 | 3.06E-06 | 3.38E-05 | 4.014825 | UP |
| WDR49 | -3.047 | -1.60307 | -4.82708 | 3.95E-06 | 4.22E-05 | 4.012533 | DOWN |
| FOSB | -2.72711 | 5.421681 | -4.9518 | 2.32E-06 | 2.66E-05 | 3.997985 | DOWN |
| HTRA4 | 2.448909 | -2.49641 | 4.799622 | 4.44E-06 | 4.65E-05 | 3.979114 | UP |
| TCHHL1 | 3.406177 | -2.89628 | 4.798511 | 4.46E-06 | 4.67E-05 | 3.973773 | UP |
| CWH43 | -3.06177 | 1.269282 | -4.87995 | 3.16E-06 | 3.47E-05 | 3.956993 | DOWN |
| ECM1 | -2.51777 | 7.143794 | -4.93864 | 2.46E-06 | 2.80E-05 | 3.95436 | DOWN |
| PRR32 | -2.96289 | -5.27387 | -4.79302 | 4.57E-06 | 4.76E-05 | 3.952066 | DOWN |
| OCA2 | -2.49039 | -0.35006 | -4.82305 | 4.02E-06 | 4.28E-05 | 3.946575 | DOWN |
| TFAP2B | -3.19023 | -3.50891 | -4.78832 | 4.66E-06 | 4.84E-05 | 3.920413 | DOWN |
| KCNB1 | -2.55383 | -2.31582 | -4.78851 | 4.65E-06 | 4.84E-05 | 3.905473 | DOWN |
| NCCRP1 | -3.39621 | 5.445427 | -4.92812 | 2.57E-06 | 2.91E-05 | 3.905457 | DOWN |
| AQP3 | -3.09534 | 8.282595 | -4.91538 | 2.72E-06 | 3.05E-05 | 3.885028 | DOWN |
| NLRP7 | 3.622194 | -0.92159 | 4.76939 | 5.04E-06 | 5.20E-05 | 3.85943 | UP |
| TRPA1 | 2.451289 | -0.63629 | 4.760878 | 5.23E-06 | 5.36E-05 | 3.82486 | UP |
| HOXD12 | 2.437892 | -4.33284 | 4.759304 | 5.26E-06 | 5.38E-05 | 3.819708 | UP |
| SMR3B | -2.89736 | -5.33405 | -4.75092 | 5.45E-06 | 5.54E-05 | 3.78764 | DOWN |
| ALPK2 | 2.665499 | 0.677541 | 4.749503 | 5.48E-06 | 5.57E-05 | 3.776735 | UP |
| F2RL2 | 3.161364 | 2.924566 | 4.757356 | 5.30E-06 | 5.42E-05 | 3.761838 | UP |
| TMC5 | -2.55096 | 2.110378 | -4.82878 | 3.93E-06 | 4.19E-05 | 3.676734 | DOWN |
| CAPNS2 | -2.60067 | 3.382242 | -4.84768 | 3.62E-06 | 3.91E-05 | 3.634913 | DOWN |
| ADAM12 | 3.945236 | 4.530298 | 4.724523 | 6.09E-06 | 6.12E-05 | 3.582978 | UP |
| RGS4 | 2.445181 | 1.680202 | 4.704455 | 6.62E-06 | 6.58E-05 | 3.580794 | UP |
| GSTA1 | -3.61905 | 1.655963 | -4.80618 | 4.32E-06 | 4.54E-05 | 3.560936 | DOWN |
| SLCO1A2 | 3.978696 | 0.689058 | 4.679489 | 7.34E-06 | 7.19E-05 | 3.505931 | UP |
| C1orf87 | -3.06841 | -4.85424 | -4.6726 | 7.55E-06 | 7.36E-05 | 3.483572 | DOWN |
| CDH11 | 3.164897 | 5.142753 | 4.719744 | 6.21E-06 | 6.23E-05 | 3.452491 | UP |
| ARTN | 3.39442 | 3.298276 | 4.674462 | 7.49E-06 | 7.32E-05 | 3.432047 | UP |
| CLEC12B | 2.713674 | -3.30018 | 4.656546 | 8.07E-06 | 7.78E-05 | 3.422872 | UP |
| MATN3 | 2.683388 | 0.642898 | 4.647407 | 8.38E-06 | 8.01E-05 | 3.381331 | UP |
| SP9 | 3.239337 | -2.1733 | 4.64184 | 8.58E-06 | 8.17E-05 | 3.36696 | UP |
| PYGM | -2.84167 | -0.25613 | -4.6712 | 7.60E-06 | 7.39E-05 | 3.308415 | DOWN |
| IGSF10 | -2.40597 | 1.010658 | -4.69182 | 6.97E-06 | 6.89E-05 | 3.281454 | DOWN |
| HTR2C | 4.059818 | -2.15026 | 4.607803 | 9.87E-06 | 9.23E-05 | 3.235908 | UP |
| VAX1 | 3.367031 | -2.53592 | 4.603953 | 1.00E-05 | 9.34E-05 | 3.221722 | UP |
| CNFN | -3.28595 | 5.992579 | -4.75801 | 5.29E-06 | 5.41E-05 | 3.211871 | DOWN |
| LPO | -3.48175 | -1.64631 | -4.62539 | 9.18E-06 | 8.66E-05 | 3.197353 | DOWN |
| SLC24A2 | 2.552245 | -0.69393 | 4.592945 | 1.05E-05 | 9.69E-05 | 3.177386 | UP |
| DUSP9 | 2.980697 | 2.60061 | 4.603497 | 1.00E-05 | 9.35E-05 | 3.171501 | UP |
| PGLYRP2 | 2.649084 | -2.97574 | 4.589669 | 1.06E-05 | 9.80E-05 | 3.167638 | UP |
| TMEM158 | 2.486411 | 3.305704 | 4.607616 | 9.88E-06 | 9.23E-05 | 3.126774 | UP |
| ACAN | 2.801099 | 1.001888 | 4.574024 | 1.13E-05 | 0.000104 | 3.098731 | UP |
| IFI6 | 2.761679 | 6.964108 | 4.696929 | 6.83E-06 | 6.77E-05 | 3.072447 | UP |
| ZNF114 | 2.947147 | 0.990847 | 4.561837 | 1.19E-05 | 0.000108 | 3.053394 | UP |
| GPR176 | 3.09917 | 4.008602 | 4.592071 | 1.05E-05 | 9.72E-05 | 3.051458 | UP |
| FAM71F1 | 3.178495 | -3.11718 | 4.549377 | 1.25E-05 | 0.000113 | 3.013826 | UP |
| GREM1 | 2.94891 | 5.149845 | 4.613256 | 9.65E-06 | 9.06E-05 | 3.003345 | UP |
| B4GALNT1 | 3.615849 | 3.039426 | 4.543355 | 1.28E-05 | 0.000115 | 2.943341 | UP |
| KCNA1 | -2.39302 | -4.42471 | -4.51218 | 1.46E-05 | 0.000129 | 2.87305 | DOWN |
| GRIN2D | 3.660916 | 3.00419 | 4.520776 | 1.41E-05 | 0.000125 | 2.86066 | UP |
| NID2 | 2.78572 | 3.263577 | 4.527078 | 1.37E-05 | 0.000123 | 2.831465 | UP |
| PLEKHG4B | 3.268907 | 2.819128 | 4.497751 | 1.55E-05 | 0.000136 | 2.767639 | UP |
| TEX26 | -2.45732 | -5.25815 | -4.46184 | 1.79E-05 | 0.000154 | 2.68541 | DOWN |
| FSD2 | -2.35332 | -3.51563 | -4.46139 | 1.79E-05 | 0.000154 | 2.679898 | DOWN |
| KRT3 | -2.46038 | -1.17035 | -4.48198 | 1.65E-05 | 0.000143 | 2.676232 | DOWN |
| SERPINB10 | -2.73352 | -2.23447 | -4.46818 | 1.74E-05 | 0.000151 | 2.662731 | DOWN |
| HTRA3 | 2.677691 | 5.021892 | 4.52706 | 1.37E-05 | 0.000123 | 2.661174 | UP |
| TREM2 | 2.395037 | 2.668722 | 4.475995 | 1.69E-05 | 0.000146 | 2.660781 | UP |
| C1orf68 | 3.550292 | -2.56035 | 4.449502 | 1.88E-05 | 0.00016 | 2.639502 | UP |
| ONECUT3 | 2.646649 | -2.09431 | 4.438246 | 1.96E-05 | 0.000166 | 2.598607 | UP |
| LYPD1 | 3.070782 | 1.24225 | 4.43564 | 1.99E-05 | 0.000167 | 2.577302 | UP |
| PIK3C2G | -2.60886 | -1.13795 | -4.45134 | 1.86E-05 | 0.000159 | 2.547594 | DOWN |
| MS4A8 | -3.67468 | -3.0431 | -4.43764 | 1.97E-05 | 0.000166 | 2.546694 | DOWN |
| MSTN | -2.53461 | -4.29816 | -4.42453 | 2.08E-05 | 0.000174 | 2.545476 | DOWN |
| HP | -2.93597 | -1.30239 | -4.44341 | 1.92E-05 | 0.000163 | 2.509839 | DOWN |
| MB | -2.89905 | 1.725073 | -4.52874 | 1.36E-05 | 0.000122 | 2.504078 | DOWN |
| ADAMTS19 | -2.37786 | -4.71137 | -4.4065 | 2.23E-05 | 0.000185 | 2.479384 | DOWN |
| SCGB2A1 | -2.96037 | -3.58432 | -4.40042 | 2.29E-05 | 0.000189 | 2.445222 | DOWN |
| TRIM15 | 2.802628 | -3.2385 | 4.38871 | 2.39E-05 | 0.000196 | 2.414462 | UP |
| WNT2 | 3.404008 | 1.211311 | 4.390588 | 2.38E-05 | 0.000195 | 2.411552 | UP |
| DGKI | 2.390423 | 0.505005 | 4.388991 | 2.39E-05 | 0.000196 | 2.407294 | UP |
| MMP11 | 5.005099 | 5.544682 | 4.419934 | 2.11E-05 | 0.000177 | 2.405224 | UP |
| KRTAP4-1 | 2.78047 | -2.46075 | 4.385472 | 2.43E-05 | 0.000198 | 2.403462 | UP |
| SLC5A7 | -2.96844 | -4.76095 | -4.36659 | 2.62E-05 | 0.00021 | 2.331715 | DOWN |
| AMPD1 | -2.52672 | -1.42037 | -4.38817 | 2.40E-05 | 0.000196 | 2.331661 | DOWN |
| GSTA2 | -3.02311 | -4.07911 | -4.35201 | 2.77E-05 | 0.000221 | 2.274974 | DOWN |
| SLC1A6 | 3.416699 | -2.8022 | 4.343827 | 2.86E-05 | 0.000227 | 2.249332 | UP |
| ONECUT1 | 2.486439 | -3.69617 | 4.342493 | 2.88E-05 | 0.000228 | 2.24445 | UP |
| FOLR3 | 3.542869 | -1.25949 | 4.333112 | 2.99E-05 | 0.000235 | 2.210306 | UP |
| TRPV3 | 2.951346 | 2.824831 | 4.350883 | 2.78E-05 | 0.000221 | 2.209678 | UP |
| INHBA | 4.458542 | 5.305713 | 4.369636 | 2.58E-05 | 0.000208 | 2.197647 | UP |
| SPX | -2.3748 | -4.01194 | -4.31941 | 3.15E-05 | 0.000246 | 2.158791 | DOWN |
| EPHA6 | -2.49309 | -3.29559 | -4.31514 | 3.21E-05 | 0.000249 | 2.134643 | DOWN |
| SERPINB12 | -2.86202 | -0.85812 | -4.33376 | 2.98E-05 | 0.000234 | 2.07251 | DOWN |
| ADH7 | -3.3677 | 5.02817 | -4.4692 | 1.73E-05 | 0.00015 | 2.070485 | DOWN |
| NOTUM | 2.726691 | 0.869365 | 4.293588 | 3.49E-05 | 0.000268 | 2.056207 | UP |
| MMP12 | 3.812979 | 4.682516 | 4.324791 | 3.09E-05 | 0.000241 | 2.034574 | UP |
| HAPLN1 | 2.451078 | 0.638761 | 4.275266 | 3.75E-05 | 0.000284 | 1.990672 | UP |
| KRT82 | 2.533575 | -3.64576 | 4.271815 | 3.80E-05 | 0.000287 | 1.987514 | UP |
| LRRC15 | 4.898125 | 3.633203 | 4.280377 | 3.67E-05 | 0.000279 | 1.982141 | UP |
| APCDD1L | 3.087973 | 1.0412 | 4.272737 | 3.79E-05 | 0.000286 | 1.980901 | UP |
| APOBEC2 | -3.16191 | -2.48564 | -4.28612 | 3.59E-05 | 0.000275 | 1.980236 | DOWN |
| ISG15 | 2.665394 | 6.196176 | 4.39307 | 2.35E-05 | 0.000194 | 1.979258 | UP |
| ST8SIA2 | 2.366563 | -0.42168 | 4.26829 | 3.85E-05 | 0.00029 | 1.970856 | UP |
| DCSTAMP | 2.762356 | -2.70073 | 4.256343 | 4.04E-05 | 0.000302 | 1.932942 | UP |
| KRT16 | 2.377455 | 10.72012 | 4.432723 | 2.01E-05 | 0.000169 | 1.927785 | UP |
| TCN1 | -3.05815 | 2.541011 | -4.40136 | 2.28E-05 | 0.000188 | 1.916521 | DOWN |
| TNNT3 | -2.62041 | 2.43125 | -4.38254 | 2.45E-05 | 0.0002 | 1.880954 | DOWN |
| SYNDIG1 | 2.761655 | 0.254653 | 4.243175 | 4.25E-05 | 0.000316 | 1.877637 | UP |
| ERBB4 | -2.39664 | -2.01809 | -4.25166 | 4.11E-05 | 0.000307 | 1.864148 | DOWN |
| SEMA3E | -2.60982 | -1.90037 | -4.25362 | 4.08E-05 | 0.000305 | 1.857667 | DOWN |
| CEBPE | 2.418221 | -2.65597 | 4.225011 | 4.56E-05 | 0.000335 | 1.820606 | UP |
| CXCL5 | 3.538598 | 1.157929 | 4.222803 | 4.60E-05 | 0.000337 | 1.80093 | UP |
| CLDN17 | -3.47801 | -1.90704 | -4.24251 | 4.26E-05 | 0.000316 | 1.775288 | DOWN |
| EN1 | 4.865849 | 0.634414 | 4.21167 | 4.80E-05 | 0.000349 | 1.76511 | UP |
| CSF2 | 3.214957 | -0.79676 | 4.207001 | 4.89E-05 | 0.000354 | 1.753129 | UP |
| TFF3 | -2.60604 | 1.793889 | -4.32618 | 3.07E-05 | 0.00024 | 1.745375 | DOWN |
| KCNJ16 | -2.78706 | -2.94947 | -4.20945 | 4.84E-05 | 0.000351 | 1.735113 | DOWN |
| SCGB3A2 | -3.41069 | -2.77709 | -4.21385 | 4.76E-05 | 0.000346 | 1.721671 | DOWN |
| LAMP5 | 2.990924 | 1.646946 | 4.20118 | 5.00E-05 | 0.000361 | 1.711809 | UP |
| KIAA2022 | -2.4246 | -1.4489 | -4.21251 | 4.79E-05 | 0.000348 | 1.693537 | DOWN |
| ARHGAP36 | -2.47465 | -4.01679 | -4.18562 | 5.31E-05 | 0.00038 | 1.676594 | DOWN |
| H2BFWT | 2.438261 | -3.57682 | 4.184144 | 5.34E-05 | 0.000382 | 1.673544 | UP |
| PRAME | 6.564462 | 1.647376 | 4.183682 | 5.35E-05 | 0.000382 | 1.661761 | UP |
| THRSP | -2.37426 | -4.83364 | -4.17881 | 5.45E-05 | 0.000388 | 1.653827 | DOWN |
| KLHL33 | -2.41983 | -4.59442 | -4.17667 | 5.50E-05 | 0.000391 | 1.645796 | DOWN |
| NELL2 | 3.444344 | 5.274252 | 4.241421 | 4.28E-05 | 0.000317 | 1.63734 | UP |
| RIMS4 | -2.65301 | -2.38007 | -4.18693 | 5.28E-05 | 0.000379 | 1.637178 | DOWN |
| APOE | 2.659336 | 5.964819 | 4.285262 | 3.60E-05 | 0.000275 | 1.605901 | UP |
| FABP12 | -2.37258 | -4.43776 | -4.15691 | 5.93E-05 | 0.000416 | 1.575535 | DOWN |
| ONECUT2 | 2.646714 | -0.37851 | 4.154978 | 5.98E-05 | 0.000418 | 1.565466 | UP |
| HTR3A | -2.82077 | -0.9322 | -4.18751 | 5.27E-05 | 0.000378 | 1.548013 | DOWN |
| DRD1 | -2.45356 | -3.40883 | -4.14363 | 6.24E-05 | 0.000435 | 1.521468 | DOWN |
| EPO | 2.787697 | -1.77621 | 4.134161 | 6.47E-05 | 0.000448 | 1.497186 | UP |
| FIBCD1 | 4.855124 | 0.531078 | 4.131911 | 6.53E-05 | 0.000451 | 1.482033 | UP |
| COL8A1 | 3.196992 | 4.403553 | 4.175805 | 5.52E-05 | 0.000391 | 1.46174 | UP |
| PDZD7 | 2.609011 | -0.03492 | 4.125687 | 6.69E-05 | 0.00046 | 1.460288 | UP |
| TLX2 | 2.414295 | -2.17098 | 4.121666 | 6.79E-05 | 0.000466 | 1.453527 | UP |
| ADCY10 | 2.512273 | -1.75981 | 4.118123 | 6.88E-05 | 0.000471 | 1.440526 | UP |
| APOBEC4 | -2.53942 | -5.38465 | -4.11216 | 7.04E-05 | 0.00048 | 1.418893 | DOWN |
| FEZF1 | 3.535653 | -1.91518 | 4.090521 | 7.65E-05 | 0.000516 | 1.343891 | UP |
| EPGN | -2.43597 | 1.29604 | -4.17652 | 5.50E-05 | 0.000391 | 1.288927 | DOWN |
| APLN | 2.405844 | 2.509277 | 4.084264 | 7.83E-05 | 0.000527 | 1.236071 | UP |
| FOXD3 | 2.445515 | -1.66079 | 4.059437 | 8.60E-05 | 0.000572 | 1.23502 | UP |
| LHX1 | 3.673567 | -2.02687 | 4.058654 | 8.63E-05 | 0.000572 | 1.232539 | UP |
| RGS7BP | -2.36503 | -3.13859 | -4.05836 | 8.64E-05 | 0.000573 | 1.217534 | DOWN |
| NR2E1 | 3.227768 | -2.28394 | 4.04441 | 9.10E-05 | 0.000599 | 1.183469 | UP |
| PTPRN | 2.675663 | -0.84767 | 4.044753 | 9.09E-05 | 0.000598 | 1.181253 | UP |
| CDH19 | -2.69581 | -2.41153 | -4.05614 | 8.71E-05 | 0.000577 | 1.173842 | DOWN |
| KRT23 | -2.44098 | 4.216637 | -4.22266 | 4.60E-05 | 0.000337 | 1.154213 | DOWN |
| PLAC1 | 2.972831 | -0.54805 | 4.029335 | 9.64E-05 | 0.000627 | 1.126779 | UP |
| OR6C70 | 2.563566 | -4.03869 | 4.02624 | 9.75E-05 | 0.000633 | 1.117936 | UP |
| LCE1F | 3.398776 | -2.47419 | 4.01266 | 0.000103 | 0.000661 | 1.073159 | UP |
| HAP1 | 3.310536 | 2.410668 | 4.02189 | 9.91E-05 | 0.000642 | 1.059008 | UP |
| TGM1 | -2.93951 | 6.698233 | -4.19725 | 5.08E-05 | 0.000366 | 1.047878 | DOWN |
| TBL1Y | -3.05939 | -3.82648 | -4.00542 | 0.000105 | 0.000676 | 1.035748 | DOWN |
| APELA | 2.434847 | -2.03411 | 3.985344 | 0.000114 | 0.000722 | 0.979877 | UP |
| TNNI2 | -2.4 | 1.626012 | -4.10036 | 7.36E-05 | 0.000499 | 0.965136 | DOWN |
| RPS6KA6 | -2.67505 | -0.86275 | -4.01351 | 0.000102 | 0.000659 | 0.927645 | DOWN |
| CHIT1 | 3.978321 | 0.874756 | 3.96796 | 0.000121 | 0.000762 | 0.909108 | UP |
| HRASLS5 | -2.46698 | -2.31121 | -3.9781 | 0.000117 | 0.000739 | 0.907056 | DOWN |
| COL2A1 | 2.774922 | -0.45138 | 3.961506 | 0.000124 | 0.000778 | 0.892789 | UP |
| AGR3 | -3.13949 | -1.75518 | -3.99019 | 0.000112 | 0.000711 | 0.885701 | DOWN |
| HMGA2 | 3.595392 | 3.899335 | 3.991889 | 0.000111 | 0.000707 | 0.882299 | UP |
| SPRR3 | -4.71245 | 7.260559 | -4.12701 | 6.65E-05 | 0.000458 | 0.827476 | DOWN |
| SP8 | 2.94873 | -2.85278 | 3.935895 | 0.000137 | 0.000841 | 0.810635 | UP |
| IGFL3 | 4.216726 | -1.48137 | 3.918077 | 0.000146 | 0.00089 | 0.749229 | UP |
| ASB4 | -2.35541 | -4.08485 | -3.91104 | 0.00015 | 0.00091 | 0.723753 | DOWN |
| SERPINI2 | -2.6931 | -3.88673 | -3.91 | 0.00015 | 0.000913 | 0.717295 | DOWN |
| VCAN | 2.440683 | 6.237712 | 4.059333 | 8.61E-05 | 0.000572 | 0.706635 | UP |
| FOXL1 | 2.428423 | 0.69835 | 3.907711 | 0.000152 | 0.000918 | 0.701366 | UP |
| DLK1 | -3.25796 | -4.35593 | -3.90532 | 0.000153 | 0.000924 | 0.700996 | DOWN |
| TOX3 | -2.9708 | -1.96222 | -3.91933 | 0.000145 | 0.000888 | 0.663727 | DOWN |
| MSC | 2.607546 | 3.5168 | 3.936355 | 0.000136 | 0.00084 | 0.65666 | UP |
| NECAB2 | 2.534407 | -1.78185 | 3.878119 | 0.000169 | 0.001007 | 0.615624 | UP |
| TEX101 | -2.39741 | -1.98203 | -3.89125 | 0.000161 | 0.000966 | 0.597764 | DOWN |
| A2ML1 | -2.62726 | 7.223607 | -4.0716 | 8.22E-05 | 0.000549 | 0.593153 | DOWN |
| UGT2A1 | -2.56094 | -4.75486 | -3.86814 | 0.000175 | 0.001039 | 0.580138 | DOWN |
| ALOX15 | -2.77959 | 0.818523 | -3.9687 | 0.000121 | 0.000761 | 0.570891 | DOWN |
| KHDC1L | 3.758243 | -0.67887 | 3.860156 | 0.000181 | 0.001066 | 0.552303 | UP |
| HOXB8 | 2.428611 | -1.94216 | 3.857147 | 0.000182 | 0.001074 | 0.545597 | UP |
| OTOP3 | -2.64922 | 1.244052 | -3.97296 | 0.000119 | 0.000751 | 0.541107 | DOWN |
| COLEC11 | 2.405727 | -0.05175 | 3.854372 | 0.000184 | 0.001082 | 0.527791 | UP |
| DNAH17 | 3.77948 | 4.29031 | 3.877097 | 0.00017 | 0.001009 | 0.472292 | UP |
| ZNF280A | 3.076899 | -3.76557 | 3.834855 | 0.000198 | 0.001147 | 0.467964 | UP |
| PRR9 | 4.303623 | -0.59226 | 3.820723 | 0.000208 | 0.001195 | 0.420605 | UP |
| SGCG | -2.50769 | -1.82338 | -3.83813 | 0.000196 | 0.001136 | 0.404048 | DOWN |
| HS3ST4 | -2.97719 | -2.71892 | -3.82101 | 0.000208 | 0.001195 | 0.371923 | DOWN |
| PRB1 | -2.91082 | -4.95538 | -3.80548 | 0.00022 | 0.001254 | 0.371185 | DOWN |
| TNNC1 | -2.78604 | -0.03417 | -3.86914 | 0.000175 | 0.001036 | 0.337227 | DOWN |
| OTOP1 | -2.37993 | -4.65077 | -3.79008 | 0.000233 | 0.001313 | 0.32066 | DOWN |
| RBFOX1 | -2.72481 | -3.35556 | -3.79231 | 0.000231 | 0.001303 | 0.310362 | DOWN |
| TM4SF19 | 2.507845 | 1.423098 | 3.796107 | 0.000228 | 0.00129 | 0.309808 | UP |
| ABRA | -2.57183 | -4.49338 | -3.77654 | 0.000244 | 0.001368 | 0.275456 | DOWN |
| CASQ1 | -2.60418 | -1.56159 | -3.79683 | 0.000227 | 0.001288 | 0.244135 | DOWN |
| FAM81B | -2.62915 | -4.34405 | -3.7556 | 0.000264 | 0.00146 | 0.206357 | DOWN |
| CCDC38 | 2.548552 | -2.77161 | 3.750073 | 0.000269 | 0.001483 | 0.191877 | UP |
| MORN5 | -2.44127 | -4.6992 | -3.75084 | 0.000268 | 0.001481 | 0.191721 | DOWN |
| PNPLA1 | 2.581235 | -0.52675 | 3.747456 | 0.000271 | 0.001496 | 0.177089 | UP |
| GJB1 | -2.8014 | -2.70449 | -3.75051 | 0.000268 | 0.001482 | 0.144602 | DOWN |
| CA9 | 4.806446 | 3.070654 | 3.723457 | 0.000296 | 0.001606 | 0.075751 | UP |
| CDSN | 3.168361 | -1.52868 | 3.710502 | 0.00031 | 0.001665 | 0.062052 | UP |
| CXCL13 | 3.280928 | 3.312459 | 3.742361 | 0.000276 | 0.00152 | 0.054987 | UP |
| PCSK9 | 2.868618 | 5.130635 | 3.800679 | 0.000224 | 0.001273 | 0.033632 | UP |
| SPP1 | 4.236835 | 6.014005 | 3.779865 | 0.000242 | 0.001354 | 0.008447 | UP |
| ASB11 | -2.53196 | -4.85353 | -3.69322 | 0.000329 | 0.001752 | 0.004466 | DOWN |
| SLC5A8 | -3.15348 | -2.78268 | -3.70243 | 0.000319 | 0.001707 | -0.02442 | DOWN |
| MMP3 | 4.574575 | 4.377562 | 3.714553 | 0.000305 | 0.001646 | -0.02843 | UP |
| PNCK | 3.268908 | 2.283997 | 3.686321 | 0.000337 | 0.001789 | -0.06553 | UP |
| SUN3 | 2.500697 | -2.40029 | 3.668303 | 0.00036 | 0.001888 | -0.07307 | UP |
| SMR3A | -2.74704 | -5.0435 | -3.66178 | 0.000368 | 0.001921 | -0.09683 | DOWN |
| KREMEN2 | 3.012571 | 3.077547 | 3.684594 | 0.000339 | 0.001798 | -0.12684 | UP |
| VGLL2 | -2.63688 | -4.47078 | -3.64878 | 0.000385 | 0.002 | -0.13937 | DOWN |
| SYT14 | 2.650823 | -0.34565 | 3.644434 | 0.000391 | 0.002024 | -0.15736 | UP |
| FOXI3 | 3.399457 | -2.40908 | 3.629853 | 0.000412 | 0.002107 | -0.19707 | UP |
| MAGEA6 | 5.847524 | -0.49977 | 3.626539 | 0.000416 | 0.00213 | -0.21393 | UP |
| C6orf223 | 2.89443 | 0.492354 | 3.619955 | 0.000426 | 0.002173 | -0.24044 | UP |
| COL11A1 | 5.58715 | 2.959537 | 3.618277 | 0.000429 | 0.002184 | -0.25737 | UP |
| APOC1 | 2.885168 | 3.143509 | 3.648242 | 0.000386 | 0.002003 | -0.25873 | UP |
| IGFL2 | 3.42098 | 0.877469 | 3.592476 | 0.000469 | 0.002361 | -0.32934 | UP |
| MYOT | -2.47485 | -0.54839 | -3.63933 | 0.000398 | 0.002054 | -0.35007 | DOWN |
| COL22A1 | 3.349751 | 1.835257 | 3.586338 | 0.000479 | 0.002402 | -0.36271 | UP |
| ROS1 | 3.309531 | -0.84323 | 3.572042 | 0.000503 | 0.002506 | -0.3831 | UP |
| SOHLH1 | 4.150216 | -2.65056 | 3.566512 | 0.000513 | 0.002544 | -0.40081 | UP |
| KLHDC7B | 3.952577 | 3.138147 | 3.578739 | 0.000492 | 0.00246 | -0.42398 | UP |
| ULBP1 | 2.51218 | 0.551985 | 3.560705 | 0.000524 | 0.002583 | -0.42944 | UP |
| GP2 | -3.895 | -2.73292 | -3.57854 | 0.000492 | 0.002461 | -0.46802 | DOWN |
| KLK12 | -2.57834 | 2.861095 | -3.71242 | 0.000308 | 0.001656 | -0.55214 | DOWN |
| CHRNA9 | 2.536408 | -2.30357 | 3.514044 | 0.000615 | 0.002968 | -0.5604 | UP |
| ASPN | 2.716319 | 3.675291 | 3.572299 | 0.000503 | 0.002505 | -0.56227 | UP |
| ALDH3A1 | -3.05523 | 7.278107 | -3.69088 | 0.000332 | 0.001764 | -0.72092 | DOWN |
| NKX2-5 | 3.254494 | -1.46622 | 3.460075 | 0.000739 | 0.003454 | -0.72822 | UP |
| HOXC10 | 4.314307 | 0.840754 | 3.460913 | 0.000737 | 0.003449 | -0.73813 | UP |
| CACNA1B | 3.32903 | 0.991311 | 3.459825 | 0.000739 | 0.003455 | -0.74422 | UP |
| NLRP10 | 2.641222 | -2.39898 | 3.452946 | 0.000757 | 0.003525 | -0.74868 | UP |
| SERPINB3 | -2.61827 | 5.549038 | -3.6891 | 0.000334 | 0.001774 | -0.74983 | DOWN |
| FTCD | 2.609243 | -2.61457 | 3.443457 | 0.000781 | 0.003618 | -0.77774 | UP |
| FBXO40 | -2.50428 | -4.26378 | -3.4336 | 0.000808 | 0.00372 | -0.81195 | DOWN |
| SPDYC | 3.434076 | -2.78844 | 3.429354 | 0.00082 | 0.003763 | -0.82298 | UP |
| MAGEA3 | 5.671747 | -0.24964 | 3.430207 | 0.000817 | 0.003756 | -0.82577 | UP |
| LYPD8 | 3.04531 | -3.09281 | 3.427277 | 0.000825 | 0.003785 | -0.82902 | UP |
| STEAP1B | 2.608042 | 0.748914 | 3.427082 | 0.000826 | 0.003786 | -0.8446 | UP |
| LHX3 | 2.619903 | -3.77911 | 3.411042 | 0.000872 | 0.00395 | -0.87947 | UP |
| RHCG | -3.32384 | 7.202912 | -3.64132 | 0.000395 | 0.002043 | -0.88254 | DOWN |
| AIM2 | 2.948429 | 2.872828 | 3.421433 | 0.000842 | 0.00384 | -0.94899 | UP |
| TLX3 | 3.545566 | -2.81317 | 3.384227 | 0.000953 | 0.004274 | -0.96012 | UP |
| SEC14L4 | 2.689597 | -1.38446 | 3.379951 | 0.000967 | 0.004326 | -0.9723 | UP |
| KRTAP19-1 | 2.486366 | -4.42734 | 3.37048 | 0.000998 | 0.004439 | -1.00309 | UP |
| AC004813.1 | 2.429036 | -4.34913 | 3.365395 | 0.001015 | 0.004503 | -1.01825 | UP |
| EN2 | 2.499025 | -0.29552 | 3.358004 | 0.00104 | 0.004595 | -1.04522 | UP |
| HHATL | -2.94647 | -3.93381 | -3.33786 | 0.001112 | 0.004852 | -1.10807 | DOWN |
| CCKAR | -2.46616 | -3.33946 | -3.33517 | 0.001122 | 0.004888 | -1.12101 | DOWN |
| MYOZ1 | -2.37686 | -0.52297 | -3.38389 | 0.000954 | 0.004278 | -1.14952 | DOWN |
| CST1 | 4.415715 | 1.376522 | 3.318736 | 0.001184 | 0.005111 | -1.17123 | UP |
| SH2D5 | 2.894367 | 2.315086 | 3.335451 | 0.001121 | 0.004884 | -1.17464 | UP |
| RASGEF1A | 2.623112 | 2.45854 | 3.339746 | 0.001105 | 0.004827 | -1.18962 | UP |
| MIOX | 2.7725 | -2.48112 | 3.299956 | 0.001259 | 0.005386 | -1.20834 | UP |
| EREG | -2.36269 | 2.709993 | -3.48833 | 0.000671 | 0.0032 | -1.25202 | DOWN |
| PRDM13 | 2.38556 | -1.91739 | 3.260867 | 0.001431 | 0.005993 | -1.32354 | UP |
| LMX1B | 2.825806 | 0.336295 | 3.262784 | 0.001422 | 0.00596 | -1.33093 | UP |
| MUC5AC | -3.16612 | -0.54124 | -3.31634 | 0.001194 | 0.005148 | -1.41483 | DOWN |
| FNDC1 | 2.564281 | 4.000419 | 3.307334 | 0.001229 | 0.005279 | -1.44146 | UP |
| ALX1 | 2.575981 | -2.46621 | 3.188763 | 0.001805 | 0.007286 | -1.53128 | UP |
| NHLH2 | -2.52993 | -1.30385 | -3.23031 | 0.001579 | 0.006493 | -1.54754 | DOWN |
| LRRC26 | -2.36558 | -3.64016 | -3.16007 | 0.001978 | 0.007858 | -1.6211 | DOWN |
| MSI1 | 2.705662 | 0.870655 | 3.154271 | 0.002014 | 0.00798 | -1.65045 | UP |
| SNTN | -2.48523 | -3.87389 | -3.1436 | 0.002084 | 0.008187 | -1.66629 | DOWN |
| C20orf85 | -2.81255 | -4.2147 | -3.12348 | 0.00222 | 0.008642 | -1.72268 | DOWN |
| LHX9 | 2.475755 | -2.22744 | 3.107339 | 0.002336 | 0.009 | -1.76203 | UP |
| RAB3B | 3.528227 | 2.460857 | 3.10921 | 0.002322 | 0.00896 | -1.81616 | UP |
| MYBPC1 | -3.14058 | -0.28536 | -3.1874 | 0.001813 | 0.007309 | -1.8354 | DOWN |
| FETUB | -2.36452 | 1.127111 | -3.21307 | 0.00167 | 0.006819 | -1.86422 | DOWN |
| STRA8 | 2.774588 | -3.18395 | 3.062123 | 0.00269 | 0.010108 | -1.88947 | UP |
| BPIFB2 | -3.76098 | -0.99161 | -3.15713 | 0.001996 | 0.007917 | -1.90448 | DOWN |
| LAMA1 | 3.581233 | 2.473524 | 3.072275 | 0.002606 | 0.009853 | -1.91711 | UP |
| S100A8 | -2.48948 | 9.306533 | -3.29343 | 0.001287 | 0.005486 | -1.95818 | DOWN |
| SYT1 | 2.369961 | 1.687575 | 3.061106 | 0.002699 | 0.010134 | -1.96366 | UP |
| TSKS | 2.525817 | -0.42513 | 3.034076 | 0.002934 | 0.010856 | -1.97435 | UP |
| NRAP | -3.29346 | -1.56458 | -3.08611 | 0.002496 | 0.009506 | -1.99839 | DOWN |
| USP9Y | -2.54778 | 1.44885 | -3.18086 | 0.001851 | 0.007436 | -2.04078 | DOWN |
| MYH7 | -3.14047 | -0.98885 | -3.06876 | 0.002635 | 0.009933 | -2.08878 | DOWN |
| MAGEA9 | 3.081651 | -3.64822 | 2.98152 | 0.003447 | 0.012345 | -2.11334 | UP |
| C1orf110 | -2.36237 | 1.309262 | -3.13375 | 0.00215 | 0.008407 | -2.13826 | DOWN |
| BPIFA1 | -3.33579 | -1.83849 | -3.01965 | 0.003067 | 0.011245 | -2.16124 | DOWN |
| PAX3 | 2.679555 | -2.80475 | 2.94877 | 0.003807 | 0.013377 | -2.19683 | UP |
| COL4A6 | 3.026171 | 4.592926 | 3.049564 | 0.002797 | 0.010448 | -2.2094 | UP |
| GPR50 | 3.223403 | -2.14072 | 2.931911 | 0.004006 | 0.013991 | -2.2419 | UP |
| BCHE | 2.427291 | 0.357013 | 2.925921 | 0.004079 | 0.014197 | -2.27371 | UP |
| CSAG1 | 3.494992 | -2.35966 | 2.911969 | 0.004253 | 0.014684 | -2.29585 | UP |
| KLHL41 | -2.66216 | -0.96595 | -2.97773 | 0.003487 | 0.012462 | -2.30655 | DOWN |
| LTF | -3.50759 | 4.576369 | -3.18644 | 0.001818 | 0.007323 | -2.31775 | DOWN |
| CALB1 | 4.315815 | 1.506072 | 2.872383 | 0.004786 | 0.016155 | -2.42105 | UP |
| PTX4 | 2.621256 | -3.05687 | 2.859646 | 0.00497 | 0.016672 | -2.43283 | UP |
| FOXL2 | 2.608574 | -0.21683 | 2.862277 | 0.004931 | 0.01656 | -2.4364 | UP |
| GPR149 | 2.506822 | -3.55485 | 2.825949 | 0.005488 | 0.018073 | -2.52163 | UP |
| FAM216B | -2.36306 | -3.75076 | -2.82098 | 0.005568 | 0.018279 | -2.53948 | DOWN |
| BPIFB1 | -3.70141 | 2.919648 | -3.09008 | 0.002466 | 0.009412 | -2.53999 | DOWN |
| PPP1R3A | -2.42213 | -4.88434 | -2.80763 | 0.00579 | 0.018855 | -2.56992 | DOWN |
| FBN2 | 3.754819 | 4.176352 | 2.849089 | 0.005127 | 0.017104 | -2.64349 | UP |
| MAGEA12 | 3.571245 | -3.05041 | 2.751403 | 0.006814 | 0.021554 | -2.71447 | UP |
| MAGEA1 | 3.319266 | -3.2592 | 2.742914 | 0.006983 | 0.021991 | -2.73569 | UP |
| LGR5 | 2.615456 | 0.823376 | 2.740784 | 0.007025 | 0.022092 | -2.75918 | UP |
| POPDC3 | 2.58758 | 0.952368 | 2.735531 | 0.007132 | 0.022358 | -2.77583 | UP |
| DHRS2 | 2.433705 | 0.639024 | 2.723188 | 0.007388 | 0.022969 | -2.80132 | UP |
| SLCO1B3 | 2.752742 | -0.20255 | 2.716948 | 0.007521 | 0.023287 | -2.80739 | UP |
| MMP13 | 4.971103 | 3.862897 | 2.734761 | 0.007148 | 0.022389 | -2.82714 | UP |
| MYL1 | -3.29753 | -2.27463 | -2.72622 | 0.007324 | 0.022821 | -2.90181 | DOWN |
| HOXB13 | 2.754541 | -1.80784 | 2.666569 | 0.008674 | 0.02621 | -2.92131 | UP |
| BPIFC | 2.371606 | -1.00048 | 2.667998 | 0.008639 | 0.026127 | -2.92283 | UP |
| ADD2 | 2.509265 | 1.594232 | 2.691495 | 0.008085 | 0.024718 | -2.92332 | UP |
| MAGEA4 | 4.459139 | -0.43335 | 2.667265 | 0.008657 | 0.026172 | -2.92597 | UP |
| CNGB1 | 2.65337 | 1.572927 | 2.655678 | 0.008943 | 0.026865 | -2.9958 | UP |
| CXCL9 | 2.648844 | 4.653547 | 2.779288 | 0.006287 | 0.020134 | -3.00171 | UP |
| TDRD5 | 2.67955 | -0.24698 | 2.624257 | 0.009763 | 0.028832 | -3.03507 | UP |
| FGF19 | 2.676365 | -2.5373 | 2.61554 | 0.010002 | 0.029359 | -3.04467 | UP |
| CXCL10 | 2.467992 | 3.962147 | 2.702511 | 0.007836 | 0.024097 | -3.11845 | UP |
| NR5A1 | 3.434256 | -1.64321 | 2.577671 | 0.011104 | 0.031908 | -3.13647 | UP |
| SAGE1 | 2.730709 | -3.94992 | 2.569943 | 0.011342 | 0.032484 | -3.15908 | UP |
| CT45A10 | 2.535193 | -4.15551 | 2.542197 | 0.012234 | 0.034478 | -3.22467 | UP |
| MAGEB2 | 3.041264 | -3.14546 | 2.461285 | 0.015205 | 0.040998 | -3.40946 | UP |
| LMOD2 | -2.62202 | -3.07984 | -2.46128 | 0.015205 | 0.040998 | -3.44597 | DOWN |
| IL36G | 2.644726 | 4.207496 | 2.531364 | 0.012599 | 0.035268 | -3.53566 | UP |
| PIGR | -2.68129 | 3.663481 | -2.71414 | 0.007581 | 0.023438 | -3.58983 | DOWN |
| ACTA1 | -2.40343 | 0.69296 | -2.51922 | 0.01302 | 0.036241 | -3.66728 | DOWN |
| SCGB3A1 | -2.73473 | 1.79555 | -2.59406 | 0.010615 | 0.030766 | -3.69737 | DOWN |
| MAGEA11 | 3.108902 | -1.05218 | 2.30194 | 0.022989 | 0.057013 | -3.76395 | UP |
| MAGEA10 | 2.599807 | -3.68239 | 2.284325 | 0.024035 | 0.059087 | -3.80079 | UP |
| SPRR2G | 3.656723 | 3.081942 | 2.317296 | 0.02211 | 0.055229 | -3.82163 | UP |
| DMRTA2 | 2.537665 | -1.16678 | 2.272741 | 0.024745 | 0.060485 | -3.82658 | UP |
| ZIC1 | 2.464275 | -1.9543 | 2.266531 | 0.025133 | 0.061262 | -3.8356 | UP |
| DSC1 | 2.48075 | 1.637128 | 2.293007 | 0.023514 | 0.058033 | -3.84441 | UP |
| CASP14 | 3.442795 | 1.367736 | 2.236759 | 0.02707 | 0.065015 | -3.92371 | UP |
| PTH2R | 2.468095 | -0.62799 | 2.227777 | 0.02768 | 0.066201 | -3.92602 | UP |
| KRT75 | 2.80079 | 3.388887 | 2.280884 | 0.024244 | 0.059473 | -3.99204 | UP |
| MYH2 | -2.81236 | -1.11668 | -2.2831 | 0.024109 | 0.05921 | -4.00658 | DOWN |
| CCNA1 | 2.467438 | 1.697242 | 2.20669 | 0.029157 | 0.069062 | -4.03547 | UP |
| XIRP2 | -2.6453 | -2.37867 | -2.20558 | 0.029237 | 0.069203 | -4.04918 | DOWN |
| SPINK6 | 2.449931 | -0.50155 | 2.091348 | 0.038516 | 0.086004 | -4.20322 | UP |
| BRDT | 2.373291 | -3.37063 | 2.078851 | 0.039671 | 0.088024 | -4.21819 | UP |
| UCHL1 | 2.379728 | 4.013242 | 2.193363 | 0.030126 | 0.07097 | -4.31148 | UP |
| MUC5B | -2.54826 | 2.700746 | -2.32573 | 0.02164 | 0.054225 | -4.43476 | DOWN |
| MMP10 | 2.467776 | 4.766264 | 2.050624 | 0.042388 | 0.092929 | -4.7013 | UP |
