# Supplementary material for: PI3K/Akt signalling pathway-associated long noncoding RNA signature predicts the prognosis of laryngeal cancer patients
Source: Sci Rep. 2023 Sep 7;13:14764. doi: 10.1038/s41598-023-41927-3 (PMC10485045; doi:10.1038/s41598-023-41927-3)
Supplement: Supplementary file 6 — Supplementary Information 3. [file 41598_2023_41927_MOESM6_ESM.docx]

|  | logFC | AveExpr | t | P.Value | adj.P.Val | B | change |
| --- | --- | --- | --- | --- | --- | --- | --- |
| RP11-369C8.1 | -7.20333 | 1.811883 | -17.2073 | 2.09E-35 | 1.98E-31 | 69.66484 | DOWN |
| LINC00330 | 6.14981 | 3.093145 | 15.7071 | 6.69E-32 | 3.16E-28 | 61.7614 | UP |
| HCG22 | -6.39755 | 4.351865 | -13.7206 | 4.23E-27 | 1.33E-23 | 51.01407 | DOWN |
| SLC8A1-AS1 | -4.98631 | 4.022262 | -13.6549 | 6.13E-27 | 1.45E-23 | 50.58335 | DOWN |
| CYP4F35P | -5.13724 | 3.172814 | -13.4399 | 2.07E-26 | 3.92E-23 | 49.33775 | DOWN |
| IL12A-AS1 | -4.37879 | 3.8165 | -13.1086 | 1.37E-25 | 2.15E-22 | 47.50073 | DOWN |
| CTD-2376I4.2 | -3.05492 | 5.157172 | -12.7155 | 1.29E-24 | 1.54E-21 | 45.3275 | DOWN |
| RP11-351J23.2 | -5.58821 | 3.29033 | -12.7139 | 1.30E-24 | 1.54E-21 | 45.3213 | DOWN |
| CTD-2376I4.1 | -3.32347 | 3.841206 | -12.5995 | 2.50E-24 | 2.63E-21 | 44.59854 | DOWN |
| AC103563.9 | -5.79589 | 2.250807 | -12.4078 | 7.51E-24 | 7.10E-21 | 43.5763 | DOWN |
| RP11-237N19.3 | -4.6771 | 2.806358 | -12.1746 | 2.86E-23 | 2.46E-20 | 42.23298 | DOWN |
| RP11-757O6.1 | -4.65095 | 1.585586 | -11.9431 | 1.08E-22 | 8.54E-20 | 40.87119 | DOWN |
| RP11-677M14.3 | -2.91926 | 4.680748 | -11.4346 | 2.03E-21 | 1.38E-18 | 38.09465 | DOWN |
| RP11-739N20.2 | -4.27802 | 2.975953 | -11.433 | 2.04E-21 | 1.38E-18 | 38.05715 | DOWN |
| FALEC | -4.75106 | 4.239824 | -11.2291 | 6.62E-21 | 4.17E-18 | 36.96118 | DOWN |
| AC005532.5 | -4.58941 | 3.921362 | -11.0605 | 1.75E-20 | 1.03E-17 | 35.99965 | DOWN |
| LINC00551 | -4.27026 | 2.48736 | -10.9464 | 3.38E-20 | 1.88E-17 | 35.30162 | DOWN |
| AC103563.8 | -4.89468 | 2.389859 | -10.8639 | 5.43E-20 | 2.85E-17 | 34.86073 | DOWN |
| AC124944.5 | -4.75395 | 3.266438 | -10.7731 | 9.15E-20 | 4.56E-17 | 34.36704 | DOWN |
| RP11-875H7.5 | -4.8208 | 1.929485 | -10.6148 | 2.28E-19 | 1.08E-16 | 33.44824 | DOWN |
| RP11-7K24.3 | -3.17105 | 10.13906 | -10.3652 | 9.56E-19 | 4.30E-16 | 31.9449 | DOWN |
| PP14571 | -4.07783 | 6.749341 | -10.3288 | 1.18E-18 | 5.06E-16 | 31.8477 | DOWN |
| AC024592.9 | -4.13596 | 4.64156 | -10.3107 | 1.31E-18 | 5.37E-16 | 31.77326 | DOWN |
| CTC-480C2.1 | 4.985026 | 4.774614 | 10.11068 | 4.12E-18 | 1.62E-15 | 30.64749 | UP |
| RP11-351J23.1 | -6.09497 | 6.06873 | -10.1001 | 4.37E-18 | 1.65E-15 | 30.53 | DOWN |
| RP11-513G11.4 | -3.66458 | 3.855859 | -9.97951 | 8.72E-18 | 3.17E-15 | 29.90514 | DOWN |
| RP11-483P21.2 | -2.94858 | 5.881114 | -9.86634 | 1.67E-17 | 5.83E-15 | 29.27237 | DOWN |
| RP11-138J23.1 | 4.021157 | 4.132481 | 9.847259 | 1.86E-17 | 6.27E-15 | 29.16967 | UP |
| RP11-275I14.4 | -2.93503 | 5.530392 | -9.80726 | 2.33E-17 | 7.61E-15 | 28.94456 | DOWN |
| ZNF503-AS1 | -4.19994 | 6.00001 | -9.72541 | 3.72E-17 | 1.17E-14 | 28.46176 | DOWN |
| LINC01497 | -4.04522 | 1.276729 | -9.63536 | 6.22E-17 | 1.90E-14 | 27.93978 | DOWN |
| RP11-774O3.3 | -2.29426 | 8.102608 | -9.57632 | 8.71E-17 | 2.57E-14 | 27.60284 | DOWN |
| LA16c-380A1.1 | -3.2763 | 2.997804 | -9.5322 | 1.12E-16 | 3.21E-14 | 27.39182 | DOWN |
| AC097499.1 | -4.93297 | 1.886384 | -9.39695 | 2.41E-16 | 6.71E-14 | 26.65592 | DOWN |
| LINC00443 | -4.13931 | 1.366725 | -9.31017 | 3.94E-16 | 1.07E-13 | 26.15548 | DOWN |
| RP4-651E10.4 | -3.99318 | 2.442823 | -9.285 | 4.55E-16 | 1.19E-13 | 26.03062 | DOWN |
| RP11-141J13.3 | -3.68578 | 1.768719 | -9.27808 | 4.73E-16 | 1.21E-13 | 25.97169 | DOWN |
| RP11-281P23.1 | -3.9858 | 3.414493 | -9.2403 | 5.86E-16 | 1.46E-13 | 25.79277 | DOWN |
| RP11-93B14.10 | -3.15775 | 5.696332 | -9.2313 | 6.16E-16 | 1.49E-13 | 25.7277 | DOWN |
| RP11-60A8.1 | -3.92266 | 1.996735 | -9.19424 | 7.60E-16 | 1.75E-13 | 25.52548 | DOWN |
| CTD-2547H18.1 | -3.79438 | 3.006455 | -9.168 | 8.81E-16 | 1.98E-13 | 25.39066 | DOWN |
| RP11-407B7.1 | -3.40058 | 4.320303 | -9.1554 | 9.46E-16 | 2.08E-13 | 25.32343 | DOWN |
| RP11-855A2.5 | -3.27261 | 2.041542 | -9.03956 | 1.82E-15 | 3.91E-13 | 24.66034 | DOWN |
| AL161668.5 | -3.59006 | 2.398346 | -9.01997 | 2.03E-15 | 4.26E-13 | 24.56736 | DOWN |
| AC011286.1 | -3.55624 | 2.555386 | -8.96596 | 2.75E-15 | 5.65E-13 | 24.2753 | DOWN |
| RP1-232P20.1 | -4.17337 | 3.475051 | -8.94767 | 3.05E-15 | 6.13E-13 | 24.18135 | DOWN |
| CTD-2532K18.2 | 3.83261 | 3.989836 | 8.907152 | 3.83E-15 | 7.54E-13 | 23.94758 | UP |
| RP11-344B5.2 | -2.79262 | 5.071007 | -8.83329 | 5.79E-15 | 1.11E-12 | 23.54799 | DOWN |
| RP11-10H3.1 | -4.53074 | 1.628259 | -8.82577 | 6.04E-15 | 1.12E-12 | 23.51286 | DOWN |
| RP11-968A15.2 | 3.359499 | 4.603436 | 8.816796 | 6.35E-15 | 1.15E-12 | 23.44996 | UP |
| RP11-93B14.9 | -2.82436 | 7.957252 | -8.83063 | 5.88E-15 | 1.11E-12 | 23.44149 | DOWN |
| RP11-641D5.2 | -3.7383 | 3.549324 | -8.74408 | 9.54E-15 | 1.70E-12 | 23.06866 | DOWN |
| LINC00675 | -4.68562 | 4.444001 | -8.71623 | 1.11E-14 | 1.92E-12 | 22.89152 | DOWN |
| CTC-490G23.2 | -4.09497 | 6.443662 | -8.72256 | 1.08E-14 | 1.88E-12 | 22.87089 | DOWN |
| LINC01269 | -3.9996 | 6.039352 | -8.68585 | 1.32E-14 | 2.23E-12 | 22.68877 | DOWN |
| ABHD11-AS1 | -3.18002 | 5.66173 | -8.63109 | 1.79E-14 | 2.97E-12 | 22.4217 | DOWN |
| RP11-963H4.3 | -3.32473 | 1.691331 | -8.62281 | 1.88E-14 | 3.06E-12 | 22.39288 | DOWN |
| RP11-1103G16.1 | 5.755857 | 5.966431 | 8.60742 | 2.04E-14 | 3.28E-12 | 22.30489 | UP |
| RP11-575H3.1 | -4.00165 | 2.890245 | -8.58831 | 2.27E-14 | 3.58E-12 | 22.22347 | DOWN |
| CTA-280A3.2 | 2.960215 | 3.431296 | 8.583579 | 2.33E-14 | 3.62E-12 | 22.1848 | UP |
| RP11-146D12.2 | -3.26329 | 2.796016 | -8.56206 | 2.63E-14 | 4.01E-12 | 22.07941 | DOWN |
| CTD-2540L5.6 | -3.47927 | 4.106964 | -8.55184 | 2.78E-14 | 4.18E-12 | 22.01936 | DOWN |
| RP6-159A1.3 | -3.75031 | 2.62997 | -8.49353 | 3.85E-14 | 5.69E-12 | 21.71094 | DOWN |
| RP11-159F24.6 | 3.686181 | 7.136147 | 8.485407 | 4.03E-14 | 5.86E-12 | 21.63684 | UP |
| DLGAP1-AS5 | -4.48511 | 3.272347 | -8.45389 | 4.79E-14 | 6.87E-12 | 21.48891 | DOWN |
| RP11-10L7.1 | -3.01811 | 2.976719 | -8.45079 | 4.88E-14 | 6.88E-12 | 21.47917 | DOWN |
| LINC01170 | -3.62818 | 2.22134 | -8.42399 | 5.66E-14 | 7.75E-12 | 21.33457 | DOWN |
| LINC00898 | 4.229441 | 4.788472 | 8.416212 | 5.91E-14 | 7.93E-12 | 21.27972 | UP |
| RP11-552E20.4 | -2.78951 | 0.893593 | -8.41474 | 5.96E-14 | 7.93E-12 | 21.24823 | DOWN |
| RP11-235G24.3 | -4.02687 | 2.990537 | -8.38387 | 7.06E-14 | 9.28E-12 | 21.11776 | DOWN |
| GS1-120K12.4 | 2.947892 | 3.845728 | 8.361713 | 7.98E-14 | 1.03E-11 | 20.997 | UP |
| LINC01395 | -3.72199 | 3.956807 | -8.33209 | 9.40E-14 | 1.20E-11 | 20.83011 | DOWN |
| AL035610.1 | -4.50603 | 4.456911 | -8.29454 | 1.16E-13 | 1.46E-11 | 20.60011 | DOWN |
| RP3-428L16.1 | -3.82495 | 2.546238 | -8.17109 | 2.28E-13 | 2.84E-11 | 19.98046 | DOWN |
| LINC00900 | -3.04766 | 6.389057 | -8.16375 | 2.37E-13 | 2.92E-11 | 19.86333 | DOWN |
| DIO2-AS1 | -3.47807 | 1.430076 | -8.08449 | 3.67E-13 | 4.44E-11 | 19.51639 | DOWN |
| RP5-965F6.2 | -3.03259 | 2.105975 | -8.06849 | 4.00E-13 | 4.77E-11 | 19.43224 | DOWN |
| AC005162.5 | -3.67251 | 2.783436 | -8.06707 | 4.03E-13 | 4.77E-11 | 19.42603 | DOWN |
| RP11-108K14.12 | -3.82444 | 3.713875 | -8.05508 | 4.30E-13 | 5.03E-11 | 19.34782 | DOWN |
| RP11-18B16.2 | 2.476706 | 3.072189 | 7.99866 | 5.86E-13 | 6.75E-11 | 19.03928 | UP |
| EPB41L4A-AS2 | -2.2083 | 6.17929 | -7.96343 | 7.10E-13 | 8.09E-11 | 18.8219 | DOWN |
| RP11-469H8.6 | -4.63082 | 3.69938 | -7.93265 | 8.39E-13 | 9.45E-11 | 18.68191 | DOWN |
| RP11-474O21.5 | -2.88656 | 4.813419 | -7.92871 | 8.57E-13 | 9.54E-11 | 18.66593 | DOWN |
| AC004988.1 | 2.908165 | 3.528041 | 7.911078 | 9.43E-13 | 1.04E-10 | 18.5759 | UP |
| RP11-426C22.4 | 4.177143 | 5.810018 | 7.895856 | 1.02E-12 | 1.11E-10 | 18.51703 | UP |
| RP11-332J15.4 | 3.019777 | 3.43089 | 7.839073 | 1.39E-12 | 1.50E-10 | 18.18468 | UP |
| RP11-598F7.6 | 3.2252 | 4.87223 | 7.800057 | 1.72E-12 | 1.83E-10 | 18.01631 | UP |
| AP001347.6 | -2.63003 | 4.943635 | -7.70093 | 2.94E-12 | 3.09E-10 | 17.46384 | DOWN |
| AC012531.25 | 3.014135 | 3.265127 | 7.696338 | 3.01E-12 | 3.13E-10 | 17.42687 | UP |
| CLDN10-AS1 | -3.58496 | 1.904975 | -7.67782 | 3.33E-12 | 3.42E-10 | 17.38195 | DOWN |
| GS1-600G8.5 | 4.48749 | 5.115908 | 7.667318 | 3.52E-12 | 3.58E-10 | 17.29028 | UP |
| CTA-363E6.6 | -3.07387 | 1.981474 | -7.62856 | 4.34E-12 | 4.32E-10 | 17.12661 | DOWN |
| RP11-120K18.2 | 2.750041 | 3.477999 | 7.629986 | 4.31E-12 | 4.32E-10 | 17.09835 | UP |
| RP11-191L9.4 | 4.286358 | 5.961553 | 7.622754 | 4.48E-12 | 4.41E-10 | 17.09376 | UP |
| RP11-397A16.1 | 4.409225 | 6.443453 | 7.608704 | 4.83E-12 | 4.70E-10 | 17.01539 | UP |
| RP1-193H18.3 | -3.08264 | 1.124239 | -7.55324 | 6.50E-12 | 6.26E-10 | 16.73453 | DOWN |
| MLLT4-AS1 | -2.18395 | 5.779634 | -7.54806 | 6.68E-12 | 6.31E-10 | 16.64611 | DOWN |
| RP11-276H7.3 | -2.59179 | 4.255524 | -7.53727 | 7.07E-12 | 6.62E-10 | 16.63067 | DOWN |
| AC011288.2 | 5.089722 | 6.271803 | 7.505162 | 8.40E-12 | 7.78E-10 | 16.48536 | UP |
| RP11-440I14.2 | -3.24016 | 1.957132 | -7.48191 | 9.51E-12 | 8.69E-10 | 16.36772 | DOWN |
| TTC39A-AS1 | -2.9696 | 2.986314 | -7.47115 | 1.01E-11 | 9.07E-10 | 16.30667 | DOWN |
| PLBD1-AS1 | -2.61492 | 6.409557 | -7.48095 | 9.55E-12 | 8.69E-10 | 16.25356 | DOWN |
| CTD-2540L5.5 | -3.03295 | 1.236493 | -7.39982 | 1.47E-11 | 1.29E-09 | 15.94669 | DOWN |
| CTD-3179P9.1 | -3.14524 | 1.884436 | -7.39818 | 1.48E-11 | 1.29E-09 | 15.93841 | DOWN |
| RP11-25I15.3 | 3.166028 | 3.735629 | 7.405278 | 1.43E-11 | 1.26E-09 | 15.91616 | UP |
| RP11-159F24.5 | 3.292052 | 5.64546 | 7.369341 | 1.73E-11 | 1.47E-09 | 15.78615 | UP |
| RP11-1123I8.1 | -2.89484 | 1.305612 | -7.33614 | 2.06E-11 | 1.73E-09 | 15.62132 | DOWN |
| TDRG1 | -3.53382 | 2.393886 | -7.33598 | 2.06E-11 | 1.73E-09 | 15.61158 | DOWN |
| RP11-989E6.3 | -2.78726 | 1.002539 | -7.33018 | 2.13E-11 | 1.76E-09 | 15.58959 | DOWN |
| TMEM220-AS1 | -2.18361 | 4.920053 | -7.32917 | 2.14E-11 | 1.76E-09 | 15.54287 | DOWN |
| RP11-90L20.2 | 2.626688 | 3.511486 | 7.299333 | 2.50E-11 | 2.04E-09 | 15.4002 | UP |
| RP11-281P23.2 | -3.11408 | 2.355491 | -7.22731 | 3.66E-11 | 2.96E-09 | 15.06301 | DOWN |
| LMO7DN-IT1 | -2.6935 | 1.18964 | -7.21677 | 3.87E-11 | 3.10E-09 | 15.01448 | DOWN |
| HOXC-AS2 | 3.635949 | 5.934145 | 7.188103 | 4.50E-11 | 3.55E-09 | 14.86828 | UP |
| AC005062.2 | -2.75803 | 3.483993 | -7.16254 | 5.14E-11 | 3.95E-09 | 14.72038 | DOWN |
| CTD-2023M8.1 | 2.457809 | 3.27027 | 7.167191 | 5.02E-11 | 3.92E-09 | 14.70712 | UP |
| HOTAIR | 4.059322 | 4.723804 | 7.165746 | 5.06E-11 | 3.92E-09 | 14.69225 | UP |
| RP11-442J21.2 | -2.88407 | 2.211284 | -7.14031 | 5.78E-11 | 4.40E-09 | 14.62495 | DOWN |
| C15orf59-AS1 | -3.45352 | 2.974712 | -7.11624 | 6.55E-11 | 4.92E-09 | 14.48234 | DOWN |
| AC144831.3 | -2.32519 | 5.772328 | -7.11801 | 6.49E-11 | 4.91E-09 | 14.42339 | DOWN |
| AC118754.4 | -3.0758 | 2.177388 | -7.0983 | 7.20E-11 | 5.36E-09 | 14.41079 | DOWN |
| CTD-2034I4.2 | 2.201108 | 2.867094 | 7.082698 | 7.81E-11 | 5.72E-09 | 14.26989 | UP |
| RP11-80H8.4 | 2.389595 | 2.982817 | 7.078574 | 7.98E-11 | 5.80E-09 | 14.25012 | UP |
| CTD-2280E9.1 | 2.959303 | 4.326633 | 7.064049 | 8.60E-11 | 6.16E-09 | 14.23379 | UP |
| AC144831.1 | -2.51971 | 7.511825 | -7.09623 | 7.27E-11 | 5.37E-09 | 14.21516 | DOWN |
| LINC01100 | -2.30297 | 0.756308 | -7.02484 | 1.06E-10 | 7.50E-09 | 14.04929 | DOWN |
| RAET1E-AS1 | -2.83415 | 2.398049 | -7.016 | 1.10E-10 | 7.80E-09 | 13.99753 | DOWN |
| DLX2-AS1 | 2.757228 | 3.35184 | 7.007448 | 1.15E-10 | 8.09E-09 | 13.88438 | UP |
| RP11-70F11.8 | -3.05911 | 1.289713 | -6.96326 | 1.45E-10 | 9.76E-09 | 13.74068 | DOWN |
| CTC-276P9.4 | -2.8321 | 4.737089 | -6.97552 | 1.36E-10 | 9.34E-09 | 13.7276 | DOWN |
| RP11-465L10.10 | 3.162045 | 4.631779 | 6.961475 | 1.47E-10 | 9.76E-09 | 13.72275 | UP |
| LINC01587 | -2.50763 | 3.394861 | -6.9622 | 1.46E-10 | 9.76E-09 | 13.71602 | DOWN |
| RP11-123K19.1 | 2.58992 | 2.661399 | 6.975447 | 1.36E-10 | 9.34E-09 | 13.70507 | UP |
| RP11-308D13.3 | -3.20064 | 4.883946 | -6.93428 | 1.69E-10 | 1.12E-08 | 13.50184 | DOWN |
| RP11-678G14.3 | -3.36726 | 2.06798 | -6.91593 | 1.86E-10 | 1.22E-08 | 13.49522 | DOWN |
| AC112721.2 | 2.713721 | 3.115859 | 6.884337 | 2.18E-10 | 1.41E-08 | 13.25321 | UP |
| RP11-626E13.1 | -2.78671 | 1.277 | -6.85263 | 2.57E-10 | 1.61E-08 | 13.19254 | DOWN |
| AC093159.1 | -3.15989 | 4.108344 | -6.85511 | 2.54E-10 | 1.60E-08 | 13.13982 | DOWN |
| MEIS1-AS3 | -2.70761 | 2.058323 | -6.84107 | 2.73E-10 | 1.70E-08 | 13.13203 | DOWN |
| CTD-2091N23.1 | 2.274614 | 2.588223 | 6.855336 | 2.54E-10 | 1.60E-08 | 13.10481 | UP |
| AC079630.2 | -3.35124 | 1.303366 | -6.82507 | 2.96E-10 | 1.82E-08 | 13.05128 | DOWN |
| RP11-499F3.2 | 3.819778 | 5.328981 | 6.823932 | 2.98E-10 | 1.82E-08 | 13.04594 | UP |
| ALDH1L1-AS2 | -2.98869 | 2.240372 | -6.79702 | 3.42E-10 | 2.05E-08 | 12.90721 | DOWN |
| AP000439.3 | -3.21374 | 2.17458 | -6.79163 | 3.52E-10 | 2.08E-08 | 12.87735 | DOWN |
| RP11-25H12.1 | 2.742325 | 3.549265 | 6.761918 | 4.09E-10 | 2.38E-08 | 12.67928 | UP |
| RP1-122P22.4 | -3.08879 | 6.736933 | -6.78667 | 3.61E-10 | 2.12E-08 | 12.66648 | DOWN |
| RP11-350J20.12 | -3.22065 | 5.699429 | -6.71732 | 5.14E-10 | 2.93E-08 | 12.37493 | DOWN |
| CTC-297N7.9 | -2.23706 | 3.13568 | -6.68486 | 6.07E-10 | 3.41E-08 | 12.35173 | DOWN |
| STXBP5-AS1 | -2.19323 | 6.649827 | -6.6685 | 6.59E-10 | 3.69E-08 | 12.12185 | DOWN |
| RP1-8B1.4 | -3.01818 | 1.929982 | -6.62929 | 8.04E-10 | 4.45E-08 | 12.08872 | DOWN |
| RP11-408E5.5 | 3.062756 | 3.282769 | 6.637638 | 7.71E-10 | 4.29E-08 | 12.01829 | UP |
| AC093850.2 | 4.229543 | 4.89866 | 6.597381 | 9.45E-10 | 5.14E-08 | 11.85297 | UP |
| AC079630.4 | -2.3984 | 3.941443 | -6.52696 | 1.35E-09 | 7.21E-08 | 11.54942 | DOWN |
| RP1-127D3.4 | -3.10256 | 2.02227 | -6.51339 | 1.44E-09 | 7.67E-08 | 11.52052 | DOWN |
| RP11-250B2.3 | -2.17367 | 4.283862 | -6.49244 | 1.60E-09 | 8.43E-08 | 11.37379 | DOWN |
| GACAT2 | 2.44062 | 2.8166 | 6.500075 | 1.54E-09 | 8.16E-08 | 11.35058 | UP |
| RP11-297P16.3 | 3.301865 | 3.287272 | 6.489598 | 1.63E-09 | 8.50E-08 | 11.28685 | UP |
| AC007750.5 | 2.845528 | 4.351615 | 6.452936 | 1.96E-09 | 1.00E-07 | 11.23318 | UP |
| RP11-149I23.3 | -2.66151 | 2.133322 | -6.44946 | 1.99E-09 | 1.01E-07 | 11.21891 | DOWN |
| LL22NC03-N64E9.1 | 2.356991 | 2.772345 | 6.4645 | 1.85E-09 | 9.54E-08 | 11.18049 | UP |
| FLG-AS1 | -2.65169 | 6.799314 | -6.47696 | 1.73E-09 | 9.01E-08 | 11.14877 | DOWN |
| HOXC-AS3 | 2.599307 | 3.036732 | 6.457283 | 1.91E-09 | 9.83E-08 | 11.14461 | UP |
| TMEM51-AS1 | -2.25955 | 6.7058 | -6.44463 | 2.04E-09 | 1.03E-07 | 11.01365 | DOWN |
| LINC01028 | -3.00291 | 1.108691 | -6.40251 | 2.52E-09 | 1.23E-07 | 11.00081 | DOWN |
| RP11-239E10.3 | -3.17407 | 2.79163 | -6.4044 | 2.49E-09 | 1.23E-07 | 10.97157 | DOWN |
| PART1 | -3.37346 | 7.474654 | -6.45129 | 1.97E-09 | 1.00E-07 | 10.94878 | DOWN |
| LINC00689 | -2.51455 | 2.72414 | -6.37725 | 2.85E-09 | 1.39E-07 | 10.86423 | DOWN |
| CTC-215O4.4 | -2.21246 | 2.34468 | -6.35309 | 3.22E-09 | 1.56E-07 | 10.76117 | DOWN |
| RP11-594N15.3 | -3.00214 | 4.01561 | -6.34664 | 3.32E-09 | 1.60E-07 | 10.65373 | DOWN |
| RP11-800A3.4 | -2.19464 | 11.00282 | -6.40575 | 2.48E-09 | 1.23E-07 | 10.53526 | DOWN |
| RP3-473B4.3 | -2.68594 | 1.794445 | -6.30289 | 4.13E-09 | 1.94E-07 | 10.52041 | DOWN |
| LINC00922 | 2.502676 | 2.721235 | 6.324214 | 3.71E-09 | 1.76E-07 | 10.49173 | UP |
| AC002511.3 | -3.02394 | 3.680929 | -6.3039 | 4.11E-09 | 1.94E-07 | 10.46147 | DOWN |
| RP11-964E11.2 | -3.21612 | 6.274685 | -6.33333 | 3.55E-09 | 1.70E-07 | 10.45718 | DOWN |
| RP6-114E22.1 | 3.562502 | 4.498747 | 6.267089 | 4.93E-09 | 2.28E-07 | 10.28607 | UP |
| RP11-797H7.5 | -2.49499 | 2.764436 | -6.24093 | 5.60E-09 | 2.57E-07 | 10.21215 | DOWN |
| RP11-863K10.2 | -3.02079 | 1.482714 | -6.22088 | 6.19E-09 | 2.79E-07 | 10.13155 | DOWN |
| C5orf66-AS1 | -3.20603 | 7.153145 | -6.26918 | 4.88E-09 | 2.27E-07 | 10.09279 | DOWN |
| AC007392.3 | -2.21726 | 2.517493 | -6.19589 | 6.99E-09 | 3.13E-07 | 10.01238 | DOWN |
| RP11-109M17.2 | 4.058123 | 4.390382 | 6.222814 | 6.13E-09 | 2.77E-07 | 9.999769 | UP |
| RP11-742B18.1 | 5.062988 | 6.831348 | 6.172873 | 7.83E-09 | 3.44E-07 | 9.914306 | UP |
| RP4-663N10.1 | -2.71943 | 1.416072 | -6.15376 | 8.60E-09 | 3.75E-07 | 9.822523 | DOWN |
| RP11-215P8.4 | 4.014322 | 4.493476 | 6.180029 | 7.56E-09 | 3.36E-07 | 9.805305 | UP |
| RP11-473M20.5 | -2.28306 | 7.092489 | -6.18405 | 7.41E-09 | 3.31E-07 | 9.733105 | DOWN |
| HOXC-AS1 | 2.899387 | 4.327521 | 6.132533 | 9.54E-09 | 4.12E-07 | 9.681021 | UP |
| CTD-2619J13.13 | -2.5085 | 7.852036 | -6.17282 | 7.83E-09 | 3.44E-07 | 9.620601 | DOWN |
| RP11-519M16.1 | 2.870519 | 3.194153 | 6.140594 | 9.17E-09 | 3.98E-07 | 9.619319 | UP |
| CTB-113D17.1 | -2.66757 | 3.494389 | -6.09347 | 1.15E-08 | 4.85E-07 | 9.485393 | DOWN |
| RP11-94A24.1 | -2.67999 | 5.143436 | -6.11092 | 1.06E-08 | 4.56E-07 | 9.484832 | DOWN |
| CTD-2314G24.2 | -2.96421 | 4.594669 | -6.10554 | 1.09E-08 | 4.65E-07 | 9.477526 | DOWN |
| ADARB2-AS1 | -3.05493 | 2.539067 | -6.08298 | 1.21E-08 | 5.08E-07 | 9.457888 | DOWN |
| RP11-528A4.2 | -3.13824 | 2.785133 | -6.08098 | 1.23E-08 | 5.11E-07 | 9.43699 | DOWN |
| RP11-37C7.3 | 2.429523 | 4.341856 | 6.057311 | 1.38E-08 | 5.68E-07 | 9.37651 | UP |
| LL22NC03-N14H11.1 | 2.771134 | 3.946222 | 6.064959 | 1.33E-08 | 5.50E-07 | 9.335546 | UP |
| RP11-219B17.3 | -2.30483 | 2.97642 | -6.03768 | 1.51E-08 | 6.19E-07 | 9.255968 | DOWN |
| RP11-91K9.1 | 3.067534 | 3.863621 | 6.053063 | 1.40E-08 | 5.77E-07 | 9.242537 | UP |
| RP11-760H22.2 | -2.20691 | 6.262446 | -6.03131 | 1.56E-08 | 6.36E-07 | 9.064567 | DOWN |
| LINC01050 | 2.461313 | 3.132875 | 6.000199 | 1.81E-08 | 7.15E-07 | 8.997685 | UP |
| RBPMS-AS1 | -2.41767 | 5.213002 | -6.00005 | 1.81E-08 | 7.15E-07 | 8.969363 | DOWN |
| RP11-44B19.1 | -2.58184 | 2.513887 | -5.97283 | 2.07E-08 | 8.05E-07 | 8.959893 | DOWN |
| RP11-1C8.7 | 2.600029 | 3.298898 | 5.989201 | 1.91E-08 | 7.50E-07 | 8.926664 | UP |
| RP11-535A19.1 | -2.70857 | 2.772716 | -5.95884 | 2.21E-08 | 8.51E-07 | 8.883421 | DOWN |
| CTA-520D8.2 | 4.050011 | 5.144968 | 5.943421 | 2.38E-08 | 9.02E-07 | 8.785758 | UP |
| KB-1562D12.1 | -2.7985 | 5.790106 | -5.9661 | 2.14E-08 | 8.28E-07 | 8.761343 | DOWN |
| RP11-54O7.3 | -2.18126 | 5.629283 | -5.9479 | 2.33E-08 | 8.86E-07 | 8.712156 | DOWN |
| LINC00908 | -2.32585 | 4.260917 | -5.91608 | 2.72E-08 | 1.02E-06 | 8.635996 | DOWN |
| RP13-580F15.2 | -2.21056 | 4.190342 | -5.86715 | 3.43E-08 | 1.27E-06 | 8.417335 | DOWN |
| RP11-128M1.1 | -2.40189 | 5.112387 | -5.87816 | 3.26E-08 | 1.21E-06 | 8.410536 | DOWN |
| AC006026.13 | -2.50748 | 2.614098 | -5.85161 | 3.70E-08 | 1.36E-06 | 8.403746 | DOWN |
| RP11-234K24.6 | -2.2217 | 3.79314 | -5.85027 | 3.72E-08 | 1.36E-06 | 8.358873 | DOWN |
| CTD-2066L21.2 | 2.738199 | 3.095833 | 5.847523 | 3.77E-08 | 1.37E-06 | 8.247842 | UP |
| RP11-85B7.2 | -2.37553 | 3.890215 | -5.81413 | 4.42E-08 | 1.59E-06 | 8.181239 | DOWN |
| RP11-734K21.2 | -2.56168 | 4.626688 | -5.80654 | 4.58E-08 | 1.64E-06 | 8.100999 | DOWN |
| RP11-544M22.1 | 2.226628 | 2.447936 | 5.801838 | 4.69E-08 | 1.67E-06 | 8.03362 | UP |
| RP11-843B15.4 | 2.234276 | 3.728587 | 5.767606 | 5.51E-08 | 1.95E-06 | 8.023633 | UP |
| AC107072.2 | -2.66659 | 1.360258 | -5.75509 | 5.85E-08 | 2.06E-06 | 7.98824 | DOWN |
| RP5-999L4.2 | -2.18978 | 2.67065 | -5.75413 | 5.87E-08 | 2.06E-06 | 7.968237 | DOWN |
| MIR503HG | 3.297485 | 5.736189 | 5.739637 | 6.29E-08 | 2.19E-06 | 7.930879 | UP |
| AC107218.3 | -2.46886 | 1.059563 | -5.70845 | 7.28E-08 | 2.52E-06 | 7.787096 | DOWN |
| RP11-338K13.1 | -2.29147 | 2.346989 | -5.7075 | 7.31E-08 | 2.52E-06 | 7.764557 | DOWN |
| RP11-357D18.1 | -2.49677 | 1.032417 | -5.69167 | 7.88E-08 | 2.69E-06 | 7.712373 | DOWN |
| RP11-598F7.5 | 2.739228 | 5.301026 | 5.687375 | 8.04E-08 | 2.73E-06 | 7.697448 | UP |
| LINC01234 | 5.578161 | 6.186736 | 5.685936 | 8.09E-08 | 2.74E-06 | 7.559701 | UP |
| FIRRE | 4.035821 | 5.698125 | 5.651208 | 9.52E-08 | 3.17E-06 | 7.516256 | UP |
| RP11-664D7.4 | -3.08864 | 2.038584 | -5.63816 | 1.01E-07 | 3.31E-06 | 7.439157 | DOWN |
| RP11-318C2.1 | -2.189 | 1.590528 | -5.62985 | 1.05E-07 | 3.41E-06 | 7.4344 | DOWN |
| RNF144A-AS1 | 3.498623 | 6.981753 | 5.632294 | 1.04E-07 | 3.38E-06 | 7.434365 | UP |
| RP11-311F12.2 | -3.20309 | 3.975472 | -5.64877 | 9.63E-08 | 3.20E-06 | 7.391164 | DOWN |
| RP11-141M1.3 | -2.25379 | 2.685254 | -5.61378 | 1.13E-07 | 3.65E-06 | 7.335853 | DOWN |
| RP11-475O23.3 | -2.47341 | 1.540404 | -5.60788 | 1.17E-07 | 3.72E-06 | 7.331354 | DOWN |
| RP11-88H10.2 | -2.8237 | 1.480736 | -5.60943 | 1.16E-07 | 3.71E-06 | 7.331303 | DOWN |
| RP5-907D15.4 | 2.786476 | 3.800388 | 5.604858 | 1.18E-07 | 3.76E-06 | 7.239734 | UP |
| FAM225B | 2.482647 | 3.769011 | 5.593602 | 1.25E-07 | 3.91E-06 | 7.217459 | UP |
| RP11-559N14.5 | 2.852672 | 5.850529 | 5.57936 | 1.33E-07 | 4.13E-06 | 7.213656 | UP |
| RP11-789C1.1 | -2.7416 | 1.259335 | -5.57827 | 1.34E-07 | 4.13E-06 | 7.200095 | DOWN |
| RP11-353N14.4 | -2.33473 | 3.136631 | -5.55826 | 1.47E-07 | 4.43E-06 | 7.065567 | DOWN |
| RP4-541C22.5 | -2.33453 | 4.281494 | -5.57054 | 1.39E-07 | 4.24E-06 | 7.063799 | DOWN |
| LL22NC03-75H12.2 | -3.0691 | 4.036205 | -5.56781 | 1.40E-07 | 4.27E-06 | 7.02997 | DOWN |
| ZFY-AS1 | -3.01685 | 2.967549 | -5.53239 | 1.65E-07 | 4.94E-06 | 6.933445 | DOWN |
| RP5-1120P11.1 | 3.305311 | 6.387747 | 5.513775 | 1.80E-07 | 5.28E-06 | 6.921117 | UP |
| DUXAP8 | 4.255734 | 8.384518 | 5.522609 | 1.73E-07 | 5.11E-06 | 6.918911 | UP |
| DSG1-AS1 | -2.54474 | 6.987593 | -5.57884 | 1.33E-07 | 4.13E-06 | 6.916752 | DOWN |
| MRVI1-AS1 | -2.3294 | 4.094835 | -5.53215 | 1.66E-07 | 4.94E-06 | 6.902214 | DOWN |
| AC005281.2 | -2.83327 | 1.835187 | -5.51172 | 1.82E-07 | 5.31E-06 | 6.890294 | DOWN |
| CTD-2171N6.1 | 3.966891 | 5.577985 | 5.495101 | 1.96E-07 | 5.66E-06 | 6.81649 | UP |
| RP11-32K4.1 | 2.573543 | 2.630584 | 5.516386 | 1.78E-07 | 5.23E-06 | 6.74021 | UP |
| RP4-755D9.1 | -2.99912 | 5.872771 | -5.5239 | 1.72E-07 | 5.10E-06 | 6.721881 | DOWN |
| HOXB-AS4 | 3.223684 | 3.764806 | 5.463624 | 2.27E-07 | 6.43E-06 | 6.521317 | UP |
| RP11-626H12.2 | 2.798278 | 4.757844 | 5.422375 | 2.74E-07 | 7.69E-06 | 6.519652 | UP |
| RP11-417E7.2 | 2.49032 | 3.846449 | 5.413831 | 2.85E-07 | 7.98E-06 | 6.417923 | UP |
| RP11-12A2.3 | -2.38193 | 1.599116 | -5.38366 | 3.27E-07 | 9.05E-06 | 6.348128 | DOWN |
| AC011513.4 | -2.46427 | 2.243431 | -5.3823 | 3.29E-07 | 9.08E-06 | 6.321998 | DOWN |
| CTC-367J11.1 | -2.31371 | 1.965 | -5.36346 | 3.59E-07 | 9.69E-06 | 6.252775 | DOWN |
| RP11-295M3.4 | -2.55332 | 8.004511 | -5.44513 | 2.47E-07 | 6.96E-06 | 6.250882 | DOWN |
| CASC9 | 5.738144 | 9.391882 | 5.360632 | 3.63E-07 | 9.79E-06 | 6.22362 | UP |
| RP11-93B14.6 | -2.61779 | 4.588963 | -5.37716 | 3.37E-07 | 9.21E-06 | 6.179107 | DOWN |
| RP11-330M19.1 | -2.7975 | 1.61633 | -5.34387 | 3.92E-07 | 1.04E-05 | 6.158065 | DOWN |
| H19 | -2.85613 | 12.81936 | -5.50195 | 1.90E-07 | 5.51E-06 | 6.102485 | DOWN |
| AC131097.4 | -2.63204 | 4.22549 | -5.34867 | 3.83E-07 | 1.02E-05 | 6.073659 | DOWN |
| RP11-1260E13.4 | -2.33292 | 3.483657 | -5.31212 | 4.52E-07 | 1.19E-05 | 5.972292 | DOWN |
| AC096579.13 | -2.61963 | 5.250331 | -5.32687 | 4.23E-07 | 1.12E-05 | 5.915279 | DOWN |
| RP11-328K4.1 | 3.811342 | 5.790054 | 5.254605 | 5.86E-07 | 1.49E-05 | 5.798711 | UP |
| RP11-17M24.1 | -2.73509 | 1.656073 | -5.23731 | 6.33E-07 | 1.58E-05 | 5.707667 | DOWN |
| RP11-8L2.1 | 3.294888 | 5.528648 | 5.201765 | 7.42E-07 | 1.82E-05 | 5.579576 | UP |
| GPR1-AS | 2.200882 | 2.459272 | 5.249077 | 6.01E-07 | 1.52E-05 | 5.571853 | UP |
| RP11-115H13.1 | -3.68566 | 4.109604 | -5.24183 | 6.21E-07 | 1.56E-05 | 5.569372 | DOWN |
| RP5-1065O2.4 | -2.84808 | 1.617344 | -5.20062 | 7.46E-07 | 1.82E-05 | 5.548178 | DOWN |
| RP5-866L20.1 | -2.5083 | 3.216413 | -5.19546 | 7.63E-07 | 1.85E-05 | 5.476822 | DOWN |
| LINC00628 | -2.30764 | 1.878707 | -5.15802 | 9.02E-07 | 2.12E-05 | 5.37759 | DOWN |
| RP11-519G16.5 | -2.18256 | 3.321395 | -5.16249 | 8.84E-07 | 2.09E-05 | 5.347169 | DOWN |
| CTD-2554C21.2 | -2.54586 | 3.876857 | -5.15286 | 9.22E-07 | 2.16E-05 | 5.258447 | DOWN |
| CTA-384D8.35 | 2.714902 | 8.561673 | 5.158929 | 8.98E-07 | 2.12E-05 | 5.25749 | UP |
| LINC00278 | -2.99637 | 3.864742 | -5.15614 | 9.09E-07 | 2.13E-05 | 5.250016 | DOWN |
| RP11-149I9.2 | -2.16415 | 2.153781 | -5.11989 | 1.07E-06 | 2.46E-05 | 5.214819 | DOWN |
| RP11-573D15.2 | 2.25045 | 4.236905 | 5.100378 | 1.16E-06 | 2.66E-05 | 5.147326 | UP |
| PGM5-AS1 | -2.38183 | 1.383676 | -5.09569 | 1.19E-06 | 2.70E-05 | 5.126365 | DOWN |
| RP11-56L13.1 | -2.82141 | 1.861706 | -5.09078 | 1.21E-06 | 2.74E-05 | 5.078375 | DOWN |
| RP11-338H14.1 | 2.174721 | 3.310722 | 5.099822 | 1.17E-06 | 2.66E-05 | 5.048637 | UP |
| CTA-384D8.34 | 2.517247 | 4.463963 | 5.064804 | 1.36E-06 | 3.06E-05 | 4.991833 | UP |
| AC098617.1 | -2.48824 | 2.058504 | -5.05367 | 1.43E-06 | 3.20E-05 | 4.934494 | DOWN |
| RP4-813D12.3 | -2.31892 | 3.850201 | -5.03013 | 1.58E-06 | 3.50E-05 | 4.751697 | DOWN |
| RP11-909N17.2 | 2.638807 | 3.763774 | 5.018283 | 1.67E-06 | 3.66E-05 | 4.714689 | UP |
| RP11-526F3.1 | -2.8346 | 1.730755 | -4.99723 | 1.83E-06 | 3.94E-05 | 4.695661 | DOWN |
| HOXC13-AS | 4.729889 | 6.996888 | 4.968198 | 2.07E-06 | 4.39E-05 | 4.603108 | UP |
| LINC01322 | 4.094956 | 5.284743 | 4.980472 | 1.97E-06 | 4.19E-05 | 4.570339 | UP |
| RP11-509A17.3 | 2.272457 | 2.685239 | 5.010079 | 1.73E-06 | 3.75E-05 | 4.564093 | UP |
| RP4-598P13.1 | 2.252731 | 3.109457 | 4.989382 | 1.89E-06 | 4.05E-05 | 4.525647 | UP |
| LINC01518 | 3.233328 | 3.936786 | 4.979737 | 1.97E-06 | 4.20E-05 | 4.465352 | UP |
| AQP4-AS1 | -2.65032 | 2.842128 | -4.94137 | 2.33E-06 | 4.89E-05 | 4.428417 | DOWN |
| RP11-297P16.4 | 5.814363 | 6.479451 | 4.960062 | 2.15E-06 | 4.54E-05 | 4.39159 | UP |
| CTA-363E6.2 | -2.62353 | 2.14299 | -4.88941 | 2.91E-06 | 5.93E-05 | 4.245997 | DOWN |
| AC002398.12 | -2.58356 | 1.496627 | -4.8756 | 3.09E-06 | 6.24E-05 | 4.208529 | DOWN |
| HOXB-AS2 | -2.21987 | 3.932901 | -4.86808 | 3.19E-06 | 6.41E-05 | 4.081423 | DOWN |
| CTA-392E5.1 | 3.850729 | 4.320424 | 4.875388 | 3.09E-06 | 6.24E-05 | 3.995097 | UP |
| AC091814.3 | 3.394513 | 5.051591 | 4.821804 | 3.89E-06 | 7.63E-05 | 3.975046 | UP |
| RP11-346D19.1 | 2.621492 | 4.734296 | 4.812701 | 4.04E-06 | 7.86E-05 | 3.969617 | UP |
| RP11-510M2.5 | -2.32487 | 1.959126 | -4.80095 | 4.25E-06 | 8.21E-05 | 3.907838 | DOWN |
| LINC00941 | 3.599086 | 7.306229 | 4.79531 | 4.35E-06 | 8.30E-05 | 3.873496 | UP |
| RP11-398B16.2 | -2.63402 | 2.880407 | -4.79745 | 4.31E-06 | 8.30E-05 | 3.838166 | DOWN |
| RP11-680C21.1 | -2.88151 | 3.742529 | -4.8069 | 4.14E-06 | 8.04E-05 | 3.813599 | DOWN |
| CTA-398F10.2 | -2.20989 | 3.490907 | -4.7892 | 4.46E-06 | 8.46E-05 | 3.793057 | DOWN |
| CTD-2357A8.3 | 3.706969 | 6.144717 | 4.753983 | 5.18E-06 | 9.61E-05 | 3.739567 | UP |
| LINC01456 | 2.216508 | 2.467971 | 4.796955 | 4.32E-06 | 8.30E-05 | 3.677493 | UP |
| RP11-197K6.1 | 4.351091 | 4.775046 | 4.793555 | 4.38E-06 | 8.32E-05 | 3.665849 | UP |
| NAALADL2-AS2 | 2.597955 | 3.558188 | 4.775283 | 4.74E-06 | 8.87E-05 | 3.64675 | UP |
| RP5-984P4.6 | 2.353569 | 2.736264 | 4.776374 | 4.71E-06 | 8.84E-05 | 3.602932 | UP |
| RP11-993B23.3 | 2.975536 | 6.113996 | 4.7077 | 6.29E-06 | 0.000113 | 3.548599 | UP |
| AC002511.2 | -2.30627 | 3.300114 | -4.72119 | 5.95E-06 | 0.000108 | 3.525573 | DOWN |
| HOXA11-AS | 3.461449 | 5.390227 | 4.700202 | 6.50E-06 | 0.000115 | 3.516216 | UP |
| RP11-12L8.1 | -2.19527 | 1.34218 | -4.68679 | 6.87E-06 | 0.000121 | 3.469037 | DOWN |
| CTD-2021H9.3 | 4.806339 | 6.092643 | 4.687804 | 6.84E-06 | 0.000121 | 3.430617 | UP |
| HAGLROS | 2.61129 | 6.4675 | 4.682083 | 7.01E-06 | 0.000123 | 3.42088 | UP |
| RP11-96B2.1 | -2.25112 | 4.55787 | -4.70439 | 6.38E-06 | 0.000114 | 3.379384 | DOWN |
| RP11-1020M18.10 | 3.138322 | 4.368029 | 4.679589 | 7.08E-06 | 0.000124 | 3.339775 | UP |
| CTD-2066L21.3 | 3.297967 | 4.729924 | 4.668153 | 7.43E-06 | 0.000129 | 3.310828 | UP |
| RP11-706C16.7 | -2.50795 | 4.178754 | -4.68201 | 7.01E-06 | 0.000123 | 3.301872 | DOWN |
| RP11-218E20.3 | 2.710646 | 7.293289 | 4.636494 | 8.47E-06 | 0.000144 | 3.198505 | UP |
| LINC00284 | -2.85238 | 3.488315 | -4.64554 | 8.16E-06 | 0.000139 | 3.18612 | DOWN |
| RP11-42I10.1 | 2.16943 | 5.867426 | 4.606984 | 9.57E-06 | 0.000159 | 3.138117 | UP |
| LINC00460 | 3.690131 | 5.113426 | 4.606645 | 9.59E-06 | 0.000159 | 3.073412 | UP |
| RP11-281A20.1 | -2.60829 | 2.790988 | -4.60165 | 9.79E-06 | 0.000162 | 3.069005 | DOWN |
| RP11-69G7.1 | 4.972184 | 5.926968 | 4.609152 | 9.49E-06 | 0.000159 | 3.050119 | UP |
| CTC-338M12.9 | 2.325993 | 2.83419 | 4.612834 | 9.34E-06 | 0.000157 | 2.957461 | UP |
| RP11-114H23.1 | -2.32987 | 4.911867 | -4.59908 | 9.89E-06 | 0.000163 | 2.93258 | DOWN |
| AC012363.4 | 2.529942 | 3.120578 | 4.604554 | 9.67E-06 | 0.00016 | 2.929226 | UP |
| ATP1B3-AS1 | 2.370474 | 5.052315 | 4.540884 | 1.26E-05 | 0.000199 | 2.908474 | UP |
| RP11-542G1.1 | 2.304951 | 3.381637 | 4.575354 | 1.09E-05 | 0.000176 | 2.903974 | UP |
| RP11-114H23.2 | -2.34598 | 3.648693 | -4.55516 | 1.19E-05 | 0.000189 | 2.846329 | DOWN |
| LINC01096 | 2.167994 | 4.385893 | 4.511761 | 1.42E-05 | 0.000219 | 2.792418 | UP |
| RP11-266O8.1 | -2.1931 | 2.907935 | -4.51458 | 1.40E-05 | 0.000217 | 2.743607 | DOWN |
| RP11-107M16.2 | -2.28252 | 3.635689 | -4.52546 | 1.34E-05 | 0.000209 | 2.736 | DOWN |
| RP11-78C3.1 | 2.164331 | 2.737524 | 4.543989 | 1.24E-05 | 0.000197 | 2.688655 | UP |
| LINC01561 | 2.615984 | 3.891243 | 4.510063 | 1.43E-05 | 0.00022 | 2.679922 | UP |
| HOXA10-AS | 2.328544 | 5.443582 | 4.454981 | 1.78E-05 | 0.000265 | 2.572175 | UP |
| RP11-366F6.2 | 2.927884 | 3.094497 | 4.48931 | 1.55E-05 | 0.000236 | 2.452777 | UP |
| CASC20 | 3.119015 | 4.000076 | 4.45334 | 1.79E-05 | 0.000266 | 2.404104 | UP |
| RP11-323C15.2 | -2.47738 | 3.471035 | -4.43725 | 1.91E-05 | 0.00028 | 2.398691 | DOWN |
| RP11-734K21.5 | -2.4829 | 5.761057 | -4.47984 | 1.61E-05 | 0.000243 | 2.397018 | DOWN |
| LINC00355 | 2.830881 | 3.209145 | 4.44668 | 1.84E-05 | 0.00027 | 2.303235 | UP |
| RP11-713N11.6 | -2.18563 | 2.478475 | -4.35554 | 2.66E-05 | 0.000366 | 2.163329 | DOWN |
| RP11-625H11.2 | 2.266637 | 2.87776 | 4.399686 | 2.23E-05 | 0.000317 | 2.139663 | UP |
| CTD-2591A6.2 | 2.196588 | 2.467266 | 4.40652 | 2.17E-05 | 0.00031 | 2.138225 | UP |
| RP11-445F12.1 | 3.072844 | 4.045715 | 4.378896 | 2.42E-05 | 0.000341 | 2.126124 | UP |
| DLX6-AS1 | 2.943582 | 7.577858 | 4.344814 | 2.77E-05 | 0.00038 | 2.068091 | UP |
| LINC00887 | -2.25055 | 6.079605 | -4.37193 | 2.49E-05 | 0.000348 | 1.973383 | DOWN |
| TTTY14 | -2.94658 | 4.940035 | -4.35739 | 2.64E-05 | 0.000364 | 1.964895 | DOWN |
| AC009410.1 | 3.113934 | 4.013411 | 4.339219 | 2.84E-05 | 0.000385 | 1.95764 | UP |
| RP11-225N10.1 | -2.17653 | 3.136199 | -4.30089 | 3.30E-05 | 0.00044 | 1.920874 | DOWN |
| RP11-493L12.3 | 2.400069 | 3.647482 | 4.310607 | 3.18E-05 | 0.000427 | 1.913032 | UP |
| RP11-1C8.4 | 2.471223 | 3.817179 | 4.297347 | 3.35E-05 | 0.000445 | 1.897618 | UP |
| RP11-23D24.2 | -2.21732 | 5.593156 | -4.33987 | 2.83E-05 | 0.000385 | 1.887851 | DOWN |
| CTD-2566J3.1 | -2.17411 | 1.646098 | -4.27248 | 3.69E-05 | 0.000488 | 1.882239 | DOWN |
| TEX41 | 3.212229 | 6.367687 | 4.263317 | 3.83E-05 | 0.000499 | 1.84764 | UP |
| RP11-110H1.8 | 2.674812 | 3.548103 | 4.310007 | 3.18E-05 | 0.000428 | 1.828477 | UP |
| CTD-2231H16.1 | 2.992804 | 6.864816 | 4.262828 | 3.83E-05 | 0.000499 | 1.810791 | UP |
| RP11-190J1.3 | 2.38655 | 2.808941 | 4.300608 | 3.30E-05 | 0.00044 | 1.746603 | UP |
| RP11-320N7.2 | -2.16831 | 1.335775 | -4.20837 | 4.75E-05 | 0.000597 | 1.65363 | DOWN |
| PCAT7 | 3.065691 | 6.25683 | 4.199 | 4.92E-05 | 0.000617 | 1.609648 | UP |
| RP11-357H14.17 | 4.96777 | 6.711549 | 4.182038 | 5.26E-05 | 0.00065 | 1.542394 | UP |
| RP11-831A10.1 | -2.75874 | 3.129416 | -4.19143 | 5.07E-05 | 0.000633 | 1.483799 | DOWN |
| RP11-145A3.1 | 3.071828 | 6.020998 | 4.16034 | 5.72E-05 | 0.0007 | 1.480412 | UP |
| RP11-123B3.2 | -2.22581 | 2.844271 | -4.16284 | 5.66E-05 | 0.000696 | 1.426951 | DOWN |
| RP11-863P13.3 | 2.658335 | 6.433776 | 4.126111 | 6.52E-05 | 0.000776 | 1.322848 | UP |
| CTD-2227E11.1 | 2.202688 | 4.507596 | 4.112278 | 6.88E-05 | 0.000803 | 1.312651 | UP |
| HAND2-AS1 | -2.18386 | 3.213963 | -4.12583 | 6.53E-05 | 0.000776 | 1.274646 | DOWN |
| RP11-417E7.1 | 3.224285 | 7.701456 | 4.123397 | 6.59E-05 | 0.00078 | 1.265954 | UP |
| RP11-356K23.1 | -2.46509 | 2.719574 | -4.11183 | 6.89E-05 | 0.000803 | 1.241465 | DOWN |
| RP5-884M6.1 | 3.884356 | 6.620225 | 4.085827 | 7.61E-05 | 0.000871 | 1.212475 | UP |
| AC084193.1 | -2.42322 | 1.505397 | -4.08814 | 7.54E-05 | 0.000868 | 1.210834 | DOWN |
| LINC01249 | 2.583633 | 2.99583 | 4.143944 | 6.09E-05 | 0.000734 | 1.169623 | UP |
| CTC-499J9.1 | 3.351267 | 5.712663 | 4.043842 | 8.93E-05 | 0.001004 | 1.066488 | UP |
| RP11-680H20.2 | 2.469885 | 4.019357 | 4.0424 | 8.98E-05 | 0.001008 | 1.010737 | UP |
| RP5-1011O1.2 | 2.348373 | 3.750728 | 4.049835 | 8.73E-05 | 0.000984 | 0.994709 | UP |
| ADAM20P1 | 2.263869 | 5.205694 | 3.998367 | 0.000106 | 0.001155 | 0.907105 | UP |
| RP11-110A12.2 | 4.232553 | 5.711626 | 4.01233 | 0.000101 | 0.001107 | 0.904102 | UP |
| RP11-180K7.1 | -2.22147 | 2.075033 | -4.00331 | 0.000104 | 0.001139 | 0.900397 | DOWN |
| AP001065.15 | 3.021741 | 3.830285 | 4.021721 | 9.71E-05 | 0.001075 | 0.828342 | UP |
| RP11-255P5.3 | -2.17963 | 1.103436 | -3.9562 | 0.000124 | 0.001324 | 0.757811 | DOWN |
| MIAT | 2.309767 | 9.582722 | 4.017252 | 9.87E-05 | 0.00109 | 0.675677 | UP |
| AP001610.9 | -2.17593 | 1.428807 | -3.93441 | 0.000135 | 0.001414 | 0.675043 | DOWN |
| RP11-1038A11.3 | 2.246262 | 7.241551 | 3.96308 | 0.000121 | 0.001296 | 0.662066 | UP |
| CTD-2377D24.6 | 3.438671 | 5.078452 | 3.939851 | 0.000132 | 0.001393 | 0.659366 | UP |
| LSAMP-AS1 | 2.224739 | 3.693142 | 3.943305 | 0.00013 | 0.001384 | 0.650728 | UP |
| IGF2BP2-AS1 | 2.301451 | 4.535972 | 3.882656 | 0.000163 | 0.001655 | 0.505209 | UP |
| RP11-133L19.3 | -2.70556 | 2.121474 | -3.86767 | 0.000172 | 0.001734 | 0.396513 | DOWN |
| RP4-594A5.1 | 2.20776 | 4.022145 | 3.859034 | 0.000178 | 0.001775 | 0.382219 | UP |
| AC006460.2 | 2.271994 | 4.092543 | 3.853724 | 0.000181 | 0.001801 | 0.378213 | UP |
| RP11-567G11.1 | 2.290672 | 5.215079 | 3.830163 | 0.000198 | 0.001926 | 0.327508 | UP |
| ARHGEF26-AS1 | -2.19562 | 5.411919 | -3.88779 | 0.00016 | 0.001631 | 0.261949 | DOWN |
| AC098973.2 | 4.422976 | 7.072032 | 3.799926 | 0.000221 | 0.002118 | 0.219097 | UP |
| RP11-758M4.4 | 5.273119 | 6.9781 | 3.805162 | 0.000217 | 0.002088 | 0.217117 | UP |
| LINC01436 | 3.172993 | 6.215364 | 3.793285 | 0.000226 | 0.002156 | 0.193433 | UP |
| MIR133A1HG | -2.61802 | 2.541337 | -3.78356 | 0.000234 | 0.002216 | 0.098682 | DOWN |
| FOXD3-AS1 | 2.844521 | 5.812054 | 3.752188 | 0.000262 | 0.002428 | 0.060084 | UP |
| RP11-95M15.1 | 2.735474 | 4.86581 | 3.725953 | 0.000288 | 0.002628 | -0.02827 | UP |
| ZFPM2-AS1 | 3.953842 | 6.897599 | 3.725823 | 0.000288 | 0.002628 | -0.03206 | UP |
| CTA-384D8.31 | 3.520352 | 6.574901 | 3.712706 | 0.000302 | 0.002741 | -0.07905 | UP |
| TSPEAR-AS1 | 3.106997 | 4.953178 | 3.70735 | 0.000308 | 0.002781 | -0.10197 | UP |
| SNAP25-AS1 | 2.377059 | 4.191223 | 3.709091 | 0.000306 | 0.002769 | -0.10338 | UP |
| TRPM2-AS | 3.270335 | 6.837061 | 3.696756 | 0.00032 | 0.002855 | -0.15806 | UP |
| MNX1-AS1 | 2.427252 | 4.169215 | 3.652721 | 0.000374 | 0.003228 | -0.31431 | UP |
| CTC-573N18.1 | 2.249632 | 3.800078 | 3.660416 | 0.000364 | 0.003159 | -0.33268 | UP |
| LINC00518 | 2.175346 | 3.442272 | 3.666759 | 0.000356 | 0.003103 | -0.35148 | UP |
| P3H2-AS1 | 2.683367 | 4.554467 | 3.606876 | 0.00044 | 0.003672 | -0.44161 | UP |
| LINC01160 | 3.68894 | 6.648499 | 3.597767 | 0.000454 | 0.003761 | -0.45134 | UP |
| LINC00626 | 2.845147 | 4.082379 | 3.610557 | 0.000434 | 0.003641 | -0.49349 | UP |
| RP11-255H23.4 | -2.20844 | 2.741246 | -3.59125 | 0.000464 | 0.003828 | -0.52981 | DOWN |
| CTD-2587H24.5 | 2.174147 | 3.333949 | 3.572452 | 0.000496 | 0.004024 | -0.68076 | UP |
| RP11-488I20.8 | 2.427448 | 3.093534 | 3.589506 | 0.000467 | 0.003848 | -0.73134 | UP |
| RP11-553A10.1 | 2.776837 | 5.095144 | 3.505105 | 0.000626 | 0.004821 | -0.73597 | UP |
| RP11-481J2.2 | 2.253861 | 5.194353 | 3.49992 | 0.000637 | 0.004896 | -0.75165 | UP |
| AC108676.1 | 3.88324 | 7.057153 | 3.473259 | 0.000698 | 0.005248 | -0.85718 | UP |
| APCDD1L-AS1 | 3.035131 | 6.564381 | 3.467289 | 0.000712 | 0.005317 | -0.88433 | UP |
| LINC00261 | -2.16787 | 2.013419 | -3.4603 | 0.000729 | 0.00542 | -0.90144 | DOWN |
| AC156455.1 | 2.480904 | 5.418453 | 3.388827 | 0.000928 | 0.006592 | -1.10007 | UP |
| RP3-323A16.1 | 2.677111 | 6.282545 | 3.379604 | 0.000957 | 0.006769 | -1.16109 | UP |
| AC007128.1 | 2.446012 | 5.156711 | 3.343258 | 0.00108 | 0.00745 | -1.23384 | UP |
| RP11-21B23.2 | -2.16455 | 5.593939 | -3.43317 | 0.000799 | 0.005827 | -1.24634 | DOWN |
| RP1-101G11.3 | 2.511901 | 4.259568 | 3.349394 | 0.001058 | 0.007336 | -1.25941 | UP |
| LINC00944 | 2.304634 | 5.559496 | 3.307965 | 0.001214 | 0.008146 | -1.35456 | UP |
| AC011738.4 | 2.449825 | 5.589838 | 3.284187 | 0.001312 | 0.008678 | -1.42129 | UP |
| TSPEAR-AS2 | 3.177675 | 5.33632 | 3.263164 | 0.001406 | 0.009141 | -1.48021 | UP |
| LINC01559 | -2.52707 | 7.68421 | -3.35948 | 0.001023 | 0.007158 | -1.67148 | DOWN |
| RP11-221N13.3 | 2.484218 | 4.652493 | 3.171036 | 0.001892 | 0.011643 | -1.75971 | UP |
| LINC00520 | 2.503882 | 6.308283 | 3.124902 | 0.002191 | 0.013089 | -1.92614 | UP |
| AC073957.15 | 2.519586 | 3.948313 | 3.108823 | 0.002305 | 0.013624 | -2.03322 | UP |
| ELFN1-AS1 | 2.551485 | 4.119867 | 3.078996 | 0.002531 | 0.014676 | -2.07781 | UP |
| AL133493.2 | 3.038867 | 7.320957 | 3.081342 | 0.002513 | 0.014589 | -2.09667 | UP |
| RP11-451G4.2 | -2.36527 | 1.923762 | -3.05897 | 0.002695 | 0.015442 | -2.1008 | DOWN |
| AF003625.3 | 2.559943 | 3.766794 | 3.092831 | 0.002424 | 0.014167 | -2.11398 | UP |
| AC007249.3 | 2.291114 | 4.351996 | 2.964188 | 0.003607 | 0.019569 | -2.33787 | UP |
| FEZF1-AS1 | 3.273201 | 6.405103 | 2.963595 | 0.003614 | 0.019593 | -2.34953 | UP |
| AC128709.3 | 2.253381 | 4.990511 | 2.871827 | 0.004762 | 0.024143 | -2.57548 | UP |
| AC009264.1 | 2.845133 | 5.34933 | 2.861216 | 0.004915 | 0.024721 | -2.60547 | UP |
| RP11-567M16.1 | 2.602555 | 6.058509 | 2.845306 | 0.005152 | 0.025653 | -2.67803 | UP |
| AFAP1-AS1 | 4.060374 | 8.414046 | 2.85105 | 0.005065 | 0.025355 | -2.74473 | UP |
| LINC00668 | 3.405619 | 7.421322 | 2.743999 | 0.006922 | 0.032292 | -2.99176 | UP |
| RP11-76C10.5 | 2.668818 | 4.04614 | 2.731257 | 0.00718 | 0.033136 | -3.02685 | UP |
| RP11-284F21.9 | 2.40802 | 6.311894 | 2.622789 | 0.009757 | 0.041633 | -3.27478 | UP |
| CTD-2354A18.1 | 2.320566 | 3.865904 | 2.439975 | 0.016027 | 0.060188 | -3.76513 | UP |
| LINC01468 | 2.493182 | 5.577684 | 2.32547 | 0.021585 | 0.075041 | -3.916 | UP |
| RP4-694A7.2 | 2.315546 | 5.879221 | 2.306434 | 0.022658 | 0.07794 | -3.98239 | UP |
| RP11-54H7.4 | 2.365367 | 10.30636 | 2.40961 | 0.017361 | 0.064153 | -4.13426 | UP |
