# Supplementary material for: PI3K/Akt signalling pathway-associated long noncoding RNA signature predicts the prognosis of laryngeal cancer patients
Source: Sci Rep. 2023 Sep 7;13:14764. doi: 10.1038/s41598-023-41927-3 (PMC10485045; doi:10.1038/s41598-023-41927-3)
Supplement: Supplementary file 7 — Supplementary Information 4. [file 41598_2023_41927_MOESM7_ESM.pdf]

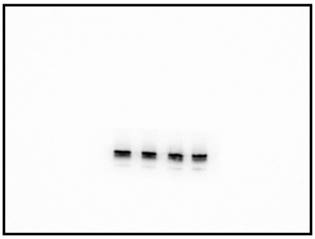

AKT AMC-HN-8 1.0sec

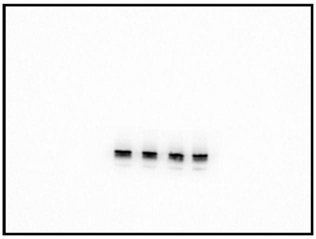

AKT AMC-HN-8 1.5sec

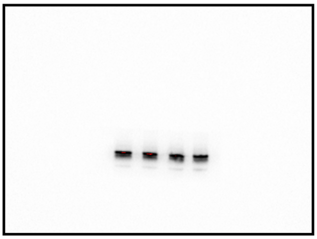

AKT AMC-HN-8 2.5sec

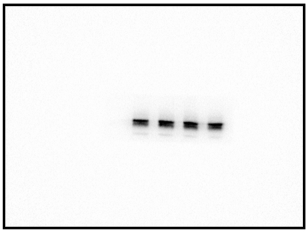

AKT TU177 1.0sec

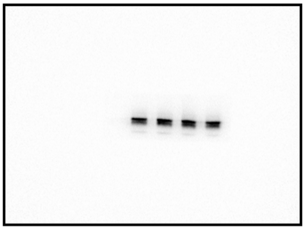

AKT TU177 1.8sec

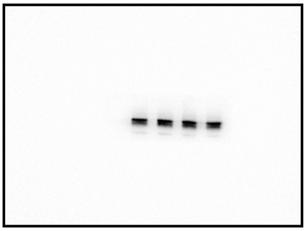

AKT TU177 2.6sec

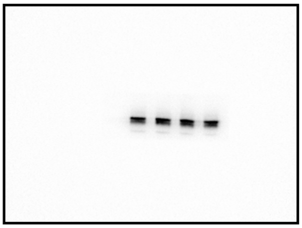

AKT TU177 3.2sec

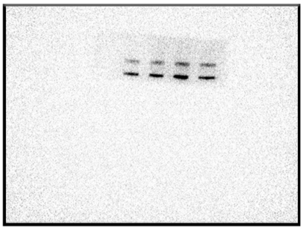

P-AKT AMC-HN-8 1.0sec

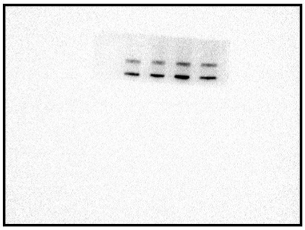

P-AKT AMC-HN-8 4.0sec

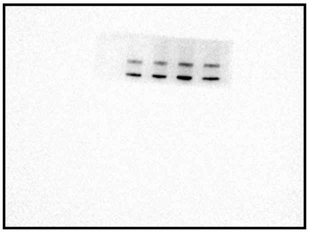

P-AKT AMC-HN-8 7.0sec

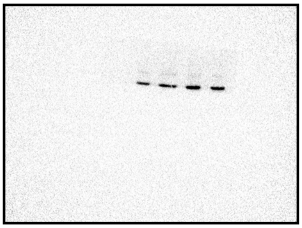

P-AKT TU177 1.0sec

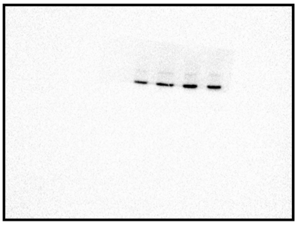

P-AKT TU177 4.0sec

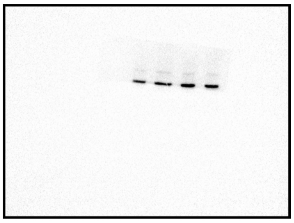

P-AKT TU177 7.0sec

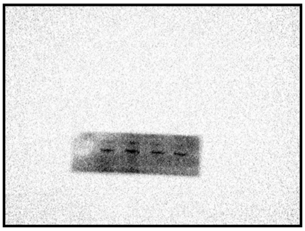

PI3K AMC-HN-8 1.0sec

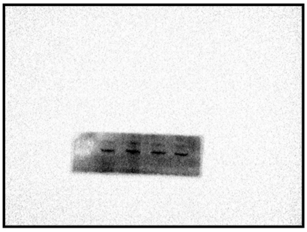

PI3K AMC-HN-8 3.0sec

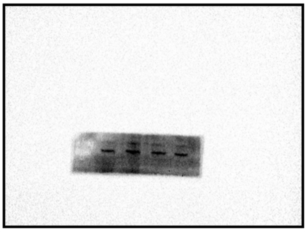

PI3K AMC-HN-8 6.0sec

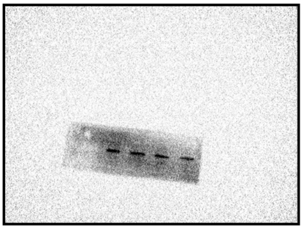

PI3K TU177 1.0sec

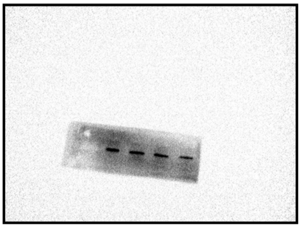

PI3K TU177 4.0sec

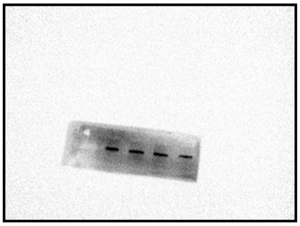

PI3K TU177 6.0sec

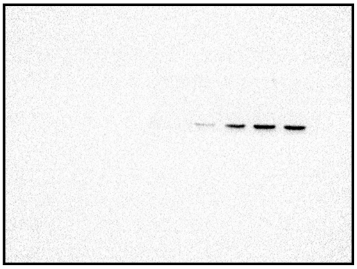

P-PI3K AMC-HN-8 1.0sec

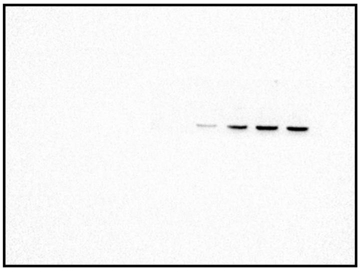

P-PI3K AMC-HN-8 4.0sec

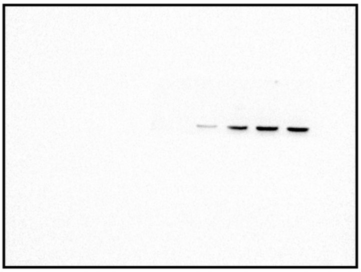

P-PI3K AMC-HN-8 7.0sec

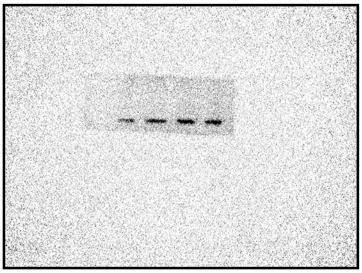

P-PI3K TU177 1.0sec

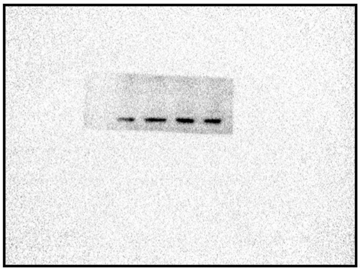

P-PI3K TU177 4.0sec

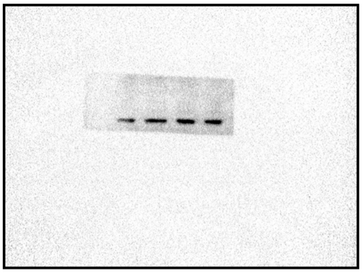

P-PI3K TU177 8.0sec
